# Recon2Neo4j: applying graph database technologies for managing comprehensive genome-scale networks

**DOI:** 10.1093/bioinformatics/btw731

**Published:** 2016-12-30

**Authors:** Irina Balaur, Alexander Mazein, Mansoor Saqi, Artem Lysenko, Christopher J Rawlings, Charles Auffray

**Affiliations:** 1 European Institute for Systems Biology and Medicine (EISBM), CIRI CNRS UMR, CNRS-ENS-UCBL-INSERM, Lyon, France; 2 Rothamsted Research, Harpenden, West Common, Hertfordshire, UK

## Abstract

**Summary:**

The goal of this work is to offer a computational framework for exploring data from the Recon2 human metabolic reconstruction model. Advanced user access features have been developed using the Neo4j graph database technology and this paper describes key features such as efficient management of the network data, examples of the network querying for addressing particular tasks, and how query results are converted back to the Systems Biology Markup Language (SBML) standard format. The Neo4j-based metabolic framework facilitates exploration of highly connected and comprehensive human metabolic data and identification of metabolic subnetworks of interest. A Java-based parser component has been developed to convert query results (available in the JSON format) into SBML and SIF formats in order to facilitate further results exploration, enhancement or network sharing.

**Availability and Implementation:**

The Neo4j-based metabolic framework is freely available from: https://diseaseknowledgebase.etriks.org/metabolic/browser/. The java code files developed for this work are available from the following url: https://github.com/ibalaur/MetabolicFramework.

**Supplementary information:**

Supplementary data are available at *Bioinformatics* online.

## 1 Introduction

Genome-scale consensus models are essential for further advances in Systems Biology and Systems Medicine. Recon2 (Thiele *et al.*, 2013) is the most up-to-date comprehensive community-driven reconstruction of the human metabolic network, with 7440 reactions, 2626 unique metabolites and 1789 proteins included. The Recon2 resource is structured in the Systems Biology Markup Language (SBML) standard format (Hucka *et al.*, 2003) and is publically available (Virtual Metabolic Human, https://vmh.uni.lu/). However, advanced exploration involving associations between multiple concepts (e.g. network neighborhood of particular metabolites, shortest pathways between specific metabolites, proteins and complexes) is challenging for models of the size and complexity of this extensive high quality reconstruction. This study demonstrates that advanced exploration of genome-scale metabolic reconstructions can benefit from an integrated graph representation of the model and associated data.

## 2 Methods

The Recon2 human metabolic reconstruction (in SBML format) was integrated into the Neo4j framework (https://neo4j.com/), which uses a graph database approach. The major concepts involved in the metabolic reactions (metabolites, proteins, complexes and metabolic reaction names) were represented as nodes in the graph database, while the relationships among them (e.g. consumption, production, catalysis) as connecting edges. In addition, the relationships between the compounds (nodes) and the complexes were represented by ‘part of’ edges. Information on the name, the SBO Term identifier and additional details (such as initial concentration, charge, metadata) were stored as attributes (properties) of the nodes. An SBML species was classified as a node of either a metabolite, a protein or a biological complex based on its SBO Term identifier in the Recon2 input file. For the proteins and biological complexes nodes, the UniProt identifier information was also stored as node attributes. When available, data related to biological compartments (including compartment name, meta id, SBO Term id, size, spatial dimensions) were also stored as attributes for every species node. For the metabolic reactions, information such as name, identifier, metadata, notes, the reversibility property, were stored as attributes of the Reaction nodes; for the consumption and production reactions, the stoichiometric relationships were also captured as edge properties. The Neo4j-based metabolic representation of Recon2 is composed of i) nodes: 5063 metabolites, 3567 proteins, 7440 metabolic reactions and 1168 complexes (with 590 protein compounds); and ii) relationships (edges): 15677 consumption, 15863 production, 9982 catalysis and 590 part-of relationships between complexes and their compounds. The data graph model of the Neo4j-based metabolic framework is given in Supplementary Figure S1 (Supplementary file S1).

A parser component was developed to convert the query results from the Neo4j-based metabolic framework in the JavaScript Object Notation (JSON) format to the SBML standard format and the Standard Interchange Format (the SIF format), compatible with well-established environments for biological data management (e.g. Cytoscape (Smoot *et al.*, 2011)) and network sharing (e.g. NDEx (Pratt *et al.*, 2015)). Both the Neo4j-based metabolic framework and the parser component were developed mainly in Java using: the JSBML 1.0 library (Dräger *et al.*, 2011) for managing the SBML files (read and write data, check consistency of the SBML output), the Neo4j Java API to build the Neo4j-based resource and the JSON-simple 1.1.1 library to read information from the JSON files.

## 3 Results

**Fig. 1.**
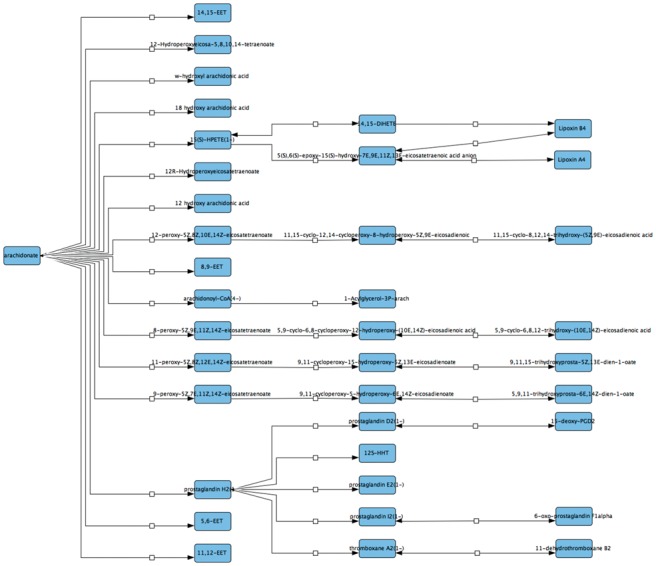
Visualization using CellDesigner (Funahashi et al., 2008) for the arachidonic acid metabolic network identified based on Cypher query 1 (Supplementary file S4)

The developments presented here focus on two major components: i) a Neo4j graph database for the human metabolism data and ii) a Java-based parser for translating the JSON representation of the Neo4j networks into the SBML and SIF formats. The major steps of the overall workflow are illustrated in Supplementary Figure S2 (Supplementary file S2) and are described briefly below. Firstly, the Neo4j-based metabolic framework facilitates exploration and visualization of the human metabolic network. As an example of exploring the newly developed resource using the Neo4j Cypher declarative language, a use-case was developed to identify pathways and subnetworks useful for understanding the metabolism of the arachidonic acid, a metabolite that plays a crucial role in inflammation processes. The metabolic network shown in Supplementary Figure S3 (Supplementary file S3) identifies metabolites and proteins three metabolic reaction steps away from the arachidonic acid (or, in terms of nodes in the graph, the figure illustrates the 6-steps neighborhood of the arachidonic acid node). The network from Supplementary Figure S3 excluded paths with highly connected promiscuous nodes (such as those representing the ‘proton’, ‘H_2_O’, ‘Sodium’), to avoid having all nodes interconnected. A list of examples of Cypher queries for the metabolic framework (including the query for Supplementary Fig. S3) is given in Supplementary file S4. Second, the user can import the Neo4j output file (the JSON format), which contains data on the metabolic subnetwork identified using a Cypher query (e.g. network in Supplementary Fig. S3), into the parser component and choose to export information into the SBML or SIF formats. The SBML output file can be visualized and managed using other tools (e.g. CellDesigner (Funahashi *et al.*, 2008)) or used for further mathematical modeling development. The metabolic subnetwork obtained (the SIF file) can be also explored in Cytoscape or shared among the community through the NDEx platform (Pratt *et al.*, 2015). A visualization of the arachidonate subnetwork using CellDesigner and following manual intervention to improve readability is shown in Figure 1. The output SBML file corresponding to the network in Supplementary Figure S3 is given as Supplementary file S5.

In summary, the developments reported here enable efficient exploration of a human metabolic model by envisioning particular metabolites together with their network neighborhood. Thus, a powerful feature of the Recon2Neo4j framework is facilitating querying and exploration of integrated metabolic data (via the Cypher language, as discussed above), which adds to the functionality provided by other systems biology software, such as cySBML (König *et al.*, 2012). Recon2Neo4j can be easily extended to process other input files available in the SBML standard format, due to the use of the JSBML library functionalities to manage the SBML files, and also to integrate new data types if these become available, due to the use of the graph database approach that presents schema free properties. (More detailed discussions on using the Neo4j environment for the management of biological and biomedical data can be found in e.g. (Lysenko *et al.*, 2016)). As possible future development steps, it would be useful to add more information to the metabolic network, such as synonyms for metabolite names and tissue expression level for proteins (e.g. from the Human Protein Atlas (Uhlen *et al.*, 2010)). Further work is being undertaken to use this newly developed Neo4j-based data integration framework to identify functional modules in disease-specific network reconstruction (e.g. Parkinson disease map, cancer specific disease map).

## Supplementary Material

Supplementary Data
