# Supplementary material for: Recon2Neo4j: applying graph database technologies for managing comprehensive genome-scale networks
Source: Bioinformatics. 2016 Dec 30;33(7):1096–8. doi: 10.1093/bioinformatics/btw731 (PMC5408918; doi:10.1093/bioinformatics/btw731)
Supplement: Supplementary Data [file btw731_supp.zip › Supplementary file 3 - Subnetwork of metabolic reactions (Example).docx]

*Supplementary file 3 - Subnetwork of metabolic reactions, 6-steps further from the arachidonic acid metabolite (Neo4j browser output for the Cypher query 1, Supplementary file 4)*

*
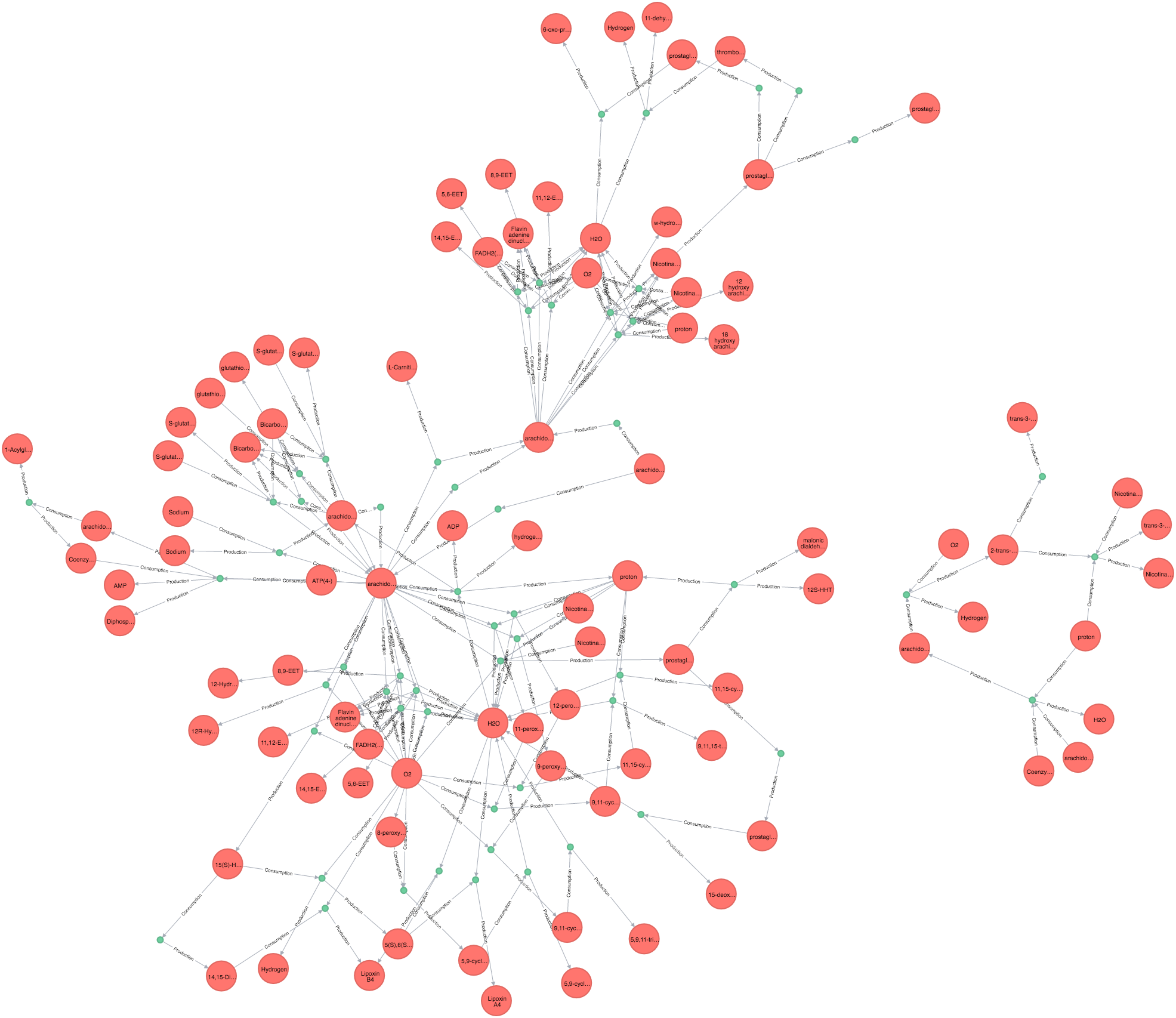
*

**Fig. S3. Subnetwork of metabolic reactions, 6-steps further from the arachidonic acid metabolite**

Nodes: metabolite (red) and reaction (green); edges: consumption, production. The developed parser allows the sub network (the query results) to be edited and further annotated in e.g. CellDesigner (Funahashi et al., 2008) and shared among community using e.g. the NDEx framework (Pratt et al., 2015)).
