# Supplementary material for: Recon2Neo4j: applying graph database technologies for managing comprehensive genome-scale networks
Source: Bioinformatics. 2016 Dec 30;33(7):1096–8. doi: 10.1093/bioinformatics/btw731 (PMC5408918; doi:10.1093/bioinformatics/btw731)
Supplement: Supplementary Data [file btw731_supp.zip › Supplementary file 4 ΓÇô Examples of Cypher queries for the metabolic framework.docx]

*Supplementary file 4 – Examples of Cypher queries for the metabolic framework*

1. Identify all chains of maximum 2 metabolic reactions starting from the arachidonate that do not include metabolites involved in a large number of metabolic reactions (such as proton, H2), Sodium etc..). This Cypher query corresponds to Figure 1 (main paper) and Figure 2 (Supplementary file 1).

**MATCH** path=((m1:Metabolite{MetaboliteName:'arachidonate'})-[r*..6]->(m:Metabolite))

**WHERE** not((m)-->()) and none (n **IN** nodes(path) **WHERE** "Metabolite" **in** labels(n) and n.MetaboliteName **in** ["proton", "H2O", "Sodium", "Coenzyme A", "Nicotinamide adenine dinucleotide phosphate", "Nicotinamide adenine dinucleotide phosphate - reduced", "ATP(4-)", "O2", "Bicarbonate", "Nicotinamide adenine dinucleotide", "hydrogenphosphate", "Nicotinamide adenine dinucleotide - reduced", "ADP", "water","L-threonate","nitrosoperoxycarbonate","S-glutathionyl-ethacrynic-acid","S-glutathionyl-2-4-dinitrobenzene","glutathionyl-leuc4","glycoursodeoxycholate","sulfochenodeoxycholate","tauroursodeoxycholate","2,6-dimethyl heptanoylcoa","5-Formamido-1-(5-phospho-D-ribosyl)imidazole-4-carboxamide","hypothiocyanite","ursodeoxycholate","sulfoglycolithocholate(2-)","palmitoleoyl-Carnitine","reverse-triiodthyronine","carbamoyl phosphate(2-)","S-glutathionyl-2-4-dinitrobenzene","taurolithocholate","glycolithocholate","alloxan","N-acetyl-5-methoxykynuramine","Hydrogen peroxide","FADH2(2-)","ammonium","Flavin adenine dinucleotide oxidized","linoleic-Carnitine","Hydrogen","IMP","cholesterol ester","Ammonium","CO2","R group 2 Coenzyme A","one carbon unit","carbon dioxide","ammonium","FADH-redox-potential","malonic dialdehyde","Flavin adenine dinucleotide oxidized","GDP","Cyanate","L-Carnitine","Orotate","GDP(3-)","AMP","Diphosphate"])

**OPTIONAL** **MATCH** (rr:Reaction)-[cp]-(met:Metabolite) **WHERE** (rr **IN** nodes(path)) and met.MetaboliteName **in** ["proton", "H2O", "Sodium", "Coenzyme A", "Nicotinamide adenine dinucleotide phosphate", "Nicotinamide adenine dinucleotide phosphate - reduced", "ATP(4-)", "O2", "Bicarbonate", "Nicotinamide adenine dinucleotide", "hydrogenphosphate", "Nicotinamide adenine dinucleotide - reduced", "ADP", "water","L-threonate","nitrosoperoxycarbonate","S-glutathionyl-ethacrynic-acid","S-glutathionyl-2-4-dinitrobenzene","glutathionyl-leuc4","glycoursodeoxycholate","sulfochenodeoxycholate","tauroursodeoxycholate","2,6-dimethyl heptanoylcoa","5-Formamido-1-(5-phospho-D-ribosyl)imidazole-4-carboxamide","hypothiocyanite","ursodeoxycholate","sulfoglycolithocholate(2-)","palmitoleoyl-Carnitine","reverse-triiodthyronine","carbamoyl phosphate(2-)","S-glutathionyl-2-4-dinitrobenzene","taurolithocholate","glycolithocholate","alloxan","N-acetyl-5-methoxykynuramine","Hydrogen peroxide","FADH2(2-)","ammonium","Flavin adenine dinucleotide oxidized","linoleic-Carnitine","Hydrogen","IMP","cholesterol ester","Ammonium","CO2","R group 2 Coenzyme A","one carbon unit","carbon dioxide","ammonium","FADH-redox-potential","malonic dialdehyde","Flavin adenine dinucleotide oxidized","GDP","Cyanate","L-Carnitine","Orotate","GDP(3-)","AMP","Diphosphate"])

**RETURN** **distinct** path, cp, met

**Additional queries**

1. Count occurrences in metabolic reactions for each metabolite:

**MATCH** (m:Metabolite)-[:Consumption]->(react1:Reaction), (react2:Reaction)-[:Production]->(m)

**WITH** m.MetaboliteName **as** Metabolite, count(**distinct** react1) **as** ReactConsumption, count(**distinct** react2) **AS** ReactProduction

**RETURN** Metabolite, ReactConsumption, ReactProduction, ReactConsumption + ReactProduction **as** TotalReactions

**ORDER** **BY** TotalReactions **DESC**

1. Identify the metabolic reactions starting from the arachidonate metabolite:

**MATCH** ({MetaboliteName:"arachidonate"})-[r1:Consumption]->(react1:Reaction)-[r3:Production]->(m2:Metabolite), ()-[r2:Catalysis]->(react1) **RETURN** r1, r2,r3

1. Identify which are the chains of metabolic reactions linking two key metabolites, (such **as** Leukotriene D4 and arachidonate):

a) The query returns a chain of the shortest length only (shortest path):

**MATCH** path = shortestPath((m1:Metabolite { MetaboliteName:"arachidonate" })-[*..]-(m2:Metabolite{MetaboliteName:"Leukotriene D4"}))

**RETURN** path

b) The query returns multiple chains of the shortest length (shortest paths):

**MATCH** path = allShortestPaths((m1:Metabolite { MetaboliteName:"arachidonate"})-[*..]- (m2:Metabolite{MetaboliteName:" Leukotriene D4"}),)

**RETURN** path

1. Count occurrences in metabolic reactions for each metabolite:

**MATCH** (m:Metabolite)-[:Consumption]->(react1:Reaction), (react2:Reaction)-[:Production]->(m)

**WITH** m.MetaboliteName **as** Metabolite, count(**distinct** react1) **as** ReactConsumption, count(**distinct** react2) **AS** ReactProduction

**RETURN** Metabolite, ReactConsumption, ReactProduction, ReactConsumption + ReactProduction **as** TotalReactions

**ORDER** **BY** TotalReactions **DESC**
