# Supplementary material for: Recon2Neo4j: applying graph database technologies for managing comprehensive genome-scale networks
Source: Bioinformatics. 2016 Dec 30;33(7):1096–8. doi: 10.1093/bioinformatics/btw731 (PMC5408918; doi:10.1093/bioinformatics/btw731)
Supplement: Supplementary Data [file btw731_supp.zip › Supplementary file 5 ΓÇô SMBL representation arachidonate subnetwork.docx]

*Supplementary file 5 – SMBL representation specific to the arachidonic acid metabolic subnetwork (Fig. S3, Supplementary file 3) and to Cypher query 1, (Supplementary file 4)*

Information was parsed from the JSON (Neo4j output format) using the parser component developed (see Methods section).

<?xml version='1.0' encoding='UTF-8' standalone='no'?>

<!-- Created by Neo4jParser version 1.0 on 2016-42-08 at 15:42:53 CEST with JSBML version 1.0-rc1. -->

<sbml xmlns="<http://www.sbml.org/sbml/level2/version4>" level="2" version="4">

<model metaid="bc62c660-2763-430e-b9b8-a290f9f6b32a" id="recon2_subnetwork">

<annotation>

<rdf:RDF xmlns:rdf="<http://www.w3.org/1999/02/22-rdf-syntax-ns#>" xmlns:dc="<http://purl.org/dc/elements/1.1/>" xmlns:vCard="<http://www.w3.org/2001/vcard-rdf/3.0#>">

<rdf:Description rdf:about="#bc62c660-2763-430e-b9b8-a290f9f6b32a">

<dc:creator>

<rdf:Bag>

<rdf:li rdf:parseType="Resource">

<vCard:N rdf:parseType="Resource">

<vCard:Family>EISBM</vCard:Family>

<vCard:Given>EISBM</vCard:Given>

</vCard:N>

<vCard:EMAIL>recon2_neo4j@eisbm.org</vCard:EMAIL>

<vCard:ORG rdf:parseType="Resource">

<vCard:Orgname>EISBM</vCard:Orgname>

</vCard:ORG>

</rdf:li>

</rdf:Bag>

</dc:creator>

</rdf:Description>

</rdf:RDF>

</annotation>

<listOfUnitDefinitions>

<unitDefinition id="mmol_per_gDW_per_hr">

</unitDefinition>

</listOfUnitDefinitions>

<listOfCompartments>

<compartment constant="false" size="1" metaid="_cbd1dd46_c958_4b53_9667_27890b8ac164" sboTerm="SBO:0000290" name="cytoplasm" id="c" spatialDimensions="3"/>

<compartment constant="false" size="1" metaid="_47f3635d_6371_447d_b4a8_68526a2d10a4" sboTerm="SBO:0000290" name="endoplasmic reticulum" id="r" spatialDimensions="3"/>

<compartment constant="false" size="1" metaid="_691bce5a_c492_48fd_95dd_a5f3a62df544" sboTerm="SBO:0000290" name="extracellular space" id="e" spatialDimensions="3"/>

<compartment constant="false" size="1" metaid="_21132531_7891_4e77_b648_11580dd8d2ad" sboTerm="SBO:0000290" name="mitochondrion" id="m" spatialDimensions="3"/>

<compartment constant="false" size="1" metaid="_8e68c920_7ef5_4ae0_8e46_52883b4d82fe" sboTerm="SBO:0000290" name="Golgi apparatus" id="g" spatialDimensions="3"/>

<compartment constant="false" size="1" metaid="_29153f24_fffe_43eb_8a4f_f58d332df102" sboTerm="SBO:0000290" name="peroxisome" id="x" spatialDimensions="3"/>

<compartment constant="false" size="1" metaid="_bb5aca61_894d_4ff8_86c4_aaf96e0307e4" sboTerm="SBO:0000290" name="nucleus" id="n" spatialDimensions="3"/>

<compartment constant="false" size="1" metaid="_25c4654e_a5e4_4427_9721_1c6f579e8ea1" sboTerm="SBO:0000290" name="lysosome" id="l" spatialDimensions="3"/>

</listOfCompartments>

<listOfSpecies>

<species boundaryCondition="false" charge="-1" constant="false" metaid="_metaM_thrnt_c" hasOnlySubstanceUnits="false" sboTerm="SBO:0000247" compartment="c" name="L-threonate" id="M_thrnt_c" initialConcentration="1">

<notes>

<body xmlns="<http://www.w3.org/1999/xhtml>">

<p>FORMULA: C4H7O5</p>

<p>CHARGE: -1</p>

<p>INCHI: InChI=1S/C4H8O5/c5-1-2(6)3(7)4(8)9/h2-3,5-7H,1H2,(H,8,9)/p-1/t2-,3+/m0/s1</p>

<p>EHMN_ABBREVIATION: C01620</p>

</body>

</notes>

</species>

<species boundaryCondition="false" charge="0" constant="false" metaid="_metaM_o2_c" hasOnlySubstanceUnits="false" sboTerm="SBO:0000247" compartment="c" name="O2" id="M_o2_c" initialConcentration="1">

<notes>

<body xmlns="<http://www.w3.org/1999/xhtml>">

<p>FORMULA: O2</p>

<p>CHARGE: 0</p>

<p>HEPATONET_1.0_ABBREVIATION: HC00017</p>

<p>EHMN_ABBREVIATION: C00007</p>

<p>INCHI: InChI=1S/O2/c1-2</p>

</body>

</notes>

</species>

<species boundaryCondition="false" charge="0" constant="false" metaid="_metaM_h2o2_c" hasOnlySubstanceUnits="false" sboTerm="SBO:0000247" compartment="c" name="Hydrogen peroxide" id="M_h2o2_c" initialConcentration="1">

<notes>

<body xmlns="<http://www.w3.org/1999/xhtml>">

<p>FORMULA: H2O2</p>

<p>CHARGE: 0</p>

<p>HEPATONET_1.0_ABBREVIATION: HC00036</p>

<p>EHMN_ABBREVIATION: C00027</p>

<p>INCHI: InChI=1S/H2O2/c1-2/h1-2H</p>

</body>

</notes>

</species>

<species boundaryCondition="false" charge="-1" constant="false" metaid="_metaM_arachd_c" hasOnlySubstanceUnits="false" sboTerm="SBO:0000247" compartment="c" name="arachidonate" id="M_arachd_c" initialConcentration="1">

<notes>

<body xmlns="<http://www.w3.org/1999/xhtml>">

<p>FORMULA: C20H31O2</p>

<p>CHARGE: -1</p>

<p>INCHI: InChI=1S/C20H32O2/c1-2-3-4-5-6-7-8-9-10-11-12-13-14-15-16-17-18-19-20(21)22/h6-7,9-10,12-13,15-16H,2-5,8,11,14,17-19H2,1H3,(H,21,22)/p-1/b7-6-,10-9-,13-12-,16-15-</p>

</body>

</notes>

</species>

<species boundaryCondition="false" charge="-1" constant="false" metaid="_metaM_15HPET_c" hasOnlySubstanceUnits="false" sboTerm="SBO:0000247" compartment="c" name="15(S)-HPETE(1-)" id="M_15HPET_c" initialConcentration="1">

<notes>

<body xmlns="<http://www.w3.org/1999/xhtml>">

<p>FORMULA: C20H31O4</p>

<p>CHARGE: -1</p>

<p>INCHI: InChI=1S/C20H32O4/c1-2-3-13-16-19(24-23)17-14-11-9-7-5-4-6-8-10-12-15-18-20(21)22/h4-5,8-11,14,17,19,23H,2-3,6-7,12-13,15-16,18H2,1H3,(H,21,22)/p-1/b5-4-,10-8-,11-9-,17-14+/t19-/m0/s1</p>

<p>EHMN_ABBREVIATION: C05966</p>

</body>

</notes>

</species>

<species boundaryCondition="false" charge="1" constant="false" metaid="_metaM_h_c" hasOnlySubstanceUnits="false" sboTerm="SBO:0000247" compartment="c" name="proton" id="M_h_c" initialConcentration="1">

<notes>

<body xmlns="<http://www.w3.org/1999/xhtml>">

<p>FORMULA: H</p>

<p>CHARGE: 1</p>

<p>INCHI: InChI=1S/p+1/i/hH</p>

</body>

</notes>

</species>

<species boundaryCondition="false" charge="0" constant="false" metaid="_metaM_h2o_c" hasOnlySubstanceUnits="false" sboTerm="SBO:0000247" compartment="c" name="H2O" id="M_h2o_c" initialConcentration="1">

<notes>

<body xmlns="<http://www.w3.org/1999/xhtml>">

<p>FORMULA: H2O</p>

<p>CHARGE: 0</p>

<p>INCHI: InChI=1S/H2O/h1H2</p>

</body>

</notes>

</species>

<species boundaryCondition="false" charge="-1" constant="false" metaid="_metaM_cynt_c" hasOnlySubstanceUnits="false" sboTerm="SBO:0000247" compartment="c" name="Cyanate" id="M_cynt_c" initialConcentration="1">

<notes>

<body xmlns="<http://www.w3.org/1999/xhtml>">

<p>FORMULA: CNO</p>

<p>CHARGE: -1</p>

<p>INCHI: InChI=1S/CHNO/c2-1-3/h3H/p-1</p>

<p>EHMN_ABBREVIATION: C01417</p>

</body>

</notes>

</species>

<species boundaryCondition="false" charge="0" constant="false" metaid="_metaM_CE2011_c" hasOnlySubstanceUnits="false" sboTerm="SBO:0000247" compartment="c" name="hypothiocyanite" id="M_CE2011_c" initialConcentration="1">

<notes>

<body xmlns="<http://www.w3.org/1999/xhtml>">

<p>FORMULA: CHNOS</p>

<p>CHARGE: 0</p>

<p>INCHI: </p>

<p>EHMN_ABBREVIATION: CE2011</p>

</body>

</notes>

</species>

<species boundaryCondition="false" charge="0" constant="false" metaid="_metaM_CE5860_c" hasOnlySubstanceUnits="false" sboTerm="SBO:0000247" compartment="c" name="N-acetyl-5-methoxykynuramine" id="M_CE5860_c" initialConcentration="1">

<notes>

<body xmlns="<http://www.w3.org/1999/xhtml>">

<p>FORMULA: C12H16N2O3</p>

<p>CHARGE: 0</p>

<p>INCHI: </p>

<p>EHMN_ABBREVIATION: CE5860</p>

</body>

</notes>

</species>

<species boundaryCondition="false" charge="0" constant="false" metaid="_metaM_CE0074_c" hasOnlySubstanceUnits="false" sboTerm="SBO:0000247" compartment="c" name="alloxan" id="M_CE0074_c" initialConcentration="1">

<notes>

<body xmlns="<http://www.w3.org/1999/xhtml>">

<p>FORMULA: C4H2N2O4</p>

<p>CHARGE: 0</p>

<p>INCHI: InChI=1/C4H2N2O4/c7-1-2(8)5-4(10)6-3(1)9/h(H2,5,6,8,9,10)</p>

<p>EHMN_ABBREVIATION: CE0074</p>

</body>

</notes>

</species>

<species boundaryCondition="false" charge="-1" constant="false" metaid="_metaM_CE2567_c" hasOnlySubstanceUnits="false" sboTerm="SBO:0000247" compartment="c" name="5(S),6(S)-epoxy-15(S)-hydroxy-7E,9E,11Z,13E-eicosatetraenoic acid anion" id="M_CE2567_c" initialConcentration="1">

<notes>

<body xmlns="<http://www.w3.org/1999/xhtml>">

<p>FORMULA: C20H29O4</p>

<p>CHARGE: -1</p>

<p>INCHI: </p>

<p>EHMN_ABBREVIATION: CE2567</p>

</body>

</notes>

</species>

<species boundaryCondition="false" charge="-1" constant="false" metaid="_metaM_C06315_c" hasOnlySubstanceUnits="false" sboTerm="SBO:0000247" compartment="c" name="Lipoxin B4" id="M_C06315_c" initialConcentration="1">

<notes>

<body xmlns="<http://www.w3.org/1999/xhtml>">

<p>FORMULA: C20H31O5</p>

<p>CHARGE: -1</p>

<p>INCHI: InChI=1S/C20H32O5/c1-2-3-8-14-18(22)19(23)15-10-7-5-4-6-9-12-17(21)13-11-16-20(24)25/h4-7,9-10,12,15,17-19,21-23H,2-3,8,11,13-14,16H2,1H3,(H,24,25)/p-1/b6-4-,7-5+,12-9+,15-10+/t17-,18+,19-/m1/s1</p>

<p>EHMN_ABBREVIATION: C06315</p>

</body>

</notes>

</species>

<species boundaryCondition="false" charge="-1" constant="false" metaid="_metaM_C06314_c" hasOnlySubstanceUnits="false" sboTerm="SBO:0000247" compartment="c" name="Lipoxin A4" id="M_C06314_c" initialConcentration="1">

<notes>

<body xmlns="<http://www.w3.org/1999/xhtml>">

<p>FORMULA: C20H31O5</p>

<p>CHARGE: -1</p>

<p>INCHI: InChI=1S/C20H32O5/c1-2-3-8-12-17(21)13-9-6-4-5-7-10-14-18(22)19(23)15-11-16-20(24)25/h4-7,9-10,13-14,17-19,21-23H,2-3,8,11-12,15-16H2,1H3,(H,24,25)/p-1/b6-4-,7-5+,13-9+,14-10+/t17-,18+,19-/m0/s1</p>

<p>EHMN_ABBREVIATION: C06314</p>

</body>

</notes>

</species>

<species boundaryCondition="false" charge="-1" constant="false" metaid="_metaM_CE7172_c" hasOnlySubstanceUnits="false" sboTerm="SBO:0000247" compartment="c" name="14,15-DiHETE" id="M_CE7172_c" initialConcentration="1">

<notes>

<body xmlns="<http://www.w3.org/1999/xhtml>">

<p>FORMULA: C20H31O4</p>

<p>CHARGE: -1</p>

<p>INCHI: </p>

<p>EHMN_ABBREVIATION: CE7172</p>

</body>

</notes>

</species>

<species boundaryCondition="false" charge="-1" constant="false" metaid="_metaM_12RHPET_c" hasOnlySubstanceUnits="false" sboTerm="SBO:0000247" compartment="c" name="12R-Hydroperoxyeicosatetraenoate" id="M_12RHPET_c" initialConcentration="1">

<notes>

<body xmlns="<http://www.w3.org/1999/xhtml>">

<p>FORMULA: C20H31O4</p>

<p>CHARGE: -1</p>

<p>INCHI: InChI=1/C20H32O4/c1-2-3-4-5-10-13-16-19(24-23)17-14-11-8-6-7-9-12-15-18-20(21)22/h7-11,13-14,17,19,23H,2-6,12,15-16,18H2,1H3,(H,21,22)/b9-7-,11-8-,13-10-,17-14+/t19-/m1/s1</p>

</body>

</notes>

</species>

<species boundaryCondition="false" charge="-1" constant="false" metaid="_metaM_12HPET_c" hasOnlySubstanceUnits="false" sboTerm="SBO:0000247" compartment="c" name="12-Hydroperoxyeicosa-5,8,10,14-tetraenoate" id="M_12HPET_c" initialConcentration="1">

<notes>

<body xmlns="<http://www.w3.org/1999/xhtml>">

<p>FORMULA: C20H31O4</p>

<p>CHARGE: -1</p>

<p>INCHI: InChI=1/C20H32O4/c1-2-3-4-5-10-13-16-19(24-23)17-14-11-8-6-7-9-12-15-18-20(21)22/h7-11,13-14,17,19,23H,2-6,12,15-16,18H2,1H3,(H,21,22)/b9-7-,11-8-,13-10-,17-14+/t19-/m0/s1</p>

</body>

</notes>

</species>

<species boundaryCondition="false" charge="0" constant="false" metaid="_metaM_o2_r" hasOnlySubstanceUnits="false" sboTerm="SBO:0000247" compartment="r" name="O2" id="M_o2_r" initialConcentration="1">

<notes>

<body xmlns="<http://www.w3.org/1999/xhtml>">

<p>FORMULA: O2</p>

<p>CHARGE: 0</p>

<p>HEPATONET_1.0_ABBREVIATION: HC00017</p>

<p>EHMN_ABBREVIATION: C00007</p>

<p>INCHI: InChI=1S/O2/c1-2</p>

</body>

</notes>

</species>

<species boundaryCondition="false" charge="-1" constant="false" metaid="_metaM_prostge2_r" hasOnlySubstanceUnits="false" sboTerm="SBO:0000247" compartment="r" name="prostaglandin E2(1-)" id="M_prostge2_r" initialConcentration="1">

<notes>

<body xmlns="<http://www.w3.org/1999/xhtml>">

<p>FORMULA: C20H31O5</p>

<p>CHARGE: -1</p>

<p>INCHI: InChI=1S/C20H32O5/c1-2-3-6-9-15(21)12-13-17-16(18(22)14-19(17)23)10-7-4-5-8-11-20(24)25/h4,7,12-13,15-17,19,21,23H,2-3,5-6,8-11,14H2,1H3,(H,24,25)/p-1/b7-4-,13-12+/t15-,16+,17+,19+/m0/s1</p>

<p>EHMN_ABBREVIATION: C00584</p>

</body>

</notes>

</species>

<species boundaryCondition="false" charge="-1" constant="false" metaid="_metaM_prostgh2_r" hasOnlySubstanceUnits="false" sboTerm="SBO:0000247" compartment="r" name="prostaglandin H2(1-)" id="M_prostgh2_r" initialConcentration="1">

<notes>

<body xmlns="<http://www.w3.org/1999/xhtml>">

<p>FORMULA: C20H31O5</p>

<p>CHARGE: -1</p>

<p>INCHI: InChI=1S/C20H32O5/c1-2-3-6-9-15(21)12-13-17-16(18-14-19(17)25-24-18)10-7-4-5-8-11-20(22)23/h4,7,12-13,15-19,21H,2-3,5-6,8-11,14H2,1H3,(H,22,23)/p-1/b7-4-,13-12+/t15-,16+,17+,18-,19+/m0/s1</p>

<p>EHMN_ABBREVIATION: C00427</p>

</body>

</notes>

</species>

<species boundaryCondition="false" charge="-1" constant="false" metaid="_metaM_arachd_r" hasOnlySubstanceUnits="false" sboTerm="SBO:0000247" compartment="r" name="arachidonate" id="M_arachd_r" initialConcentration="1">

<notes>

<body xmlns="<http://www.w3.org/1999/xhtml>">

<p>FORMULA: C20H31O2</p>

<p>CHARGE: -1</p>

<p>INCHI: InChI=1S/C20H32O2/c1-2-3-4-5-6-7-8-9-10-11-12-13-14-15-16-17-18-19-20(21)22/h6-7,9-10,12-13,15-16H,2-5,8,11,14,17-19H2,1H3,(H,21,22)/p-1/b7-6-,10-9-,13-12-,16-15-</p>

<p>HEPATONET_1.0_ABBREVIATION: HC00202</p>

<p>EHMN_ABBREVIATION: C00219</p>

</body>

</notes>

</species>

<species boundaryCondition="false" charge="0" constant="false" metaid="_metaM_h2o_r" hasOnlySubstanceUnits="false" sboTerm="SBO:0000247" compartment="r" name="H2O" id="M_h2o_r" initialConcentration="1">

<notes>

<body xmlns="<http://www.w3.org/1999/xhtml>">

<p>FORMULA: H2O</p>

<p>CHARGE: 0</p>

<p>HEPATONET_1.0_ABBREVIATION: HC00011</p>

<p>EHMN_ABBREVIATION: C00001</p>

<p>INCHI: InChI=1S/H2O/h1H2</p>

</body>

</notes>

</species>

<species boundaryCondition="false" charge="1" constant="false" metaid="_metaM_h_r" hasOnlySubstanceUnits="false" sboTerm="SBO:0000247" compartment="r" name="proton" id="M_h_r" initialConcentration="1">

<notes>

<body xmlns="<http://www.w3.org/1999/xhtml>">

<p>FORMULA: H</p>

<p>CHARGE: 1</p>

<p>HEPATONET_1.0_ABBREVIATION: HC00083</p>

<p>EHMN_ABBREVIATION: C00080</p>

<p>INCHI: InChI=1S/p+1/i/hH</p>

</body>

</notes>

</species>

<species boundaryCondition="false" charge="-4" constant="false" metaid="_metaM_nadph_r" hasOnlySubstanceUnits="false" sboTerm="SBO:0000247" compartment="r" name="Nicotinamide adenine dinucleotide phosphate - reduced" id="M_nadph_r" initialConcentration="1">

<notes>

<body xmlns="<http://www.w3.org/1999/xhtml>">

<p>FORMULA: C21H26N7O17P3</p>

<p>CHARGE: -4</p>

<p>HEPATONET_1.0_ABBREVIATION: HC00015</p>

<p>EHMN_ABBREVIATION: C00005</p>

<p>INCHI: InChI=1S/C21H30N7O17P3/c22-17-12-19(25-7-24-17)28(8-26-12)21-16(44-46(33,34)35)14(30)11(43-21)6-41-48(38,39)45-47(36,37)40-5-10-13(29)15(31)20(42-10)27-3-1-2-9(4-27)18(23)32/h1,3-4,7-8,10-11,13-16,20-21,29-31H,2,5-6H2,(H2,23,32)(H,36,37)(H,38,39)(H2,22,24,25)(H2,33,34,35)/p-4/t10-,11-,13-,14-,15-,16-,20-,21-/m1/s1</p>

</body>

</notes>

</species>

<species boundaryCondition="false" charge="-3" constant="false" metaid="_metaM_nadp_r" hasOnlySubstanceUnits="false" sboTerm="SBO:0000247" compartment="r" name="Nicotinamide adenine dinucleotide phosphate" id="M_nadp_r" initialConcentration="1">

<notes>

<body xmlns="<http://www.w3.org/1999/xhtml>">

<p>FORMULA: C21H25N7O17P3</p>

<p>CHARGE: -3</p>

<p>HEPATONET_1.0_ABBREVIATION: HC00016</p>

<p>EHMN_ABBREVIATION: C00006</p>

<p>INCHI: InChI=1S/C21H28N7O17P3/c22-17-12-19(25-7-24-17)28(8-26-12)21-16(44-46(33,34)35)14(30)11(43-21)6-41-48(38,39)45-47(36,37)40-5-10-13(29)15(31)20(42-10)27-3-1-2-9(4-27)18(23)32/h1-4,7-8,10-11,13-16,20-21,29-31H,5-6H2,(H7-,22,23,24,25,32,33,34,35,36,37,38,39)/p-3/t10-,11-,13-,14-,15-,16-,20-,21-/m1/s1</p>

</body>

</notes>

</species>

<species boundaryCondition="false" charge="-1" constant="false" metaid="_metaM_18harachd_r" hasOnlySubstanceUnits="false" sboTerm="SBO:0000247" compartment="r" name="18 hydroxy arachidonic acid" id="M_18harachd_r" initialConcentration="1">

<notes>

<body xmlns="<http://www.w3.org/1999/xhtml>">

<p>FORMULA: C20H31O3</p>

<p>CHARGE: -1</p>

<p>INCHI: InChI=1/C20H32O3/c1-2-19(21)17-15-13-11-9-7-5-3-4-6-8-10-12-14-16-18-20(22)23/h4-7,10-13,19,21H,2-3,8-9,14-18H2,1H3,(H,22,23)/b6-4-,7-5-,12-10-,13-11-/t19-/m1/s1</p>

</body>

</notes>

</species>

<species boundaryCondition="false" charge="-1" constant="false" metaid="_metaM_12harachd_r" hasOnlySubstanceUnits="false" sboTerm="SBO:0000247" compartment="r" name="12 hydroxy arachidonic acid" id="M_12harachd_r" initialConcentration="1">

<notes>

<body xmlns="<http://www.w3.org/1999/xhtml>">

<p>FORMULA: C20H31O3</p>

<p>CHARGE: -1</p>

<p>INCHI: InChI=1/C20H32O3/c1-2-3-4-5-10-13-16-19(21)17-14-11-8-6-7-9-12-15-18-20(22)23/h7-11,13-14,17,19,21H,2-6,12,15-16,18H2,1H3,(H,22,23)/b9-7+,11-8-,13-10-,17-14-</p>

</body>

</notes>

</species>

<species boundaryCondition="false" charge="-1" constant="false" metaid="_metaM_wharachd_r" hasOnlySubstanceUnits="false" sboTerm="SBO:0000247" compartment="r" name="w-hydroxyl arachidonic acid" id="M_wharachd_r" initialConcentration="1">

<notes>

<body xmlns="<http://www.w3.org/1999/xhtml>">

<p>FORMULA: C20H31O3</p>

<p>CHARGE: -1</p>

<p>INCHI: InChI=1/C20H32O3/c21-19-17-15-13-11-9-7-5-3-1-2-4-6-8-10-12-14-16-18-20(22)23/h4,6,8,10,12,14,16,18,21H,1-3,5,7,9,11,13,15,17,19H2,(H,22,23)/b6-4+,10-8+,14-12+,18-16+</p>

</body>

</notes>

</species>

<species boundaryCondition="false" charge="-4" constant="false" metaid="_metaM_atp_r" hasOnlySubstanceUnits="false" sboTerm="SBO:0000247" compartment="r" name="ATP(4-)" id="M_atp_r" initialConcentration="1">

<notes>

<body xmlns="<http://www.w3.org/1999/xhtml>">

<p>FORMULA: C10H12N5O13P3</p>

<p>CHARGE: -4</p>

<p>INCHI: InChI=1S/C10H16N5O13P3/c11-8-5-9(13-2-12-8)15(3-14-5)10-7(17)6(16)4(26-10)1-25-30(21,22)28-31(23,24)27-29(18,19)20/h2-4,6-7,10,16-17H,1H2,(H,21,22)(H,23,24)(H2,11,12,13)(H2,18,19,20)/p-3/t4-,6-,7-,10-/m1/s1</p>

<p>HEPATONET_1.0_ABBREVIATION: HC00012</p>

<p>EHMN_ABBREVIATION: C00002</p>

</body>

</notes>

</species>

<species boundaryCondition="false" charge="-4" constant="false" metaid="_metaM_arachdcoa_r" hasOnlySubstanceUnits="false" sboTerm="SBO:0000247" compartment="r" name="arachidonoyl-CoA(4-)" id="M_arachdcoa_r" initialConcentration="1">

<notes>

<body xmlns="<http://www.w3.org/1999/xhtml>">

<p>FORMULA: C41H62N7O17P3S</p>

<p>CHARGE: -4</p>

<p>INCHI: InChI=1S/C41H66N7O17P3S/c1-4-5-6-7-8-9-10-11-12-13-14-15-16-17-18-19-20-21-32(50)69-25-24-43-31(49)22-23-44-39(53)36(52)41(2,3)27-62-68(59,60)65-67(57,58)61-26-30-35(64-66(54,55)56)34(51)40(63-30)48-29-47-33-37(42)45-28-46-38(33)48/h8-9,11-12,14-15,17-18,28-30,34-36,40,51-52H,4-7,10,13,16,19-27H2,1-3H3,(H,43,49)(H,44,53)(H,57,58)(H,59,60)(H2,42,45,46)(H2,54,55,56)/p-4/b9-8-,12-11-,15-14-,18-17-/t30-,34-,35-,36+,40-/m1/s1</p>

<p>HEPATONET_1.0_ABBREVIATION: HC01986</p>

<p>EHMN_ABBREVIATION: C02249</p>

</body>

</notes>

</species>

<species boundaryCondition="false" charge="0" constant="false" metaid="_metaM_co2_r" hasOnlySubstanceUnits="false" sboTerm="SBO:0000247" compartment="r" name="CO2" id="M_co2_r" initialConcentration="1">

<notes>

<body xmlns="<http://www.w3.org/1999/xhtml>">

<p>FORMULA: CO2</p>

<p>CHARGE: 0</p>

<p>HEPATONET_1.0_ABBREVIATION: HC00021</p>

<p>EHMN_ABBREVIATION: C00011</p>

<p>INCHI: InChI=1S/CO2/c2-1-3</p>

</body>

</notes>

</species>

<species boundaryCondition="false" charge="-4" constant="false" metaid="_metaM_coa_r" hasOnlySubstanceUnits="false" sboTerm="SBO:0000247" compartment="r" name="Coenzyme A" id="M_coa_r" initialConcentration="1">

<notes>

<body xmlns="<http://www.w3.org/1999/xhtml>">

<p>FORMULA: C21H32N7O16P3S</p>

<p>CHARGE: -4</p>

<p>HEPATONET_1.0_ABBREVIATION: HC00020</p>

<p>EHMN_ABBREVIATION: C00010</p>

<p>INCHI: InChI=1S/C21H36N7O16P3S/c1-21(2,16(31)19(32)24-4-3-12(29)23-5-6-48)8-41-47(38,39)44-46(36,37)40-7-11-15(43-45(33,34)35)14(30)20(42-11)28-10-27-13-17(22)25-9-26-18(13)28/h9-11,14-16,20,30-31,48H,3-8H2,1-2H3,(H,23,29)(H,24,32)(H,36,37)(H,38,39)(H2,22,25,26)(H2,33,34,35)/p-4/t11-,14-,15-,16+,20-/m1/s1</p>

</body>

</notes>

</species>

<species boundaryCondition="false" charge="-1" constant="false" metaid="_metaM_C14771_r" hasOnlySubstanceUnits="false" sboTerm="SBO:0000247" compartment="r" name="14,15-EET" id="M_C14771_r" initialConcentration="1">

<notes>

<body xmlns="<http://www.w3.org/1999/xhtml>">

<p>FORMULA: C20H31O3</p>

<p>CHARGE: -1</p>

<p>INCHI: InChI=1/C20H32O3/c1-2-3-12-15-18-19(23-18)16-13-10-8-6-4-5-7-9-11-14-17-20(21)22/h4,6-7,9-10,13,18-19H,2-3,5,8,11-12,14-17H2,1H3,(H,21,22)/b6-4-,9-7-,13-10-</p>

<p>EHMN_ABBREVIATION: C14771</p>

</body>

</notes>

</species>

<species boundaryCondition="false" charge="-1" constant="false" metaid="_metaM_C14769_r" hasOnlySubstanceUnits="false" sboTerm="SBO:0000247" compartment="r" name="8,9-EET" id="M_C14769_r" initialConcentration="1">

<notes>

<body xmlns="<http://www.w3.org/1999/xhtml>">

<p>FORMULA: C20H31O3</p>

<p>CHARGE: -1</p>

<p>INCHI: InChI=1/C20H32O3/c1-2-3-4-5-6-7-8-9-12-15-18-19(23-18)16-13-10-11-14-17-20(21)22/h6-7,9-10,12-13,18-19H,2-5,8,11,14-17H2,1H3,(H,21,22)/b7-6-,12-9-,13-10-</p>

<p>EHMN_ABBREVIATION: C14769</p>

</body>

</notes>

</species>

<species boundaryCondition="false" charge="-1" constant="false" metaid="_metaM_C14770_r" hasOnlySubstanceUnits="false" sboTerm="SBO:0000247" compartment="r" name="11,12-EET" id="M_C14770_r" initialConcentration="1">

<notes>

<body xmlns="<http://www.w3.org/1999/xhtml>">

<p>FORMULA: C20H31O3</p>

<p>CHARGE: -1</p>

<p>INCHI: InChI=1/C20H32O3/c1-2-3-4-5-9-12-15-18-19(23-18)16-13-10-7-6-8-11-14-17-20(21)22/h6,8-10,12-13,18-19H,2-5,7,11,14-17H2,1H3,(H,21,22)/b8-6+,12-9+,13-10+</p>

<p>EHMN_ABBREVIATION: C14770</p>

</body>

</notes>

</species>

<species boundaryCondition="false" charge="-1" constant="false" metaid="_metaM_C14768_r" hasOnlySubstanceUnits="false" sboTerm="SBO:0000247" compartment="r" name="5,6-EET" id="M_C14768_r" initialConcentration="1">

<notes>

<body xmlns="<http://www.w3.org/1999/xhtml>">

<p>FORMULA: C20H31O3</p>

<p>CHARGE: -1</p>

<p>INCHI: InChI=1/C20H32O3/c1-2-3-4-5-6-7-8-9-10-11-12-13-15-18-19(23-18)16-14-17-20(21)22/h6-7,9-10,12-13,18-19H,2-5,8,11,14-17H2,1H3,(H,21,22)/b7-6-,10-9-,13-12-</p>

<p>EHMN_ABBREVIATION: C14768</p>

</body>

</notes>

</species>

<species boundaryCondition="false" charge="-1" constant="false" metaid="_metaM_arachd_e" hasOnlySubstanceUnits="false" sboTerm="SBO:0000247" compartment="e" name="arachidonate" id="M_arachd_e" initialConcentration="1">

<notes>

<body xmlns="<http://www.w3.org/1999/xhtml>">

<p>FORMULA: C20H31O2</p>

<p>CHARGE: -1</p>

<p>INCHI: InChI=1S/C20H32O2/c1-2-3-4-5-6-7-8-9-10-11-12-13-14-15-16-17-18-19-20(21)22/h6-7,9-10,12-13,15-16H,2-5,8,11,14,17-19H2,1H3,(H,21,22)/p-1/b7-6-,10-9-,13-12-,16-15-</p>

</body>

</notes>

</species>

<species boundaryCondition="false" charge="1" constant="false" metaid="_metaM_na1_e" hasOnlySubstanceUnits="false" sboTerm="SBO:0000247" compartment="e" name="Sodium" id="M_na1_e" initialConcentration="1">

<notes>

<body xmlns="<http://www.w3.org/1999/xhtml>">

<p>FORMULA: Na</p>

<p>CHARGE: 1</p>

<p>INCHI: </p>

</body>

</notes>

</species>

<species boundaryCondition="false" charge="1" constant="false" metaid="_metaM_na1_c" hasOnlySubstanceUnits="false" sboTerm="SBO:0000247" compartment="c" name="Sodium" id="M_na1_c" initialConcentration="1">

<notes>

<body xmlns="<http://www.w3.org/1999/xhtml>">

<p>FORMULA: Na</p>

<p>CHARGE: 1</p>

<p>INCHI: </p>

</body>

</notes>

</species>

<species boundaryCondition="false" charge="-1" constant="false" metaid="_metaM_C14771_c" hasOnlySubstanceUnits="false" sboTerm="SBO:0000247" compartment="c" name="14,15-EET" id="M_C14771_c" initialConcentration="1">

<notes>

<body xmlns="<http://www.w3.org/1999/xhtml>">

<p>FORMULA: C20H31O3</p>

<p>CHARGE: -1</p>

<p>INCHI: InChI=1/C20H32O3/c1-2-3-12-15-18-19(23-18)16-13-10-8-6-4-5-7-9-11-14-17-20(21)22/h4,6-7,9-10,13,18-19H,2-3,5,8,11-12,14-17H2,1H3,(H,21,22)/b6-4-,9-7-,13-10-</p>

<p>EHMN_ABBREVIATION: C14771</p>

</body>

</notes>

</species>

<species boundaryCondition="false" charge="-1" constant="false" metaid="_metaM_C14769_c" hasOnlySubstanceUnits="false" sboTerm="SBO:0000247" compartment="c" name="8,9-EET" id="M_C14769_c" initialConcentration="1">

<notes>

<body xmlns="<http://www.w3.org/1999/xhtml>">

<p>FORMULA: C20H31O3</p>

<p>CHARGE: -1</p>

<p>INCHI: InChI=1/C20H32O3/c1-2-3-4-5-6-7-8-9-12-15-18-19(23-18)16-13-10-11-14-17-20(21)22/h6-7,9-10,12-13,18-19H,2-5,8,11,14-17H2,1H3,(H,21,22)/b7-6-,12-9-,13-10-</p>

<p>EHMN_ABBREVIATION: C14769</p>

</body>

</notes>

</species>

<species boundaryCondition="false" charge="-1" constant="false" metaid="_metaM_C14770_c" hasOnlySubstanceUnits="false" sboTerm="SBO:0000247" compartment="c" name="11,12-EET" id="M_C14770_c" initialConcentration="1">

<notes>

<body xmlns="<http://www.w3.org/1999/xhtml>">

<p>FORMULA: C20H31O3</p>

<p>CHARGE: -1</p>

<p>INCHI: InChI=1/C20H32O3/c1-2-3-4-5-9-12-15-18-19(23-18)16-13-10-7-6-8-11-14-17-20(21)22/h6,8-10,12-13,18-19H,2-5,7,11,14-17H2,1H3,(H,21,22)/b8-6+,12-9+,13-10+</p>

<p>EHMN_ABBREVIATION: C14770</p>

</body>

</notes>

</species>

<species boundaryCondition="false" charge="-1" constant="false" metaid="_metaM_C14768_c" hasOnlySubstanceUnits="false" sboTerm="SBO:0000247" compartment="c" name="5,6-EET" id="M_C14768_c" initialConcentration="1">

<notes>

<body xmlns="<http://www.w3.org/1999/xhtml>">

<p>FORMULA: C20H31O3</p>

<p>CHARGE: -1</p>

<p>INCHI: InChI=1/C20H32O3/c1-2-3-4-5-6-7-8-9-10-11-12-13-15-18-19(23-18)16-14-17-20(21)22/h6-7,9-10,12-13,18-19H,2-5,8,11,14-17H2,1H3,(H,21,22)/b7-6-,10-9-,13-12-</p>

<p>EHMN_ABBREVIATION: C14768</p>

</body>

</notes>

</species>

<species boundaryCondition="false" charge="-1" constant="false" metaid="_metaM_hco3_e" hasOnlySubstanceUnits="false" sboTerm="SBO:0000247" compartment="e" name="Bicarbonate" id="M_hco3_e" initialConcentration="1">

<notes>

<body xmlns="<http://www.w3.org/1999/xhtml>">

<p>FORMULA: CHO3</p>

<p>CHARGE: -1</p>

<p>HEPATONET_1.0_ABBREVIATION: HC00253</p>

<p>EHMN_ABBREVIATION: C00288</p>

<p>INCHI: InChI=1S/CH2O3/c2-1(3)4/h(H2,2,3,4)/p-1</p>

</body>

</notes>

</species>

<species boundaryCondition="false" charge="-1" constant="false" metaid="_metaM_hco3_c" hasOnlySubstanceUnits="false" sboTerm="SBO:0000247" compartment="c" name="Bicarbonate" id="M_hco3_c" initialConcentration="1">

<notes>

<body xmlns="<http://www.w3.org/1999/xhtml>">

<p>FORMULA: CHO3</p>

<p>CHARGE: -1</p>

<p>HEPATONET_1.0_ABBREVIATION: HC00253</p>

<p>EHMN_ABBREVIATION: C00288</p>

<p>INCHI: InChI=1S/CH2O3/c2-1(3)4/h(H2,2,3,4)/p-1</p>

</body>

</notes>

</species>

<species boundaryCondition="false" charge="-3" constant="false" metaid="_metaM_ppi_c" hasOnlySubstanceUnits="false" sboTerm="SBO:0000247" compartment="c" name="Diphosphate" id="M_ppi_c" initialConcentration="1">

<notes>

<body xmlns="<http://www.w3.org/1999/xhtml>">

<p>FORMULA: HO7P2</p>

<p>CHARGE: -3</p>

<p>INCHI: </p>

</body>

</notes>

</species>

<species boundaryCondition="false" charge="1" constant="false" metaid="_metaM_nh4_r" hasOnlySubstanceUnits="false" sboTerm="SBO:0000247" compartment="r" name="ammonium" id="M_nh4_r" initialConcentration="1">

<notes>

<body xmlns="<http://www.w3.org/1999/xhtml>">

<p>FORMULA: H4N</p>

<p>CHARGE: 1</p>

<p>INCHI: InChI=1S/H3N/h1H3/p+1</p>

<p>HEPATONET_1.0_ABBREVIATION: HC00765</p>

<p>EHMN_ABBREVIATION: C01342</p>

</body>

</notes>

</species>

<species boundaryCondition="false" charge="-2" constant="false" metaid="_metaM_cbp_r" hasOnlySubstanceUnits="false" sboTerm="SBO:0000247" compartment="r" name="carbamoyl phosphate(2-)" id="M_cbp_r" initialConcentration="1">

<notes>

<body xmlns="<http://www.w3.org/1999/xhtml>">

<p>FORMULA: CH2NO5P</p>

<p>CHARGE: -2</p>

<p>INCHI: InChI=1S/CH4NO5P/c2-1(3)7-8(4,5)6/h(H2,2,3)(H2,4,5,6)/p-2</p>

<p>HEPATONET_1.0_ABBREVIATION: HC00164</p>

<p>EHMN_ABBREVIATION: C00169</p>

</body>

</notes>

</species>

<species boundaryCondition="false" charge="-4" constant="false" metaid="_metaM_atp_c" hasOnlySubstanceUnits="false" sboTerm="SBO:0000247" compartment="c" name="ATP(4-)" id="M_atp_c" initialConcentration="1">

<notes>

<body xmlns="<http://www.w3.org/1999/xhtml>">

<p>FORMULA: C10H12N5O13P3</p>

<p>CHARGE: -4</p>

<p>INCHI: InChI=1S/C10H16N5O13P3/c11-8-5-9(13-2-12-8)15(3-14-5)10-7(17)6(16)4(26-10)1-25-30(21,22)28-31(23,24)27-29(18,19)20/h2-4,6-7,10,16-17H,1H2,(H,21,22)(H,23,24)(H2,11,12,13)(H2,18,19,20)/p-4/t4-,6-,7-,10-/m1/s1</p>

</body>

</notes>

</species>

<species boundaryCondition="false" charge="-2" constant="false" metaid="_metaM_pi_c" hasOnlySubstanceUnits="false" sboTerm="SBO:0000247" compartment="c" name="hydrogenphosphate" id="M_pi_c" initialConcentration="1">

<notes>

<body xmlns="<http://www.w3.org/1999/xhtml>">

<p>FORMULA: HO4P</p>

<p>CHARGE: -2</p>

<p>INCHI: InChI=1S/H3O4P/c1-5(2,3)4/h(H3,1,2,3,4)/p-2</p>

</body>

</notes>

</species>

<species boundaryCondition="false" charge="-4" constant="false" metaid="_metaM_coa_c" hasOnlySubstanceUnits="false" sboTerm="SBO:0000247" compartment="c" name="Coenzyme A" id="M_coa_c" initialConcentration="1">

<notes>

<body xmlns="<http://www.w3.org/1999/xhtml>">

<p>FORMULA: C21H32N7O16P3S</p>

<p>CHARGE: -4</p>

<p>INCHI: </p>

</body>

</notes>

</species>

<species boundaryCondition="false" charge="-2" constant="false" metaid="_metaM_pi_r" hasOnlySubstanceUnits="false" sboTerm="SBO:0000247" compartment="r" name="hydrogenphosphate" id="M_pi_r" initialConcentration="1">

<notes>

<body xmlns="<http://www.w3.org/1999/xhtml>">

<p>FORMULA: HO4P</p>

<p>CHARGE: -2</p>

<p>HEPATONET_1.0_ABBREVIATION: HC00019</p>

<p>EHMN_ABBREVIATION: C00009</p>

<p>INCHI: InChI=1S/H3O4P/c1-5(2,3)4/h(H3,1,2,3,4)/p-2</p>

</body>

</notes>

</species>

<species boundaryCondition="false" charge="-1" constant="false" metaid="_metaM_orot_c" hasOnlySubstanceUnits="false" sboTerm="SBO:0000247" compartment="c" name="Orotate" id="M_orot_c" initialConcentration="1">

<notes>

<body xmlns="<http://www.w3.org/1999/xhtml>">

<p>FORMULA: C5H3N2O4</p>

<p>CHARGE: -1</p>

<p>HEPATONET_1.0_ABBREVIATION: HC00256</p>

<p>EHMN_ABBREVIATION: C00295</p>

<p>INCHI: InChI=1S/C5H4N2O4/c8-3-1-2(4(9)10)6-5(11)7-3/h1H,(H,9,10)(H2,6,7,8,11)/p-1</p>

</body>

</notes>

</species>

<species boundaryCondition="false" charge="-4" constant="false" metaid="_metaM_arachdcoa_c" hasOnlySubstanceUnits="false" sboTerm="SBO:0000247" compartment="c" name="arachidonoyl-CoA(4-)" id="M_arachdcoa_c" initialConcentration="1">

<notes>

<body xmlns="<http://www.w3.org/1999/xhtml>">

<p>FORMULA: C41H62N7O17P3S</p>

<p>CHARGE: -4</p>

<p>INCHI: InChI=1S/C41H66N7O17P3S/c1-4-5-6-7-8-9-10-11-12-13-14-15-16-17-18-19-20-21-32(50)69-25-24-43-31(49)22-23-44-39(53)36(52)41(2,3)27-62-68(59,60)65-67(57,58)61-26-30-35(64-66(54,55)56)34(51)40(63-30)48-29-47-33-37(42)45-28-46-38(33)48/h8-9,11-12,14-15,17-18,28-30,34-36,40,51-52H,4-7,10,13,16,19-27H2,1-3H3,(H,43,49)(H,44,53)(H,57,58)(H,59,60)(H2,42,45,46)(H2,54,55,56)/p-4/b9-8-,12-11-,15-14-,18-17-/t30-,34-,35-,36+,40-/m1/s1</p>

<p>HEPATONET_1.0_ABBREVIATION: HC01986</p>

<p>EHMN_ABBREVIATION: C02249</p>

</body>

</notes>

</species>

<species boundaryCondition="false" charge="0" constant="false" metaid="_metaM_xolest_hs_c" hasOnlySubstanceUnits="false" sboTerm="SBO:0000247" compartment="c" name="cholesterol ester" id="M_xolest_hs_c" initialConcentration="1">

<notes>

<body xmlns="<http://www.w3.org/1999/xhtml>">

<p>FORMULA: C27H45XCO2</p>

<p>CHARGE: 0</p>

<p>EHMN_ABBREVIATION: C02530</p>

<p>INCHI: </p>

</body>

</notes>

</species>

<species boundaryCondition="false" charge="-4" constant="false" metaid="_metaM_R2coa_hs_c" hasOnlySubstanceUnits="false" sboTerm="SBO:0000247" compartment="c" name="R group 2 Coenzyme A" id="M_R2coa_hs_c" initialConcentration="1">

<notes>

<body xmlns="<http://www.w3.org/1999/xhtml>">

<p>FORMULA: XCO2C21H31N7O15P3S</p>

<p>CHARGE: -4</p>

<p>INCHI: </p>

</body>

</notes>

</species>

<species boundaryCondition="false" charge="1" constant="false" metaid="_metaM_h_m" hasOnlySubstanceUnits="false" sboTerm="SBO:0000247" compartment="m" name="proton" id="M_h_m" initialConcentration="1">

<notes>

<body xmlns="<http://www.w3.org/1999/xhtml>">

<p>FORMULA: H</p>

<p>CHARGE: 1</p>

<p>HEPATONET_1.0_ABBREVIATION: HC00083</p>

<p>EHMN_ABBREVIATION: C00080</p>

<p>INCHI: InChI=1S/p+1/i/hH</p>

</body>

</notes>

</species>

<species boundaryCondition="false" charge="-4" constant="false" metaid="_metaM_nadph_m" hasOnlySubstanceUnits="false" sboTerm="SBO:0000247" compartment="m" name="Nicotinamide adenine dinucleotide phosphate - reduced" id="M_nadph_m" initialConcentration="1">

<notes>

<body xmlns="<http://www.w3.org/1999/xhtml>">

<p>FORMULA: C21H26N7O17P3</p>

<p>CHARGE: -4</p>

<p>HEPATONET_1.0_ABBREVIATION: HC00015</p>

<p>EHMN_ABBREVIATION: C00005</p>

<p>INCHI: InChI=1S/C21H30N7O17P3/c22-17-12-19(25-7-24-17)28(8-26-12)21-16(44-46(33,34)35)14(30)11(43-21)6-41-48(38,39)45-47(36,37)40-5-10-13(29)15(31)20(42-10)27-3-1-2-9(4-27)18(23)32/h1,3-4,7-8,10-11,13-16,20-21,29-31H,2,5-6H2,(H2,23,32)(H,36,37)(H,38,39)(H2,22,24,25)(H2,33,34,35)/p-4/t10-,11-,13-,14-,15-,16-,20-,21-/m1/s1</p>

</body>

</notes>

</species>

<species boundaryCondition="false" charge="-3" constant="false" metaid="_metaM_nadp_m" hasOnlySubstanceUnits="false" sboTerm="SBO:0000247" compartment="m" name="Nicotinamide adenine dinucleotide phosphate" id="M_nadp_m" initialConcentration="1">

<notes>

<body xmlns="<http://www.w3.org/1999/xhtml>">

<p>FORMULA: C21H25N7O17P3</p>

<p>CHARGE: -3</p>

<p>HEPATONET_1.0_ABBREVIATION: HC00016</p>

<p>EHMN_ABBREVIATION: C00006</p>

<p>INCHI: InChI=1S/C21H28N7O17P3/c22-17-12-19(25-7-24-17)28(8-26-12)21-16(44-46(33,34)35)14(30)11(43-21)6-41-48(38,39)45-47(36,37)40-5-10-13(29)15(31)20(42-10)27-3-1-2-9(4-27)18(23)32/h1-4,7-8,10-11,13-16,20-21,29-31H,5-6H2,(H7-,22,23,24,25,32,33,34,35,36,37,38,39)/p-3/t10-,11-,13-,14-,15-,16-,20-,21-/m1/s1</p>

</body>

</notes>

</species>

<species boundaryCondition="false" charge="-4" constant="false" metaid="_metaM_dmhepcoa_m" hasOnlySubstanceUnits="false" sboTerm="SBO:0000247" compartment="m" name="2,6-dimethyl heptanoylcoa" id="M_dmhepcoa_m" initialConcentration="1">

<notes>

<body xmlns="<http://www.w3.org/1999/xhtml>">

<p>FORMULA: C30H48N7O17P3S</p>

<p>CHARGE: -4</p>

</body>

</notes>

</species>

<species boundaryCondition="false" charge="-2" constant="false" metaid="_metaM_fad_m" hasOnlySubstanceUnits="false" sboTerm="SBO:0000247" compartment="m" name="Flavin adenine dinucleotide oxidized" id="M_fad_m" initialConcentration="1">

<notes>

<body xmlns="<http://www.w3.org/1999/xhtml>">

<p>FORMULA: C27H31N9O15P2</p>

<p>CHARGE: -2</p>

<p>HEPATONET_1.0_ABBREVIATION: HC00026</p>

<p>EHMN_ABBREVIATION: C00016</p>

<p>INCHI: InChI=1S/C27H33N9O15P2/c1-10-3-12-13(4-11(10)2)35(24-18(32-12)25(42)34-27(43)33-24)5-14(37)19(39)15(38)6-48-52(44,45)51-53(46,47)49-7-16-20(40)21(41)26(50-16)36-9-31-17-22(28)29-8-30-23(17)36/h3-4,8-9,14-16,19-21,26,37-41H,5-7H2,1-2H3,(H,44,45)(H,46,47)(H2,28,29,30)(H,34,42,43)/t14-,15+,16+,19-,20+,21+,26+/m0/s1</p>

</body>

</notes>

</species>

<species boundaryCondition="false" charge="0" constant="false" metaid="_metaM_h2o_m" hasOnlySubstanceUnits="false" sboTerm="SBO:0000247" compartment="m" name="H2O" id="M_h2o_m" initialConcentration="1">

<notes>

<body xmlns="<http://www.w3.org/1999/xhtml>">

<p>FORMULA: H2O</p>

<p>CHARGE: 0</p>

<p>HEPATONET_1.0_ABBREVIATION: HC00011</p>

<p>EHMN_ABBREVIATION: C00001</p>

<p>INCHI: InChI=1S/H2O/h1H2</p>

</body>

</notes>

</species>

<species boundaryCondition="false" charge="-4" constant="false" metaid="_metaM_coa_m" hasOnlySubstanceUnits="false" sboTerm="SBO:0000247" compartment="m" name="Coenzyme A" id="M_coa_m" initialConcentration="1">

<notes>

<body xmlns="<http://www.w3.org/1999/xhtml>">

<p>FORMULA: C21H32N7O16P3S</p>

<p>CHARGE: -4</p>

<p>HEPATONET_1.0_ABBREVIATION: HC00020</p>

<p>EHMN_ABBREVIATION: C00010</p>

<p>INCHI: InChI=1S/C21H36N7O16P3S/c1-21(2,16(31)19(32)24-4-3-12(29)23-5-6-48)8-41-47(38,39)44-46(36,37)40-7-11-15(43-45(33,34)35)14(30)20(42-11)28-10-27-13-17(22)25-9-26-18(13)28/h9-11,14-16,20,30-31,48H,3-8H2,1-2H3,(H,23,29)(H,24,32)(H,36,37)(H,38,39)(H2,22,25,26)(H2,33,34,35)/p-4/t11-,14-,15-,16+,20-/m1/s1</p>

</body>

</notes>

</species>

<species boundaryCondition="false" charge="-1" constant="false" metaid="_metaM_nad_m" hasOnlySubstanceUnits="false" sboTerm="SBO:0000247" compartment="m" name="Nicotinamide adenine dinucleotide" id="M_nad_m" initialConcentration="1">

<notes>

<body xmlns="<http://www.w3.org/1999/xhtml>">

<p>FORMULA: C21H26N7O14P2</p>

<p>CHARGE: -1</p>

<p>INCHI: InChI=1S/C21H27N7O14P2/c22-17-12-19(25-7-24-17)28(8-26-12)21-16(32)14(30)11(41-21)6-39-44(36,37)42-43(34,35)38-5-10-13(29)15(31)20(40-10)27-3-1-2-9(4-27)18(23)33/h1-4,7-8,10-11,13-16,20-21,29-32H,5-6H2,(H5-,22,23,24,25,33,34,35,36,37)/p-1/t10-,11-,13-,14-,15-,16-,20-,21-/m1/s1</p>

<p>HEPATONET_1.0_ABBREVIATION: HC00013</p>

<p>EHMN_ABBREVIATION: C00003</p>

</body>

</notes>

</species>

<species boundaryCondition="false" charge="-2" constant="false" metaid="_metaM_nadh_m" hasOnlySubstanceUnits="false" sboTerm="SBO:0000247" compartment="m" name="Nicotinamide adenine dinucleotide - reduced" id="M_nadh_m" initialConcentration="1">

<notes>

<body xmlns="<http://www.w3.org/1999/xhtml>">

<p>FORMULA: C21H27N7O14P2</p>

<p>CHARGE: -2</p>

<p>INCHI: InChI=1S/C21H29N7O14P2/c22-17-12-19(25-7-24-17)28(8-26-12)21-16(32)14(30)11(41-21)6-39-44(36,37)42-43(34,35)38-5-10-13(29)15(31)20(40-10)27-3-1-2-9(4-27)18(23)33/h1,3-4,7-8,10-11,13-16,20-21,29-32H,2,5-6H2,(H2,23,33)(H,34,35)(H,36,37)(H2,22,24,25)/p-2/t10-,11-,13-,14-,15-,16-,20-,21-/m1/s1</p>

<p>HEPATONET_1.0_ABBREVIATION: HC00014</p>

<p>EHMN_ABBREVIATION: C00004</p>

</body>

</notes>

</species>

<species boundaryCondition="false" charge="0" constant="false" metaid="_metaM_co2_c" hasOnlySubstanceUnits="false" sboTerm="SBO:0000247" compartment="c" name="CO2" id="M_co2_c" initialConcentration="1">

<notes>

<body xmlns="<http://www.w3.org/1999/xhtml>">

<p>FORMULA: CO2</p>

<p>CHARGE: 0</p>

<p>INCHI: InChI=1S/CO2/c2-1-3</p>

</body>

</notes>

</species>

<species boundaryCondition="false" charge="-4" constant="false" metaid="_metaM_nadph_c" hasOnlySubstanceUnits="false" sboTerm="SBO:0000247" compartment="c" name="Nicotinamide adenine dinucleotide phosphate - reduced" id="M_nadph_c" initialConcentration="1">

<notes>

<body xmlns="<http://www.w3.org/1999/xhtml>">

<p>FORMULA: C21H26N7O17P3</p>

<p>CHARGE: -4</p>

<p>HEPATONET_1.0_ABBREVIATION: HC00015</p>

<p>EHMN_ABBREVIATION: C00005</p>

<p>INCHI: InChI=1S/C21H30N7O17P3/c22-17-12-19(25-7-24-17)28(8-26-12)21-16(44-46(33,34)35)14(30)11(43-21)6-41-48(38,39)45-47(36,37)40-5-10-13(29)15(31)20(42-10)27-3-1-2-9(4-27)18(23)32/h1,3-4,7-8,10-11,13-16,20-21,29-31H,2,5-6H2,(H2,23,32)(H,36,37)(H,38,39)(H2,22,24,25)(H2,33,34,35)/p-4/t10-,11-,13-,14-,15-,16-,20-,21-/m1/s1</p>

</body>

</notes>

</species>

<species boundaryCondition="false" charge="-3" constant="false" metaid="_metaM_nadp_c" hasOnlySubstanceUnits="false" sboTerm="SBO:0000247" compartment="c" name="Nicotinamide adenine dinucleotide phosphate" id="M_nadp_c" initialConcentration="1">

<notes>

<body xmlns="<http://www.w3.org/1999/xhtml>">

<p>FORMULA: C21H25N7O17P3</p>

<p>CHARGE: -3</p>

<p>HEPATONET_1.0_ABBREVIATION: HC00016</p>

<p>EHMN_ABBREVIATION: C00006</p>

<p>INCHI: InChI=1S/C21H28N7O17P3/c22-17-12-19(25-7-24-17)28(8-26-12)21-16(44-46(33,34)35)14(30)11(43-21)6-41-48(38,39)45-47(36,37)40-5-10-13(29)15(31)20(42-10)27-3-1-2-9(4-27)18(23)32/h1-4,7-8,10-11,13-16,20-21,29-31H,5-6H2,(H7-,22,23,24,25,32,33,34,35,36,37,38,39)/p-3/t10-,11-,13-,14-,15-,16-,20-,21-/m1/s1</p>

</body>

</notes>

</species>

<species boundaryCondition="false" charge="0" constant="false" metaid="_metaM_co2_g" hasOnlySubstanceUnits="false" sboTerm="SBO:0000247" compartment="g" name="carbon dioxide" id="M_co2_g" initialConcentration="1">

<notes>

<body xmlns="<http://www.w3.org/1999/xhtml>">

<p>FORMULA: CO2</p>

<p>CHARGE: 0</p>

<p>INCHI: InChI=1S/CO2/c2-1-3</p>

<p>HEPATONET_1.0_ABBREVIATION: HC00021</p>

<p>EHMN_ABBREVIATION: C00011</p>

</body>

</notes>

</species>

<species boundaryCondition="false" charge="0" constant="false" metaid="_metaM_44mctr_c" hasOnlySubstanceUnits="false" sboTerm="SBO:0000247" compartment="c" name="4,4-dimethyl-5alpha-cholesta-8,14,24-trien-3beta-ol" id="M_44mctr_c" initialConcentration="1">

<notes>

<body xmlns="<http://www.w3.org/1999/xhtml>">

<p>FORMULA: C29H46O</p>

<p>CHARGE: 0</p>

<p>INCHI: InChI=1S/C29H46O/c1-19(2)9-8-10-20(3)22-12-13-23-21-11-14-25-27(4,5)26(30)16-18-29(25,7)24(21)15-17-28(22,23)6/h9,13,20,22,25-26,30H,8,10-12,14-18H2,1-7H3/t20-,22-,25+,26+,28-,29-/m1/s1</p>

<p>HEPATONET_1.0_ABBREVIATION: HC01808</p>

<p>EHMN_ABBREVIATION: C11455</p>

</body>

</notes>

</species>

<species boundaryCondition="false" charge="0" constant="false" metaid="_metaM_CE5049_c" hasOnlySubstanceUnits="false" sboTerm="SBO:0000247" compartment="c" name="one carbon unit" id="M_CE5049_c" initialConcentration="1">

<notes>

<body xmlns="<http://www.w3.org/1999/xhtml>">

<p>FORMULA: CH2</p>

<p>CHARGE: 0</p>

<p>EHMN_ABBREVIATION: CE5049</p>

</body>

</notes>

</species>

<species boundaryCondition="false" charge="-1" constant="false" metaid="_metaM_CE6000_c" hasOnlySubstanceUnits="false" sboTerm="SBO:0000247" compartment="c" name="nitrosoperoxycarbonate" id="M_CE6000_c" initialConcentration="1">

<notes>

<body xmlns="<http://www.w3.org/1999/xhtml>">

<p>FORMULA: CNO5</p>

<p>CHARGE: -1</p>

<p>INCHI: </p>

<p>EHMN_ABBREVIATION: CE6000</p>

</body>

</notes>

</species>

<species boundaryCondition="false" charge="0" constant="false" metaid="_metaM_HC02036_c" hasOnlySubstanceUnits="false" sboTerm="SBO:0000247" compartment="c" name="1-Acylglycerol-3P-arach" id="M_HC02036_c" initialConcentration="1">

<notes>

<body xmlns="<http://www.w3.org/1999/xhtml>">

<p>FORMULA: </p>

<p>CHARGE: 0</p>

</body>

</notes>

</species>

<species boundaryCondition="false" charge="-2" constant="false" metaid="_metaM_amp_c" hasOnlySubstanceUnits="false" sboTerm="SBO:0000247" compartment="c" name="AMP" id="M_amp_c" initialConcentration="1">

<notes>

<body xmlns="<http://www.w3.org/1999/xhtml>">

<p>FORMULA: C10H12N5O7P</p>

<p>CHARGE: -2</p>

<p>INCHI: </p>

</body>

</notes>

</species>

<species boundaryCondition="false" charge="1" constant="false" metaid="_metaM_nh4_x" hasOnlySubstanceUnits="false" sboTerm="SBO:0000247" compartment="x" name="Ammonium" id="M_nh4_x" initialConcentration="1">

<notes>

<body xmlns="<http://www.w3.org/1999/xhtml>">

<p>FORMULA: H4N</p>

<p>CHARGE: 1</p>

<p>HEPATONET_1.0_ABBREVIATION: HC00765</p>

<p>EHMN_ABBREVIATION: C01342</p>

<p>INCHI: InChI=1S/H3N/h1H3/p+1</p>

</body>

</notes>

</species>

<species boundaryCondition="false" charge="1" constant="false" metaid="_metaM_nh4_c" hasOnlySubstanceUnits="false" sboTerm="SBO:0000247" compartment="c" name="Ammonium" id="M_nh4_c" initialConcentration="1">

<notes>

<body xmlns="<http://www.w3.org/1999/xhtml>">

<p>FORMULA: H4N</p>

<p>CHARGE: 1</p>

<p>HEPATONET_1.0_ABBREVIATION: HC00765</p>

<p>EHMN_ABBREVIATION: C01342</p>

<p>INCHI: InChI=1S/H3N/h1H3/p+1</p>

</body>

</notes>

</species>

<species boundaryCondition="false" charge="1" constant="false" metaid="_metaM_nh4_n" hasOnlySubstanceUnits="false" sboTerm="SBO:0000247" compartment="n" name="ammonium" id="M_nh4_n" initialConcentration="1">

<notes>

<body xmlns="<http://www.w3.org/1999/xhtml>">

<p>FORMULA: H4N</p>

<p>CHARGE: 1</p>

<p>INCHI: InChI=1S/H3N/h1H3/p+1</p>

<p>HEPATONET_1.0_ABBREVIATION: HC00765</p>

<p>EHMN_ABBREVIATION: C01342</p>

</body>

</notes>

</species>

<species boundaryCondition="false" charge="-2" constant="false" metaid="_metaM_fprica_c" hasOnlySubstanceUnits="false" sboTerm="SBO:0000247" compartment="c" name="5-Formamido-1-(5-phospho-D-ribosyl)imidazole-4-carboxamide" id="M_fprica_c" initialConcentration="1">

<notes>

<body xmlns="<http://www.w3.org/1999/xhtml>">

<p>FORMULA: C10H13N4O9P</p>

<p>CHARGE: -2</p>

<p>HEPATONET_1.0_ABBREVIATION: HC01344</p>

<p>EHMN_ABBREVIATION: C04734</p>

<p>INCHI: InChI=1S/C10H15N4O9P/c11-8(18)5-9(13-3-15)14(2-12-5)10-7(17)6(16)4(23-10)1-22-24(19,20)21/h2-4,6-7,10,16-17H,1H2,(H2,11,18)(H,13,15)(H2,19,20,21)/p-2/t4-,6-,7-,10-/m1/s1</p>

</body>

</notes>

</species>

<species boundaryCondition="false" charge="-2" constant="false" metaid="_metaM_imp_c" hasOnlySubstanceUnits="false" sboTerm="SBO:0000247" compartment="c" name="IMP" id="M_imp_c" initialConcentration="1">

<notes>

<body xmlns="<http://www.w3.org/1999/xhtml>">

<p>FORMULA: C10H11N4O8P</p>

<p>CHARGE: -2</p>

<p>HEPATONET_1.0_ABBREVIATION: HC00128</p>

<p>EHMN_ABBREVIATION: C00130</p>

<p>INCHI: InChI=1S/C10H13N4O8P/c15-6-4(1-21-23(18,19)20)22-10(7(6)16)14-3-13-5-8(14)11-2-12-9(5)17/h2-4,6-7,10,15-16H,1H2,(H,11,12,17)(H2,18,19,20)/p-2/t4-,6-,7-,10-/m1/s1</p>

</body>

</notes>

</species>

<species boundaryCondition="false" charge="-3" constant="false" metaid="_metaM_gdp_g" hasOnlySubstanceUnits="false" sboTerm="SBO:0000247" compartment="g" name="GDP(3-)" id="M_gdp_g" initialConcentration="1">

<notes>

<body xmlns="<http://www.w3.org/1999/xhtml>">

<p>FORMULA: C10H12N5O11P2</p>

<p>CHARGE: -3</p>

<p>INCHI: InChI=1S/C10H15N5O11P2/c11-10-13-7-4(8(18)14-10)12-2-15(7)9-6(17)5(16)3(25-9)1-24-28(22,23)26-27(19,20)21/h2-3,5-6,9,16-17H,1H2,(H,22,23)(H2,19,20,21)(H3,11,13,14,18)/p-3/t3-,5-,6-,9-/m1/s1</p>

<p>HEPATONET_1.0_ABBREVIATION: HC00043</p>

<p>EHMN_ABBREVIATION: C00035</p>

</body>

</notes>

</species>

<species boundaryCondition="false" charge="-3" constant="false" metaid="_metaM_gdp_c" hasOnlySubstanceUnits="false" sboTerm="SBO:0000247" compartment="c" name="GDP" id="M_gdp_c" initialConcentration="1">

<notes>

<body xmlns="<http://www.w3.org/1999/xhtml>">

<p>FORMULA: C10H12N5O11P2</p>

<p>CHARGE: -3</p>

<p>HEPATONET_1.0_ABBREVIATION: HC00043</p>

<p>EHMN_ABBREVIATION: C00035</p>

<p>INCHI: InChI=1S/C10H15N5O11P2/c11-10-13-7-4(8(18)14-10)12-2-15(7)9-6(17)5(16)3(25-9)1-24-28(22,23)26-27(19,20)21/h2-3,5-6,9,16-17H,1H2,(H,22,23)(H2,19,20,21)(H3,11,13,14,18)/p-3/t3-,5-,6-,9-/m1/s1</p>

</body>

</notes>

</species>

<species boundaryCondition="false" charge="-3" constant="false" metaid="_metaM_adp_c" hasOnlySubstanceUnits="false" sboTerm="SBO:0000247" compartment="c" name="ADP" id="M_adp_c" initialConcentration="1">

<notes>

<body xmlns="<http://www.w3.org/1999/xhtml>">

<p>FORMULA: C10H12N5O10P2</p>

<p>CHARGE: -3</p>

<p>INCHI: InChI=1S/C10H15N5O10P2/c11-8-5-9(13-2-12-8)15(3-14-5)10-7(17)6(16)4(24-10)1-23-27(21,22)25-26(18,19)20/h2-4,6-7,10,16-17H,1H2,(H,21,22)(H2,11,12,13)(H2,18,19,20)/t4-,6-,7-,10-/m1/s1</p>

</body>

</notes>

</species>

<species boundaryCondition="false" charge="-1" constant="false" metaid="_metaM_CE5304_c" hasOnlySubstanceUnits="false" sboTerm="SBO:0000247" compartment="c" name="15-deoxy-PGD2" id="M_CE5304_c" initialConcentration="1">

<notes>

<body xmlns="<http://www.w3.org/1999/xhtml>">

<p>FORMULA: C20H29O4</p>

<p>CHARGE: -1</p>

<p>INCHI: InChI=1/C20H30O4/c1-2-3-4-5-6-9-12-16-17(19(22)15-18(16)21)13-10-7-8-11-14-20(23)24/h6-7,9-10,12,17,19,22H,2-5,8,11,13-15H2,1H3,(H,23,24)/p-1/b9-6-,10-7-,16-12+/t17-,19+/m0/s1</p>

<p>EHMN_ABBREVIATION: CE5304</p>

</body>

</notes>

</species>

<species boundaryCondition="false" charge="-1" constant="false" metaid="_metaM_prostgd2_c" hasOnlySubstanceUnits="false" sboTerm="SBO:0000247" compartment="c" name="prostaglandin D2(1-)" id="M_prostgd2_c" initialConcentration="1">

<notes>

<body xmlns="<http://www.w3.org/1999/xhtml>">

<p>FORMULA: C20H31O5</p>

<p>CHARGE: -1</p>

<p>INCHI: InChI=1S/C20H32O5/c1-2-3-6-9-15(21)12-13-17-16(18(22)14-19(17)23)10-7-4-5-8-11-20(24)25/h4,7,12-13,15-18,21-22H,2-3,5-6,8-11,14H2,1H3,(H,24,25)/p-1/b7-4-,13-12+/t15-,16+,17+,18-/m0/s1</p>

<p>EHMN_ABBREVIATION: C00696</p>

</body>

</notes>

</species>

<species boundaryCondition="false" charge="-1" constant="false" metaid="_metaM_prostgh2_c" hasOnlySubstanceUnits="false" sboTerm="SBO:0000247" compartment="c" name="prostaglandin H2(1-)" id="M_prostgh2_c" initialConcentration="1">

<notes>

<body xmlns="<http://www.w3.org/1999/xhtml>">

<p>FORMULA: C20H31O5</p>

<p>CHARGE: -1</p>

<p>INCHI: InChI=1S/C20H32O5/c1-2-3-6-9-15(21)12-13-17-16(18-14-19(17)25-24-18)10-7-4-5-8-11-20(22)23/h4,7,12-13,15-19,21H,2-3,5-6,8-11,14H2,1H3,(H,22,23)/p-1/b7-4-,13-12+/t15-,16+,17+,18-,19+/m0/s1</p>

<p>EHMN_ABBREVIATION: C00427</p>

</body>

</notes>

</species>

<species boundaryCondition="false" charge="-1" constant="false" metaid="_metaM_CE1243_c" hasOnlySubstanceUnits="false" sboTerm="SBO:0000247" compartment="c" name="12S-HHT" id="M_CE1243_c" initialConcentration="1">

<notes>

<body xmlns="<http://www.w3.org/1999/xhtml>">

<p>FORMULA: C17H27O3</p>

<p>CHARGE: -1</p>

<p>INCHI: InChI=1/C17H28O3/c1-2-3-10-13-16(18)14-11-8-6-4-5-7-9-12-15-17(19)20/h5-8,11,14,16,18H,2-4,9-10,12-13,15H2,1H3,(H,19,20)/p-1/b7-5-,8-6+,14-11+/t16-/m0/s1</p>

<p>EHMN_ABBREVIATION: CE1243</p>

</body>

</notes>

</species>

<species boundaryCondition="false" charge="-1" constant="false" metaid="_metaM_CE0737_c" hasOnlySubstanceUnits="false" sboTerm="SBO:0000247" compartment="c" name="malonic dialdehyde" id="M_CE0737_c" initialConcentration="1">

<notes>

<body xmlns="<http://www.w3.org/1999/xhtml>">

<p>FORMULA: C3H3O2</p>

<p>CHARGE: -1</p>

<p>INCHI: InChI=1/C3H4O2/c4-2-1-3-5/h2-3H,1H2</p>

<p>EHMN_ABBREVIATION: CE0737</p>

</body>

</notes>

</species>

<species boundaryCondition="false" charge="0" constant="false" metaid="_metaM_crn_c" hasOnlySubstanceUnits="false" sboTerm="SBO:0000247" compartment="c" name="L-Carnitine" id="M_crn_c" initialConcentration="1">

<notes>

<body xmlns="<http://www.w3.org/1999/xhtml>">

<p>FORMULA: C7H15NO3</p>

<p>CHARGE: 0</p>

<p>HEPATONET_1.0_ABBREVIATION: HC00270</p>

<p>EHMN_ABBREVIATION: C00318</p>

<p>INCHI: InChI=1S/C7H15NO3/c1-8(2,3)5-6(9)4-7(10)11/h6,9H,4-5H2,1-3H3/t6-/m1/s1</p>

</body>

</notes>

</species>

<species boundaryCondition="false" charge="0" constant="false" metaid="_metaM_HC10854_c" hasOnlySubstanceUnits="false" sboTerm="SBO:0000247" compartment="c" name="palmitoleoyl-Carnitine" id="M_HC10854_c" initialConcentration="1">

<notes>

<body xmlns="<http://www.w3.org/1999/xhtml>">

<p>FORMULA: </p>

<p>CHARGE: 0</p>

<p>INCHI: </p>

</body>

</notes>

</species>

<species boundaryCondition="false" charge="0" constant="false" metaid="_metaM_HC10855_c" hasOnlySubstanceUnits="false" sboTerm="SBO:0000247" compartment="c" name="linoleic-Carnitine" id="M_HC10855_c" initialConcentration="1">

<notes>

<body xmlns="<http://www.w3.org/1999/xhtml>">

<p>FORMULA: </p>

<p>CHARGE: 0</p>

</body>

</notes>

</species>

<species boundaryCondition="false" charge="-2" constant="false" metaid="_metaM_fadh2_c" hasOnlySubstanceUnits="false" sboTerm="SBO:0000247" compartment="c" name="FADH2(2-)" id="M_fadh2_c" initialConcentration="1">

<notes>

<body xmlns="<http://www.w3.org/1999/xhtml>">

<p>FORMULA: C27H33N9O15P2</p>

<p>CHARGE: -2</p>

<p>INCHI: InChI=1S/C27H35N9O15P2/c1-10-3-12-13(4-11(10)2)35(24-18(32-12)25(42)34-27(43)33-24)5-14(37)19(39)15(38)6-48-52(44,45)51-53(46,47)49-7-16-20(40)21(41)26(50-16)36-9-31-17-22(28)29-8-30-23(17)36/h3-4,8-9,14-16,19-21,26,32,37-41H,5-7H2,1-2H3,(H,44,45)(H,46,47)(H2,28,29,30)(H2,33,34,42,43)/p-2/t14-,15+,16+,19-,20+,21+,26+/m0/s1</p>

<p>HEPATONET_1.0_ABBREVIATION: HC00770</p>

<p>EHMN_ABBREVIATION: C01352</p>

</body>

</notes>

</species>

<species boundaryCondition="false" charge="-2" constant="false" metaid="_metaM_fad_c" hasOnlySubstanceUnits="false" sboTerm="SBO:0000247" compartment="c" name="Flavin adenine dinucleotide oxidized" id="M_fad_c" initialConcentration="1">

<notes>

<body xmlns="<http://www.w3.org/1999/xhtml>">

<p>FORMULA: C27H31N9O15P2</p>

<p>CHARGE: -2</p>

<p>HEPATONET_1.0_ABBREVIATION: HC00026</p>

<p>EHMN_ABBREVIATION: C00016</p>

<p>INCHI: InChI=1S/C27H33N9O15P2/c1-10-3-12-13(4-11(10)2)35(24-18(32-12)25(42)34-27(43)33-24)5-14(37)19(39)15(38)6-48-52(44,45)51-53(46,47)49-7-16-20(40)21(41)26(50-16)36-9-31-17-22(28)29-8-30-23(17)36/h3-4,8-9,14-16,19-21,26,37-41H,5-7H2,1-2H3,(H,44,45)(H,46,47)(H2,28,29,30)(H,34,42,43)/t14-,15+,16+,19-,20+,21+,26+/m0/s1</p>

</body>

</notes>

</species>

<species boundaryCondition="false" charge="0" constant="false" metaid="_metaM_HC02114_c" hasOnlySubstanceUnits="false" sboTerm="SBO:0000247" compartment="c" name="FADH-redox-potential" id="M_HC02114_c" initialConcentration="1">

<notes>

<body xmlns="<http://www.w3.org/1999/xhtml>">

<p>FORMULA: </p>

<p>CHARGE: 0</p>

</body>

</notes>

</species>

<species boundaryCondition="false" charge="-1" constant="false" metaid="_metaM_nad_c" hasOnlySubstanceUnits="false" sboTerm="SBO:0000247" compartment="c" name="Nicotinamide adenine dinucleotide" id="M_nad_c" initialConcentration="1">

<notes>

<body xmlns="<http://www.w3.org/1999/xhtml>">

<p>FORMULA: C21H26N7O14P2</p>

<p>CHARGE: -1</p>

<p>INCHI: InChI=1S/C21H27N7O14P2/c22-17-12-19(25-7-24-17)28(8-26-12)21-16(32)14(30)11(41-21)6-39-44(36,37)42-43(34,35)38-5-10-13(29)15(31)20(40-10)27-3-1-2-9(4-27)18(23)33/h1-4,7-8,10-11,13-16,20-21,29-32H,5-6H2,(H5-,22,23,24,25,33,34,35,36,37)/p-1/t10-,11-,13-,14-,15-,16-,20-,21-/m1/s1</p>

<p>HEPATONET_1.0_ABBREVIATION: HC00013</p>

<p>EHMN_ABBREVIATION: C00003</p>

</body>

</notes>

</species>

<species boundaryCondition="false" charge="-2" constant="false" metaid="_metaM_nadh_c" hasOnlySubstanceUnits="false" sboTerm="SBO:0000247" compartment="c" name="Nicotinamide adenine dinucleotide - reduced" id="M_nadh_c" initialConcentration="1">

<notes>

<body xmlns="<http://www.w3.org/1999/xhtml>">

<p>FORMULA: C21H27N7O14P2</p>

<p>CHARGE: -2</p>

<p>INCHI: InChI=1S/C21H29N7O14P2/c22-17-12-19(25-7-24-17)28(8-26-12)21-16(32)14(30)11(41-21)6-39-44(36,37)42-43(34,35)38-5-10-13(29)15(31)20(40-10)27-3-1-2-9(4-27)18(23)33/h1,3-4,7-8,10-11,13-16,20-21,29-32H,2,5-6H2,(H2,23,33)(H,34,35)(H,36,37)(H2,22,24,25)/p-2/t10-,11-,13-,14-,15-,16-,20-,21-/m1/s1</p>

<p>HEPATONET_1.0_ABBREVIATION: HC00014</p>

<p>EHMN_ABBREVIATION: C00004</p>

</body>

</notes>

</species>

<species boundaryCondition="false" charge="-1" constant="false" metaid="_metaM_CE5534_c" hasOnlySubstanceUnits="false" sboTerm="SBO:0000247" compartment="c" name="9,11-cycloperoxy-5-hydroperoxy-6E,14Z-eicosadienoate" id="M_CE5534_c" initialConcentration="1">

<notes>

<body xmlns="<http://www.w3.org/1999/xhtml>">

<p>FORMULA: C20H31O6</p>

<p>CHARGE: -1</p>

<p>INCHI: </p>

<p>EHMN_ABBREVIATION: CE5534</p>

</body>

</notes>

</species>

<species boundaryCondition="false" charge="-1" constant="false" metaid="_metaM_CE5828_c" hasOnlySubstanceUnits="false" sboTerm="SBO:0000247" compartment="c" name="5,9,11-trihydroxyprosta-6E,14Z-dien-1-oate" id="M_CE5828_c" initialConcentration="1">

<notes>

<body xmlns="<http://www.w3.org/1999/xhtml>">

<p>FORMULA: C20H33O5</p>

<p>CHARGE: -1</p>

<p>INCHI: </p>

<p>EHMN_ABBREVIATION: CE5828</p>

</body>

</notes>

</species>

<species boundaryCondition="false" charge="-1" constant="false" metaid="_metaM_CE5535_c" hasOnlySubstanceUnits="false" sboTerm="SBO:0000247" compartment="c" name="9-peroxy-5Z,7E,11Z,14Z-eicosatetraenoate" id="M_CE5535_c" initialConcentration="1">

<notes>

<body xmlns="<http://www.w3.org/1999/xhtml>">

<p>FORMULA: C20H30O4</p>

<p>CHARGE: -1</p>

<p>INCHI: InChI=1/C20H31O4/c1-2-3-4-5-6-7-8-10-13-16-19(24-23)17-14-11-9-12-15-18-20(21)22/h6-7,9-11,13-14,17,19H,2-5,8,12,15-16,18H2,1H3,(H,21,22)/p-1/b7-6-,11-9-,13-10-,17-14+</p>

<p>EHMN_ABBREVIATION: CE5535</p>

</body>

</notes>

</species>

<species boundaryCondition="false" charge="-1" constant="false" metaid="_metaM_CE5928_c" hasOnlySubstanceUnits="false" sboTerm="SBO:0000247" compartment="c" name="8-peroxy-5Z,9E,11Z,14Z-eicosatetraenoate" id="M_CE5928_c" initialConcentration="1">

<notes>

<body xmlns="<http://www.w3.org/1999/xhtml>">

<p>FORMULA: C20H30O4</p>

<p>CHARGE: -1</p>

<p>INCHI: InChI=1/C20H31O4/c1-2-3-4-5-6-7-8-9-10-13-16-19(24-23)17-14-11-12-15-18-20(21)22/h6-7,9-11,13-14,16,19H,2-5,8,12,15,17-18H2,1H3,(H,21,22)/p-1/b7-6-,10-9-,14-11-,16-13+</p>

<p>EHMN_ABBREVIATION: CE5928</p>

</body>

</notes>

</species>

<species boundaryCondition="false" charge="-1" constant="false" metaid="_metaM_CE5930_c" hasOnlySubstanceUnits="false" sboTerm="SBO:0000247" compartment="c" name="5,9-cyclo-6,8-cycloperoxy-12-hydroperoxy-(10E,14Z)-eicosadienoic acid" id="M_CE5930_c" initialConcentration="1">

<notes>

<body xmlns="<http://www.w3.org/1999/xhtml>">

<p>FORMULA: C20H31O6</p>

<p>CHARGE: -1</p>

<p>INCHI: </p>

<p>EHMN_ABBREVIATION: CE5930</p>

</body>

</notes>

</species>

<species boundaryCondition="false" charge="-1" constant="false" metaid="_metaM_CE5931_c" hasOnlySubstanceUnits="false" sboTerm="SBO:0000247" compartment="c" name="5,9-cyclo-6,8,12-trihydroxy-(10E,14Z)-eicosadienoic acid" id="M_CE5931_c" initialConcentration="1">

<notes>

<body xmlns="<http://www.w3.org/1999/xhtml>">

<p>FORMULA: C20H33O5</p>

<p>CHARGE: -1</p>

<p>INCHI: </p>

<p>EHMN_ABBREVIATION: CE5931</p>

</body>

</notes>

</species>

<species boundaryCondition="false" charge="-1" constant="false" metaid="_metaM_CE5929_c" hasOnlySubstanceUnits="false" sboTerm="SBO:0000247" compartment="c" name="11,15-cyclo-8,12,14-trihydroxy-(5Z,9E)-eicosadienoic acid" id="M_CE5929_c" initialConcentration="1">

<notes>

<body xmlns="<http://www.w3.org/1999/xhtml>">

<p>FORMULA: C20H33O5</p>

<p>CHARGE: -1</p>

<p>INCHI: </p>

<p>EHMN_ABBREVIATION: CE5929</p>

</body>

</notes>

</species>

<species boundaryCondition="false" charge="-1" constant="false" metaid="_metaM_CE5925_c" hasOnlySubstanceUnits="false" sboTerm="SBO:0000247" compartment="c" name="12-peroxy-5Z,8Z,10E,14Z-eicosatetraenoate" id="M_CE5925_c" initialConcentration="1">

<notes>

<body xmlns="<http://www.w3.org/1999/xhtml>">

<p>FORMULA: C20H30O4</p>

<p>CHARGE: -1</p>

<p>INCHI: InChI=1/C20H31O4/c1-2-3-4-5-10-13-16-19(24-23)17-14-11-8-6-7-9-12-15-18-20(21)22/h7-11,13-14,17,19H,2-6,12,15-16,18H2,1H3,(H,21,22)/p-1/b9-7-,11-8-,13-10-,17-14+</p>

<p>EHMN_ABBREVIATION: CE5925</p>

</body>

</notes>

</species>

<species boundaryCondition="false" charge="-1" constant="false" metaid="_metaM_CE5926_c" hasOnlySubstanceUnits="false" sboTerm="SBO:0000247" compartment="c" name="11,15-cyclo-12,14-cycloperoxy-8-hydroperoxy-5Z,9E-eicosadienoic" id="M_CE5926_c" initialConcentration="1">

<notes>

<body xmlns="<http://www.w3.org/1999/xhtml>">

<p>FORMULA: C20H31O6</p>

<p>CHARGE: -1</p>

<p>INCHI: </p>

<p>EHMN_ABBREVIATION: CE5926</p>

</body>

</notes>

</species>

<species boundaryCondition="false" charge="-1" constant="false" metaid="_metaM_CE5537_c" hasOnlySubstanceUnits="false" sboTerm="SBO:0000247" compartment="c" name="11-peroxy-5Z,8Z,12E,14Z-eicosatetraenoate" id="M_CE5537_c" initialConcentration="1">

<notes>

<body xmlns="<http://www.w3.org/1999/xhtml>">

<p>FORMULA: C20H30O4</p>

<p>CHARGE: -1</p>

<p>INCHI: InChI=1/C20H31O4/c1-2-3-4-5-7-10-13-16-19(24-23)17-14-11-8-6-9-12-15-18-20(21)22/h6-7,9-11,13-14,16,19H,2-5,8,12,15,17-18H2,1H3,(H,21,22)/p-1/b9-6-,10-7-,14-11-,16-13+</p>

<p>EHMN_ABBREVIATION: CE5537</p>

</body>

</notes>

</species>

<species boundaryCondition="false" charge="-1" constant="false" metaid="_metaM_CE5533_c" hasOnlySubstanceUnits="false" sboTerm="SBO:0000247" compartment="c" name="9,11-cycloperoxy-15-hydroperoxy-5Z,13E-eicosadienoate" id="M_CE5533_c" initialConcentration="1">

<notes>

<body xmlns="<http://www.w3.org/1999/xhtml>">

<p>FORMULA: C20H31O6</p>

<p>CHARGE: -1</p>

<p>INCHI: </p>

<p>EHMN_ABBREVIATION: CE5533</p>

</body>

</notes>

</species>

<species boundaryCondition="false" charge="-1" constant="false" metaid="_metaM_CE5924_c" hasOnlySubstanceUnits="false" sboTerm="SBO:0000247" compartment="c" name="9,11,15-trihydroxyprosta-5Z,13E-dien-1-oate" id="M_CE5924_c" initialConcentration="1">

<notes>

<body xmlns="<http://www.w3.org/1999/xhtml>">

<p>FORMULA: C20H33O5</p>

<p>CHARGE: -1</p>

<p>INCHI: </p>

<p>EHMN_ABBREVIATION: CE5924</p>

</body>

</notes>

</species>

<species boundaryCondition="false" charge="0" constant="false" metaid="_metaM_HC02199_c" hasOnlySubstanceUnits="false" sboTerm="SBO:0000247" compartment="c" name="glutathionyl-leuc4" id="M_HC02199_c" initialConcentration="1">

<notes>

<body xmlns="<http://www.w3.org/1999/xhtml>">

<p>FORMULA: </p>

<p>CHARGE: 0</p>

<p>INCHI: </p>

</body>

</notes>

</species>

<species boundaryCondition="false" charge="0" constant="false" metaid="_metaM_HC02199_e" hasOnlySubstanceUnits="false" sboTerm="SBO:0000247" compartment="e" name="glutathionyl-leuc4" id="M_HC02199_e" initialConcentration="1">

<notes>

<body xmlns="<http://www.w3.org/1999/xhtml>">

<p>FORMULA: </p>

<p>CHARGE: 0</p>

<p>INCHI: </p>

</body>

</notes>

</species>

<species boundaryCondition="false" charge="-1" constant="false" metaid="_metaM_HC02192_c" hasOnlySubstanceUnits="false" sboTerm="SBO:0000247" compartment="c" name="taurolithocholate" id="M_HC02192_c" initialConcentration="1">

<notes>

<body xmlns="<http://www.w3.org/1999/xhtml>">

<p>FORMULA: C26H44NO5S</p>

<p>CHARGE: -1</p>

<p>INCHI: InChI=1S/C26H45NO5S/c1-17(4-9-24(29)27-14-15-33(30,31)32)21-7-8-22-20-6-5-18-16-19(28)10-12-25(18,2)23(20)11-13-26(21,22)3/h17-23,28H,4-16H2,1-3H3,(H,27,29)(H,30,31,32)/p-1/t17-,18-,19-,20+,21-,22+,23+,25+,26-/m1/s1</p>

<p>EHMN_ABBREVIATION: C02592</p>

</body>

</notes>

</species>

<species boundaryCondition="false" charge="-1" constant="false" metaid="_metaM_HC02193_c" hasOnlySubstanceUnits="false" sboTerm="SBO:0000247" compartment="c" name="glycolithocholate" id="M_HC02193_c" initialConcentration="1">

<notes>

<body xmlns="<http://www.w3.org/1999/xhtml>">

<p>FORMULA: C26H42NO4</p>

<p>CHARGE: -1</p>

<p>INCHI: InChI=1S/C26H43NO4/c1-16(4-9-23(29)27-15-24(30)31)20-7-8-21-19-6-5-17-14-18(28)10-12-25(17,2)22(19)11-13-26(20,21)3/h16-22,28H,4-15H2,1-3H3,(H,27,29)(H,30,31)/p-1/t16-,17-,18-,19+,20-,21+,22+,25+,26-/m1/s1</p>

</body>

</notes>

</species>

<species boundaryCondition="false" charge="0" constant="false" metaid="_metaM_HC02194_c" hasOnlySubstanceUnits="false" sboTerm="SBO:0000247" compartment="c" name="ursodeoxycholate" id="M_HC02194_c" initialConcentration="1">

<notes>

<body xmlns="<http://www.w3.org/1999/xhtml>">

<p>FORMULA: </p>

<p>CHARGE: 0</p>

<p>INCHI: </p>

</body>

</notes>

</species>

<species boundaryCondition="false" charge="-2" constant="false" metaid="_metaM_HC02197_c" hasOnlySubstanceUnits="false" sboTerm="SBO:0000247" compartment="c" name="sulfoglycolithocholate(2-)" id="M_HC02197_c" initialConcentration="1">

<notes>

<body xmlns="<http://www.w3.org/1999/xhtml>">

<p>FORMULA: C26H41NO7S</p>

<p>CHARGE: -2</p>

<p>INCHI: InChI=1S/C26H43NO7S/c1-16(4-9-23(28)27-15-24(29)30)20-7-8-21-19-6-5-17-14-18(34-35(31,32)33)10-12-25(17,2)22(19)11-13-26(20,21)3/h16-22H,4-15H2,1-3H3,(H,27,28)(H,29,30)(H,31,32,33)/p-2/t16-,17-,18-,19+,20-,21+,22+,25+,26-/m1/s1</p>

</body>

</notes>

</species>

<species boundaryCondition="false" charge="0" constant="false" metaid="_metaM_HC02196_c" hasOnlySubstanceUnits="false" sboTerm="SBO:0000247" compartment="c" name="glycoursodeoxycholate" id="M_HC02196_c" initialConcentration="1">

<notes>

<body xmlns="<http://www.w3.org/1999/xhtml>">

<p>FORMULA: </p>

<p>CHARGE: 0</p>

<p>INCHI: </p>

</body>

</notes>

</species>

<species boundaryCondition="false" charge="0" constant="false" metaid="_metaM_HC02195_c" hasOnlySubstanceUnits="false" sboTerm="SBO:0000247" compartment="c" name="tauroursodeoxycholate" id="M_HC02195_c" initialConcentration="1">

<notes>

<body xmlns="<http://www.w3.org/1999/xhtml>">

<p>FORMULA: </p>

<p>CHARGE: 0</p>

<p>INCHI: </p>

</body>

</notes>

</species>

<species boundaryCondition="false" charge="0" constant="false" metaid="_metaM_HC02220_c" hasOnlySubstanceUnits="false" sboTerm="SBO:0000247" compartment="c" name="sulfochenodeoxycholate" id="M_HC02220_c" initialConcentration="1">

<notes>

<body xmlns="<http://www.w3.org/1999/xhtml>">

<p>FORMULA: </p>

<p>CHARGE: 0</p>

<p>INCHI: </p>

</body>

</notes>

</species>

<species boundaryCondition="false" charge="0" constant="false" metaid="_metaM_HC02187_c" hasOnlySubstanceUnits="false" sboTerm="SBO:0000247" compartment="c" name="reverse-triiodthyronine" id="M_HC02187_c" initialConcentration="1">

<notes>

<body xmlns="<http://www.w3.org/1999/xhtml>">

<p>FORMULA: C15H12I3NO4</p>

<p>CHARGE: 0</p>

<p>INCHI: InChI=1S/C15H12I3NO4/c16-9-3-7(4-12(19)15(21)22)1-2-13(9)23-8-5-10(17)14(20)11(18)6-8/h1-3,5-6,12,20H,4,19H2,(H,21,22)</p>

</body>

</notes>

</species>

<species boundaryCondition="false" charge="-1" constant="false" metaid="_metaM_HC02180_c" hasOnlySubstanceUnits="false" sboTerm="SBO:0000247" compartment="c" name="thromboxane-b2" id="M_HC02180_c" initialConcentration="1">

<notes>

<body xmlns="<http://www.w3.org/1999/xhtml>">

<p>FORMULA: C20H33O6</p>

<p>CHARGE: -1</p>

<p>INCHI: InChI=1S/C20H34O6/c1-2-3-6-9-15(21)12-13-18-16(17(22)14-20(25)26-18)10-7-4-5-8-11-19(23)24/h4,7,12-13,15-18,20-22,25H,2-3,5-6,8-11,14H2,1H3,(H,23,24)/b7-4-,13-12+/t15-,16-,17-,18+,20?/m0/s1</p>

<p>EHMN_ABBREVIATION: C05963</p>

</body>

</notes>

</species>

<species boundaryCondition="false" charge="-1" constant="false" metaid="_metaM_HC02179_c" hasOnlySubstanceUnits="false" sboTerm="SBO:0000247" compartment="c" name="20-hydroxy-arachidonate" id="M_HC02179_c" initialConcentration="1">

<notes>

<body xmlns="<http://www.w3.org/1999/xhtml>">

<p>FORMULA: C20H31O3</p>

<p>CHARGE: -1</p>

<p>INCHI: InChI=1S/C20H32O3/c21-19-17-15-13-11-9-7-5-3-1-2-4-6-8-10-12-14-16-18-20(22)23/h1,3-4,6-7,9-10,12,21H,2,5,8,11,13-19H2,(H,22,23)/b3-1-,6-4-,9-7-,12-10-</p>

<p>EHMN_ABBREVIATION: C14748</p>

</body>

</notes>

</species>

<species boundaryCondition="false" charge="-2" constant="false" metaid="_metaM_leuktrF4_c" hasOnlySubstanceUnits="false" sboTerm="SBO:0000247" compartment="c" name="leukotriene F4" id="M_leuktrF4_c" initialConcentration="1">

<notes>

<body xmlns="<http://www.w3.org/1999/xhtml>">

<p>FORMULA: C28H42N2O8S</p>

<p>CHARGE: -2</p>

<p>INCHI: InChI=1S/C28H44N2O8S/c1-2-3-4-5-6-7-8-9-10-11-12-13-16-24(23(31)15-14-17-26(33)34)39-20-22(28(37)38)30-25(32)19-18-21(29)27(35)36/h6-7,9-13,16,21-24,31H,2-5,8,14-15,17-20,29H2,1H3,(H,30,32)(H,33,34)(H,35,36)(H,37,38)/b7-6-,10-9-,12-11+,16-13+/t21-,22+,23+,24-/m1/s1</p>

</body>

</notes>

</species>

<species boundaryCondition="false" charge="-1" constant="false" metaid="_metaM_HC02205_c" hasOnlySubstanceUnits="false" sboTerm="SBO:0000247" compartment="c" name="prostaglandin-b2" id="M_HC02205_c" initialConcentration="1">

<notes>

<body xmlns="<http://www.w3.org/1999/xhtml>">

<p>FORMULA: C20H29O4</p>

<p>CHARGE: -1</p>

<p>INCHI: InChI=1S/C20H30O4/c1-2-3-6-9-17(21)14-12-16-13-15-19(22)18(16)10-7-4-5-8-11-20(23)24/h4,7,12,14,17,21H,2-3,5-6,8-11,13,15H2,1H3,(H,23,24)/b7-4-,14-12+/t17-/m0/s1</p>

<p>EHMN_ABBREVIATION: C05954</p>

</body>

</notes>

</species>

<species boundaryCondition="false" charge="-1" constant="false" metaid="_metaM_HC02208_c" hasOnlySubstanceUnits="false" sboTerm="SBO:0000247" compartment="c" name="prostaglandin-d1" id="M_HC02208_c" initialConcentration="1">

<notes>

<body xmlns="<http://www.w3.org/1999/xhtml>">

<p>FORMULA: C20H33O5</p>

<p>CHARGE: -1</p>

<p>INCHI: InChI=1S/C20H34O5/c1-2-3-6-9-15(21)12-13-17-16(18(22)14-19(17)23)10-7-4-5-8-11-20(24)25/h12-13,15-18,21-22H,2-11,14H2,1H3,(H,24,25)/b13-12+/t15-,16+,17+,18-/m0/s1</p>

</body>

</notes>

</species>

<species boundaryCondition="false" charge="-1" constant="false" metaid="_metaM_HC02204_c" hasOnlySubstanceUnits="false" sboTerm="SBO:0000247" compartment="c" name="prostaglandin-b1" id="M_HC02204_c" initialConcentration="1">

<notes>

<body xmlns="<http://www.w3.org/1999/xhtml>">

<p>FORMULA: C20H31O4</p>

<p>CHARGE: -1</p>

<p>INCHI: InChI=1S/C20H32O4/c1-2-3-6-9-17(21)14-12-16-13-15-19(22)18(16)10-7-4-5-8-11-20(23)24/h12,14,17,21H,2-11,13,15H2,1H3,(H,23,24)/b14-12+/t17-/m0/s1</p>

<p>EHMN_ABBREVIATION: C00959</p>

</body>

</notes>

</species>

<species boundaryCondition="false" charge="-1" constant="false" metaid="_metaM_HC02214_c" hasOnlySubstanceUnits="false" sboTerm="SBO:0000247" compartment="c" name="prostaglandin-f1alpha" id="M_HC02214_c" initialConcentration="1">

<notes>

<body xmlns="<http://www.w3.org/1999/xhtml>">

<p>FORMULA: C20H35O5</p>

<p>CHARGE: -1</p>

<p>INCHI: InChI=1S/C20H36O5/c1-2-3-6-9-15(21)12-13-17-16(18(22)14-19(17)23)10-7-4-5-8-11-20(24)25/h12-13,15-19,21-23H,2-11,14H2,1H3,(H,24,25)/b13-12+/t15-,16+,17+,18-,19+/m0/s1</p>

</body>

</notes>

</species>

<species boundaryCondition="false" charge="-1" constant="false" metaid="_metaM_HC02213_c" hasOnlySubstanceUnits="false" sboTerm="SBO:0000247" compartment="c" name="prostaglandin-e3" id="M_HC02213_c" initialConcentration="1">

<notes>

<body xmlns="<http://www.w3.org/1999/xhtml>">

<p>FORMULA: C20H29O5</p>

<p>CHARGE: -1</p>

<p>INCHI: InChI=1S/C20H30O5/c1-2-3-6-9-15(21)12-13-17-16(18(22)14-19(17)23)10-7-4-5-8-11-20(24)25/h3-4,6-7,12-13,15-17,19,21,23H,2,5,8-11,14H2,1H3,(H,24,25)/b6-3-,7-4-,13-12+/t15-,16+,17+,19+/m0/s1</p>

<p>EHMN_ABBREVIATION: C06439</p>

</body>

</notes>

</species>

<species boundaryCondition="false" charge="-1" constant="false" metaid="_metaM_HC02216_c" hasOnlySubstanceUnits="false" sboTerm="SBO:0000247" compartment="c" name="prostaglandin-f2beta" id="M_HC02216_c" initialConcentration="1">

<notes>

<body xmlns="<http://www.w3.org/1999/xhtml>">

<p>FORMULA: C20H33O5</p>

<p>CHARGE: -1</p>

<p>INCHI: InChI=1S/C20H34O5/c1-2-3-6-9-15(21)12-13-17-16(18(22)14-19(17)23)10-7-4-5-8-11-20(24)25/h4,7,12-13,15-19,21-23H,2-3,5-6,8-11,14H2,1H3,(H,24,25)/b7-4-,13-12+/t15-,16+,17+,18+,19+/m0/s1</p>

</body>

</notes>

</species>

<species boundaryCondition="false" charge="-1" constant="false" metaid="_metaM_HC02210_c" hasOnlySubstanceUnits="false" sboTerm="SBO:0000247" compartment="c" name="prostaglandin-d3" id="M_HC02210_c" initialConcentration="1">

<notes>

<body xmlns="<http://www.w3.org/1999/xhtml>">

<p>FORMULA: C20H29O5</p>

<p>CHARGE: -1</p>

<p>INCHI: InChI=1S/C20H30O5/c1-2-3-6-9-15(21)12-13-17-16(18(22)14-19(17)23)10-7-4-5-8-11-20(24)25/h3-4,6-7,12-13,15-18,21-22H,2,5,8-11,14H2,1H3,(H,24,25)/b6-3-,7-4-,13-12+/t15-,16+,17+,18-/m0/s1</p>

</body>

</notes>

</species>

<species boundaryCondition="false" charge="-1" constant="false" metaid="_metaM_HC02217_c" hasOnlySubstanceUnits="false" sboTerm="SBO:0000247" compartment="c" name="prostaglandin-g2" id="M_HC02217_c" initialConcentration="1">

<notes>

<body xmlns="<http://www.w3.org/1999/xhtml>">

<p>FORMULA: C20H31O6</p>

<p>CHARGE: -1</p>

<p>INCHI: InChI=1S/C20H32O6/c1-2-3-6-9-15(24-23)12-13-17-16(18-14-19(17)26-25-18)10-7-4-5-8-11-20(21)22/h4,7,12-13,15-19,23H,2-3,5-6,8-11,14H2,1H3,(H,21,22)/b7-4-,13-12+/t15-,16+,17+,18-,19+/m0/s1</p>

<p>EHMN_ABBREVIATION: C05956</p>

</body>

</notes>

</species>

<species boundaryCondition="false" charge="0" constant="false" metaid="_metaM_HC02200_e" hasOnlySubstanceUnits="false" sboTerm="SBO:0000247" compartment="e" name="S-glutathionyl-2-4-dinitrobenzene" id="M_HC02200_e" initialConcentration="1">

<notes>

<body xmlns="<http://www.w3.org/1999/xhtml>">

<p>FORMULA: </p>

<p>CHARGE: 0</p>

<p>INCHI: </p>

</body>

</notes>

</species>

<species boundaryCondition="false" charge="0" constant="false" metaid="_metaM_HC02200_c" hasOnlySubstanceUnits="false" sboTerm="SBO:0000247" compartment="c" name="S-glutathionyl-2-4-dinitrobenzene" id="M_HC02200_c" initialConcentration="1">

<notes>

<body xmlns="<http://www.w3.org/1999/xhtml>">

<p>FORMULA: </p>

<p>CHARGE: 0</p>

<p>INCHI: </p>

</body>

</notes>

</species>

<species boundaryCondition="false" charge="0" constant="false" metaid="_metaM_HC02201_e" hasOnlySubstanceUnits="false" sboTerm="SBO:0000247" compartment="e" name="S-glutathionyl-ethacrynic-acid" id="M_HC02201_e" initialConcentration="1">

<notes>

<body xmlns="<http://www.w3.org/1999/xhtml>">

<p>FORMULA: </p>

<p>CHARGE: 0</p>

<p>INCHI: </p>

</body>

</notes>

</species>

<species boundaryCondition="false" charge="0" constant="false" metaid="_metaM_HC02201_c" hasOnlySubstanceUnits="false" sboTerm="SBO:0000247" compartment="c" name="S-glutathionyl-ethacrynic-acid" id="M_HC02201_c" initialConcentration="1">

<notes>

<body xmlns="<http://www.w3.org/1999/xhtml>">

<p>FORMULA: </p>

<p>CHARGE: 0</p>

<p>INCHI: </p>

</body>

</notes>

</species>

<species boundaryCondition="false" charge="-1" constant="false" metaid="_metaM_CE0955_r" hasOnlySubstanceUnits="false" sboTerm="SBO:0000247" compartment="r" name="6-oxo-prostaglandin F1alpha" id="M_CE0955_r" initialConcentration="1">

<notes>

<body xmlns="<http://www.w3.org/1999/xhtml>">

<p>FORMULA: C20H33O6</p>

<p>CHARGE: -1</p>

<p>INCHI: InChI=1S/C20H34O6/c1-2-3-4-7-14(21)10-11-16-17(19(24)13-18(16)23)12-15(22)8-5-6-9-20(25)26/h10-11,14,16-19,21,23-24H,2-9,12-13H2,1H3,(H,25,26)/b11-10+/t14-,16+,17+,18+,19-/m0/s1</p>

<p>EHMN_ABBREVIATION: CE0955</p>

</body>

</notes>

</species>

<species boundaryCondition="false" charge="-1" constant="false" metaid="_metaM_prostgi2_r" hasOnlySubstanceUnits="false" sboTerm="SBO:0000247" compartment="r" name="prostaglandin I2(1-)" id="M_prostgi2_r" initialConcentration="1">

<notes>

<body xmlns="<http://www.w3.org/1999/xhtml>">

<p>FORMULA: C20H31O5</p>

<p>CHARGE: -1</p>

<p>INCHI: InChI=1S/C20H32O5/c1-2-3-4-7-14(21)10-11-16-17-12-15(8-5-6-9-20(23)24)25-19(17)13-18(16)22/h8,10-11,14,16-19,21-22H,2-7,9,12-13H2,1H3,(H,23,24)/p-1/b11-10+,15-8-/t14-,16+,17+,18+,19-/m0/s1</p>

<p>EHMN_ABBREVIATION: C01312</p>

</body>

</notes>

</species>

<species boundaryCondition="false" charge="-1" constant="false" metaid="_metaM_txa2_r" hasOnlySubstanceUnits="false" sboTerm="SBO:0000247" compartment="r" name="thromboxane A2(1-)" id="M_txa2_r" initialConcentration="1">

<notes>

<body xmlns="<http://www.w3.org/1999/xhtml>">

<p>FORMULA: C20H31O5</p>

<p>CHARGE: -1</p>

<p>INCHI: InChI=1S/C20H32O5/c1-2-3-6-9-15(21)12-13-17-16(18-14-20(24-17)25-18)10-7-4-5-8-11-19(22)23/h4,7,12-13,15-18,20-21H,2-3,5-6,8-11,14H2,1H3,(H,22,23)/p-1/b7-4-,13-12+/t15-,16+,17+,18-,20+/m0/s1</p>

<p>EHMN_ABBREVIATION: C02198</p>

</body>

</notes>

</species>

<species boundaryCondition="false" charge="0" constant="false" metaid="_metaM_h2_c" hasOnlySubstanceUnits="false" sboTerm="SBO:0000247" compartment="c" name="Hydrogen" id="M_h2_c" initialConcentration="1">

<notes>

<body xmlns="<http://www.w3.org/1999/xhtml>">

<p>FORMULA: H2</p>

<p>CHARGE: 0</p>

<p>INCHI: InChI=1S/H2/h1H</p>

<p>EHMN_ABBREVIATION: C00282</p>

</body>

</notes>

</species>

<species boundaryCondition="false" charge="-1" constant="false" metaid="_metaM_CE1447_c" hasOnlySubstanceUnits="false" sboTerm="SBO:0000247" compartment="c" name="11-dehydrothromboxane B2" id="M_CE1447_c" initialConcentration="1">

<notes>

<body xmlns="<http://www.w3.org/1999/xhtml>">

<p>FORMULA: C20H31O6</p>

<p>CHARGE: -1</p>

<p>INCHI: InChI=1S/C20H32O6/c1-2-3-6-9-15(21)12-13-18-16(17(22)14-20(25)26-18)10-7-4-5-8-11-19(23)24/h4,7,12-13,15-18,21-22H,2-3,5-6,8-11,14H2,1H3,(H,23,24)/b7-4-,13-12+/t15-,16-,17-,18+/m0/s1</p>

<p>EHMN_ABBREVIATION: CE1447</p>

</body>

</notes>

</species>

<species boundaryCondition="false" charge="-4" constant="false" metaid="_metaM_CE4795_x" hasOnlySubstanceUnits="false" sboTerm="SBO:0000247" compartment="x" name="2-trans-cis,cis,cis,cis-4,8,11,14-eicosapentaenoyl-CoA" id="M_CE4795_x" initialConcentration="1">

<notes>

<body xmlns="<http://www.w3.org/1999/xhtml>">

<p>FORMULA: C41H60N7O17P3S</p>

<p>CHARGE: -4</p>

<p>INCHI: </p>

<p>EHMN_ABBREVIATION: CE4795</p>

</body>

</notes>

</species>

<species boundaryCondition="false" charge="-4" constant="false" metaid="_metaM_CE5115_x" hasOnlySubstanceUnits="false" sboTerm="SBO:0000247" compartment="x" name="trans-3-cis-5,8,11,14-eicosapentaenoyl-CoA" id="M_CE5115_x" initialConcentration="1">

<notes>

<body xmlns="<http://www.w3.org/1999/xhtml>">

<p>FORMULA: C41H60N7O17P3S</p>

<p>CHARGE: -4</p>

<p>INCHI: </p>

<p>EHMN_ABBREVIATION: CE5115</p>

</body>

</notes>

</species>

<species boundaryCondition="false" charge="-4" constant="false" metaid="_metaM_arachdcoa_x" hasOnlySubstanceUnits="false" sboTerm="SBO:0000247" compartment="x" name="arachidonoyl-CoA(4-)" id="M_arachdcoa_x" initialConcentration="1">

<notes>

<body xmlns="<http://www.w3.org/1999/xhtml>">

<p>FORMULA: C41H62N7O17P3S</p>

<p>CHARGE: -4</p>

<p>INCHI: InChI=1S/C41H66N7O17P3S/c1-4-5-6-7-8-9-10-11-12-13-14-15-16-17-18-19-20-21-32(50)69-25-24-43-31(49)22-23-44-39(53)36(52)41(2,3)27-62-68(59,60)65-67(57,58)61-26-30-35(64-66(54,55)56)34(51)40(63-30)48-29-47-33-37(42)45-28-46-38(33)48/h8-9,11-12,14-15,17-18,28-30,34-36,40,51-52H,4-7,10,13,16,19-27H2,1-3H3,(H,43,49)(H,44,53)(H,57,58)(H,59,60)(H2,42,45,46)(H2,54,55,56)/p-4/b9-8-,12-11-,15-14-,18-17-/t30-,34-,35-,36+,40-/m1/s1</p>

<p>HEPATONET_1.0_ABBREVIATION: HC01986</p>

<p>EHMN_ABBREVIATION: C02249</p>

</body>

</notes>

</species>

<species boundaryCondition="false" charge="-1" constant="false" metaid="_metaM_arachd_x" hasOnlySubstanceUnits="false" sboTerm="SBO:0000247" compartment="x" name="arachidonate" id="M_arachd_x" initialConcentration="1">

<notes>

<body xmlns="<http://www.w3.org/1999/xhtml>">

<p>FORMULA: C20H31O2</p>

<p>CHARGE: -1</p>

<p>INCHI: InChI=1S/C20H32O2/c1-2-3-4-5-6-7-8-9-10-11-12-13-14-15-16-17-18-19-20(21)22/h6-7,9-10,12-13,15-16H,2-5,8,11,14,17-19H2,1H3,(H,21,22)/p-1/b7-6-,10-9-,13-12-,16-15-</p>

<p>HEPATONET_1.0_ABBREVIATION: HC00202</p>

<p>EHMN_ABBREVIATION: C00219</p>

</body>

</notes>

</species>

<species boundaryCondition="false" charge="1" constant="false" metaid="_metaM_h_x" hasOnlySubstanceUnits="false" sboTerm="SBO:0000247" compartment="x" name="proton" id="M_h_x" initialConcentration="1">

<notes>

<body xmlns="<http://www.w3.org/1999/xhtml>">

<p>FORMULA: H</p>

<p>CHARGE: 1</p>

<p>HEPATONET_1.0_ABBREVIATION: HC00083</p>

<p>EHMN_ABBREVIATION: C00080</p>

<p>INCHI: InChI=1S/p+1/i/hH</p>

</body>

</notes>

</species>

<species boundaryCondition="false" charge="-4" constant="false" metaid="_metaM_coa_x" hasOnlySubstanceUnits="false" sboTerm="SBO:0000247" compartment="x" name="Coenzyme A" id="M_coa_x" initialConcentration="1">

<notes>

<body xmlns="<http://www.w3.org/1999/xhtml>">

<p>FORMULA: C21H32N7O16P3S</p>

<p>CHARGE: -4</p>

<p>HEPATONET_1.0_ABBREVIATION: HC00020</p>

<p>EHMN_ABBREVIATION: C00010</p>

<p>INCHI: InChI=1S/C21H36N7O16P3S/c1-21(2,16(31)19(32)24-4-3-12(29)23-5-6-48)8-41-47(38,39)44-46(36,37)40-7-11-15(43-45(33,34)35)14(30)20(42-11)28-10-27-13-17(22)25-9-26-18(13)28/h9-11,14-16,20,30-31,48H,3-8H2,1-2H3,(H,23,29)(H,24,32)(H,36,37)(H,38,39)(H2,22,25,26)(H2,33,34,35)/p-4/t11-,14-,15-,16+,20-/m1/s1</p>

</body>

</notes>

</species>

<species boundaryCondition="false" charge="0" constant="false" metaid="_metaM_o2_x" hasOnlySubstanceUnits="false" sboTerm="SBO:0000247" compartment="x" name="O2" id="M_o2_x" initialConcentration="1">

<notes>

<body xmlns="<http://www.w3.org/1999/xhtml>">

<p>FORMULA: O2</p>

<p>CHARGE: 0</p>

<p>HEPATONET_1.0_ABBREVIATION: HC00017</p>

<p>EHMN_ABBREVIATION: C00007</p>

<p>INCHI: InChI=1S/O2/c1-2</p>

</body>

</notes>

</species>

<species boundaryCondition="false" charge="0" constant="false" metaid="_metaM_h2o_x" hasOnlySubstanceUnits="false" sboTerm="SBO:0000247" compartment="x" name="H2O" id="M_h2o_x" initialConcentration="1">

<notes>

<body xmlns="<http://www.w3.org/1999/xhtml>">

<p>FORMULA: H2O</p>

<p>CHARGE: 0</p>

<p>HEPATONET_1.0_ABBREVIATION: HC00011</p>

<p>EHMN_ABBREVIATION: C00001</p>

<p>INCHI: InChI=1S/H2O/h1H2</p>

</body>

</notes>

</species>

<species boundaryCondition="false" charge="-4" constant="false" metaid="_metaM_CE5116_x" hasOnlySubstanceUnits="false" sboTerm="SBO:0000247" compartment="x" name="trans-3-cis-8,11,14-eicosatetraenoyl-CoA" id="M_CE5116_x" initialConcentration="1">

<notes>

<body xmlns="<http://www.w3.org/1999/xhtml>">

<p>FORMULA: C41H62N7O17P3S</p>

<p>CHARGE: -4</p>

<p>INCHI: </p>

<p>EHMN_ABBREVIATION: CE5116</p>

</body>

</notes>

</species>

<species boundaryCondition="false" charge="-4" constant="false" metaid="_metaM_nadph_x" hasOnlySubstanceUnits="false" sboTerm="SBO:0000247" compartment="x" name="Nicotinamide adenine dinucleotide phosphate - reduced" id="M_nadph_x" initialConcentration="1">

<notes>

<body xmlns="<http://www.w3.org/1999/xhtml>">

<p>FORMULA: C21H26N7O17P3</p>

<p>CHARGE: -4</p>

<p>HEPATONET_1.0_ABBREVIATION: HC00015</p>

<p>EHMN_ABBREVIATION: C00005</p>

<p>INCHI: InChI=1S/C21H30N7O17P3/c22-17-12-19(25-7-24-17)28(8-26-12)21-16(44-46(33,34)35)14(30)11(43-21)6-41-48(38,39)45-47(36,37)40-5-10-13(29)15(31)20(42-10)27-3-1-2-9(4-27)18(23)32/h1,3-4,7-8,10-11,13-16,20-21,29-31H,2,5-6H2,(H2,23,32)(H,36,37)(H,38,39)(H2,22,24,25)(H2,33,34,35)/p-4/t10-,11-,13-,14-,15-,16-,20-,21-/m1/s1</p>

</body>

</notes>

</species>

<species boundaryCondition="false" charge="-3" constant="false" metaid="_metaM_nadp_x" hasOnlySubstanceUnits="false" sboTerm="SBO:0000247" compartment="x" name="Nicotinamide adenine dinucleotide phosphate" id="M_nadp_x" initialConcentration="1">

<notes>

<body xmlns="<http://www.w3.org/1999/xhtml>">

<p>FORMULA: C21H25N7O17P3</p>

<p>CHARGE: -3</p>

<p>HEPATONET_1.0_ABBREVIATION: HC00016</p>

<p>EHMN_ABBREVIATION: C00006</p>

<p>INCHI: InChI=1S/C21H28N7O17P3/c22-17-12-19(25-7-24-17)28(8-26-12)21-16(44-46(33,34)35)14(30)11(43-21)6-41-48(38,39)45-47(36,37)40-5-10-13(29)15(31)20(42-10)27-3-1-2-9(4-27)18(23)32/h1-4,7-8,10-11,13-16,20-21,29-31H,5-6H2,(H7-,22,23,24,25,32,33,34,35,36,37,38,39)/p-3/t10-,11-,13-,14-,15-,16-,20-,21-/m1/s1</p>

</body>

</notes>

</species>

<species boundaryCondition="false" charge="-1" constant="false" metaid="_metaM_arachd_l" hasOnlySubstanceUnits="false" sboTerm="SBO:0000247" compartment="l" name="arachidonate" id="M_arachd_l" initialConcentration="1">

<notes>

<body xmlns="<http://www.w3.org/1999/xhtml>">

<p>FORMULA: C20H31O2</p>

<p>CHARGE: -1</p>

<p>INCHI: InChI=1S/C20H32O2/c1-2-3-4-5-6-7-8-9-10-11-12-13-14-15-16-17-18-19-20(21)22/h6-7,9-10,12-13,15-16H,2-5,8,11,14,17-19H2,1H3,(H,21,22)/p-1/b7-6-,10-9-,13-12-,16-15-</p>

<p>HEPATONET_1.0_ABBREVIATION: HC00202</p>

<p>EHMN_ABBREVIATION: C00219</p>

</body>

</notes>

</species>

</listOfSpecies>

<listOfReactions>

<reaction name="Arachidonate 15-lipoxygenase" id="R_ALOX15" metaid="_metaR_ALOX15" reversible="false" sboTerm="SBO:0000176">

<notes>

<body xmlns="<http://www.w3.org/1999/xhtml>">

<p>GENE_ASSOCIATION: (247.1) or (246.1)</p>

<p>SUBSYSTEM: Eicosanoid metabolism</p>

<p>EC Number: 1.13.11.33</p>

<p>Confidence Level: 4</p>

<p>AUTHORS: PMID:11839751</p>

<p>NOTES: Converts arachidonic acid to 15S-hydroperoxyeicosatetraenoic acid. Also acts on C-12 of arachidonate as well as on linoleic acid. NJ</p>

</body>

</notes>

<listOfReactants>

<speciesReference species="M_arachd_c"/>

<speciesReference species="M_o2_c"/>

</listOfReactants>

<listOfProducts>

<speciesReference species="M_15HPET_c"/>

</listOfProducts>

<kineticLaw>

<math xmlns="<http://www.w3.org/1998/Math/MathML>">

<ci> FLUX_VALUE </ci>

</math>

<listOfParameters>

<parameter id="LOWER_BOUND" units="mmol_per_gDW_per_hr" value="0"/>

<parameter id="UPPER_BOUND" units="mmol_per_gDW_per_hr" value="1000"/>

<parameter id="FLUX_VALUE" units="mmol_per_gDW_per_hr" value="0"/>

<parameter id="OBJECTIVE_COEFFICIENT" units="dimensionless" value="0"/>

</listOfParameters>

</kineticLaw>

</reaction>

<reaction name="2,3-dioxo-L-gulonate:hydrogen peroxide oxireductase" id="R_DOGULNO1" metaid="_metaR_DOGULNO1" reversible="false" sboTerm="SBO:0000176">

<notes>

<body xmlns="<http://www.w3.org/1999/xhtml>">

<p>GENE_ASSOCIATION: </p>

<p>SUBSYSTEM: Vitamin C metabolism</p>

<p>EC Number: </p>

<p>Confidence Level: 2</p>

<p>AUTHORS: PMID:10727845,PMID:9296457</p>

<p>NOTES: NCD</p>

</body>

</notes>

<listOfReactants>

<speciesReference species="M_h2o2_c"/>

</listOfReactants>

<listOfProducts>

<speciesReference species="M_thrnt_c"/>

<speciesReference species="M_h_c" stoichiometry="2"/>

</listOfProducts>

<kineticLaw>

<math xmlns="<http://www.w3.org/1998/Math/MathML>">

<ci> FLUX_VALUE </ci>

</math>

<listOfParameters>

<parameter id="LOWER_BOUND" units="mmol_per_gDW_per_hr" value="0"/>

<parameter id="UPPER_BOUND" units="mmol_per_gDW_per_hr" value="1000"/>

<parameter id="FLUX_VALUE" units="mmol_per_gDW_per_hr" value="0"/>

<parameter id="OBJECTIVE_COEFFICIENT" units="dimensionless" value="0"/>

</listOfParameters>

</kineticLaw>

</reaction>

<reaction name="RE3033" id="R_RE3033C" metaid="_metaR_RE3033C" reversible="false" sboTerm="SBO:0000176">

<notes>

<body xmlns="<http://www.w3.org/1999/xhtml>">

<p>GENE_ASSOCIATION: </p>

<p>SUBSYSTEM: Arachidonic acid metabolism</p>

<p>EC Number: 1.13.11.34</p>

<p>Confidence Level: 0</p>

<p>AUTHORS: PMID:11323741,PMID:9870464</p>

<p>NOTES: </p>

</body>

</notes>

<listOfReactants>

<speciesReference species="M_o2_c"/>

<speciesReference species="M_15HPET_c"/>

</listOfReactants>

<listOfProducts>

<speciesReference species="M_h2o2_c"/>

<speciesReference species="M_CE2567_c"/>

</listOfProducts>

<kineticLaw>

<math xmlns="<http://www.w3.org/1998/Math/MathML>">

<ci> FLUX_VALUE </ci>

</math>

<listOfParameters>

<parameter id="LOWER_BOUND" units="mmol_per_gDW_per_hr" value="0"/>

<parameter id="UPPER_BOUND" units="mmol_per_gDW_per_hr" value="1000"/>

<parameter id="FLUX_VALUE" units="mmol_per_gDW_per_hr" value="0"/>

<parameter id="OBJECTIVE_COEFFICIENT" units="dimensionless" value="0"/>

</listOfParameters>

</kineticLaw>

</reaction>

<reaction name="RE1050" id="R_RE1050C" metaid="_metaR_RE1050C" reversible="true" sboTerm="SBO:0000176">

<notes>

<body xmlns="<http://www.w3.org/1999/xhtml>">

<p>GENE_ASSOCIATION: (7173.1) or (9588.1)</p>

<p>SUBSYSTEM: Miscellaneous</p>

<p>EC Number: 1.11.1.7</p>

<p>Confidence Level: 0</p>

<p>AUTHORS: PMID:11013238</p>

<p>NOTES: </p>

</body>

</notes>

<listOfReactants>

<speciesReference species="M_h2o2_c" stoichiometry="3"/>

</listOfReactants>

<listOfProducts>

<speciesReference species="M_h2o_c" stoichiometry="2"/>

<speciesReference species="M_cynt_c" stoichiometry="2"/>

<speciesReference species="M_h_c" stoichiometry="2"/>

</listOfProducts>

<kineticLaw>

<math xmlns="<http://www.w3.org/1998/Math/MathML>">

<ci> FLUX_VALUE </ci>

</math>

<listOfParameters>

<parameter id="LOWER_BOUND" units="mmol_per_gDW_per_hr" value="-1000"/>

<parameter id="UPPER_BOUND" units="mmol_per_gDW_per_hr" value="1000"/>

<parameter id="FLUX_VALUE" units="mmol_per_gDW_per_hr" value="0"/>

<parameter id="OBJECTIVE_COEFFICIENT" units="dimensionless" value="0"/>

</listOfParameters>

</kineticLaw>

</reaction>

<reaction name="RE0702" id="R_RE0702C" metaid="_metaR_RE0702C" reversible="true" sboTerm="SBO:0000176">

<notes>

<body xmlns="<http://www.w3.org/1999/xhtml>">

<p>GENE_ASSOCIATION: (7173.1) or (9588.1)</p>

<p>SUBSYSTEM: Miscellaneous</p>

<p>EC Number: 1.8.1.4</p>

<p>Confidence Level: 0</p>

<p>AUTHORS: PMID:11013238,PMID:9665099</p>

<p>NOTES: </p>

</body>

</notes>

<listOfReactants>

<speciesReference species="M_h2o2_c"/>

<speciesReference species="M_h_c"/>

</listOfReactants>

<listOfProducts>

<speciesReference species="M_CE2011_c"/>

<speciesReference species="M_h2o_c"/>

</listOfProducts>

<kineticLaw>

<math xmlns="<http://www.w3.org/1998/Math/MathML>">

<ci> FLUX_VALUE </ci>

</math>

<listOfParameters>

<parameter id="LOWER_BOUND" units="mmol_per_gDW_per_hr" value="-1000"/>

<parameter id="UPPER_BOUND" units="mmol_per_gDW_per_hr" value="1000"/>

<parameter id="FLUX_VALUE" units="mmol_per_gDW_per_hr" value="0"/>

<parameter id="OBJECTIVE_COEFFICIENT" units="dimensionless" value="0"/>

</listOfParameters>

</kineticLaw>

</reaction>

<reaction name="RE2440" id="R_RE2440C" metaid="_metaR_RE2440C" reversible="true" sboTerm="SBO:0000176">

<notes>

<body xmlns="<http://www.w3.org/1999/xhtml>">

<p>GENE_ASSOCIATION: </p>

<p>SUBSYSTEM: Tryptophan metabolism</p>

<p>EC Number: </p>

<p>Confidence Level: 0</p>

<p>AUTHORS: PMID:12044950</p>

<p>NOTES: </p>

</body>

</notes>

<listOfReactants>

<speciesReference species="M_h2o2_c"/>

</listOfReactants>

<listOfProducts>

<speciesReference species="M_CE5860_c" stoichiometry="2"/>

<speciesReference species="M_h_c"/>

</listOfProducts>

<kineticLaw>

<math xmlns="<http://www.w3.org/1998/Math/MathML>">

<ci> FLUX_VALUE </ci>

</math>

<listOfParameters>

<parameter id="LOWER_BOUND" units="mmol_per_gDW_per_hr" value="-1000"/>

<parameter id="UPPER_BOUND" units="mmol_per_gDW_per_hr" value="1000"/>

<parameter id="FLUX_VALUE" units="mmol_per_gDW_per_hr" value="0"/>

<parameter id="OBJECTIVE_COEFFICIENT" units="dimensionless" value="0"/>

</listOfParameters>

</kineticLaw>

</reaction>

<reaction name="RE2888" id="R_RE2888C" metaid="_metaR_RE2888C" reversible="true" sboTerm="SBO:0000176">

<notes>

<body xmlns="<http://www.w3.org/1999/xhtml>">

<p>GENE_ASSOCIATION: (7173.1) or (9588.1)</p>

<p>SUBSYSTEM: Purine catabolism</p>

<p>EC Number: 1.11.1.7</p>

<p>Confidence Level: 0</p>

<p>AUTHORS: PMID:10600166</p>

<p>NOTES: </p>

</body>

</notes>

<listOfReactants>

<speciesReference species="M_h2o2_c"/>

</listOfReactants>

<listOfProducts>

<speciesReference species="M_CE0074_c"/>

</listOfProducts>

<kineticLaw>

<math xmlns="<http://www.w3.org/1998/Math/MathML>">

<ci> FLUX_VALUE </ci>

</math>

<listOfParameters>

<parameter id="LOWER_BOUND" units="mmol_per_gDW_per_hr" value="-1000"/>

<parameter id="UPPER_BOUND" units="mmol_per_gDW_per_hr" value="1000"/>

<parameter id="FLUX_VALUE" units="mmol_per_gDW_per_hr" value="0"/>

<parameter id="OBJECTIVE_COEFFICIENT" units="dimensionless" value="0"/>

</listOfParameters>

</kineticLaw>

</reaction>

<reaction name="RE3038" id="R_RE3038C" metaid="_metaR_RE3038C" reversible="true" sboTerm="SBO:0000176">

<notes>

<body xmlns="<http://www.w3.org/1999/xhtml>">

<p>GENE_ASSOCIATION: 2053.1</p>

<p>SUBSYSTEM: Arachidonic acid metabolism</p>

<p>EC Number: 3.3.2.9</p>

<p>Confidence Level: 0</p>

<p>AUTHORS: PMID:11323741,PMID:9870464</p>

<p>NOTES: </p>

</body>

</notes>

<listOfReactants>

<speciesReference species="M_CE2567_c"/>

<speciesReference species="M_h2o_c"/>

</listOfReactants>

<listOfProducts>

<speciesReference species="M_C06315_c"/>

</listOfProducts>

<kineticLaw>

<math xmlns="<http://www.w3.org/1998/Math/MathML>">

<ci> FLUX_VALUE </ci>

</math>

<listOfParameters>

<parameter id="LOWER_BOUND" units="mmol_per_gDW_per_hr" value="-1000"/>

<parameter id="UPPER_BOUND" units="mmol_per_gDW_per_hr" value="1000"/>

<parameter id="FLUX_VALUE" units="mmol_per_gDW_per_hr" value="0"/>

<parameter id="OBJECTIVE_COEFFICIENT" units="dimensionless" value="0"/>

</listOfParameters>

</kineticLaw>

</reaction>

<reaction name="RE3040" id="R_RE3040C" metaid="_metaR_RE3040C" reversible="true" sboTerm="SBO:0000176">

<notes>

<body xmlns="<http://www.w3.org/1999/xhtml>">

<p>GENE_ASSOCIATION: 2053.1</p>

<p>SUBSYSTEM: Arachidonic acid metabolism</p>

<p>EC Number: 3.3.2.9</p>

<p>Confidence Level: 0</p>

<p>AUTHORS: PMID:11323741,PMID:9870464</p>

<p>NOTES: </p>

</body>

</notes>

<listOfReactants>

<speciesReference species="M_CE2567_c"/>

<speciesReference species="M_h2o_c"/>

</listOfReactants>

<listOfProducts>

<speciesReference species="M_C06314_c"/>

</listOfProducts>

<kineticLaw>

<math xmlns="<http://www.w3.org/1998/Math/MathML>">

<ci> FLUX_VALUE </ci>

</math>

<listOfParameters>

<parameter id="LOWER_BOUND" units="mmol_per_gDW_per_hr" value="-1000"/>

<parameter id="UPPER_BOUND" units="mmol_per_gDW_per_hr" value="1000"/>

<parameter id="FLUX_VALUE" units="mmol_per_gDW_per_hr" value="0"/>

<parameter id="OBJECTIVE_COEFFICIENT" units="dimensionless" value="0"/>

</listOfParameters>

</kineticLaw>

</reaction>

<reaction name="RE3036" id="R_RE3036C" metaid="_metaR_RE3036C" reversible="true" sboTerm="SBO:0000176">

<notes>

<body xmlns="<http://www.w3.org/1999/xhtml>">

<p>GENE_ASSOCIATION: 239.1</p>

<p>SUBSYSTEM: Arachidonic acid metabolism</p>

<p>EC Number: 1.13.11.31</p>

<p>Confidence Level: 0</p>

<p>AUTHORS: PMID:10224163</p>

<p>NOTES: </p>

</body>

</notes>

<listOfReactants>

<speciesReference species="M_15HPET_c"/>

</listOfReactants>

<listOfProducts>

<speciesReference species="M_CE7172_c"/>

</listOfProducts>

<kineticLaw>

<math xmlns="<http://www.w3.org/1998/Math/MathML>">

<ci> FLUX_VALUE </ci>

</math>

<listOfParameters>

<parameter id="LOWER_BOUND" units="mmol_per_gDW_per_hr" value="-1000"/>

<parameter id="UPPER_BOUND" units="mmol_per_gDW_per_hr" value="1000"/>

<parameter id="FLUX_VALUE" units="mmol_per_gDW_per_hr" value="0"/>

<parameter id="OBJECTIVE_COEFFICIENT" units="dimensionless" value="0"/>

</listOfParameters>

</kineticLaw>

</reaction>

<reaction name="RE3044" id="R_RE3044C" metaid="_metaR_RE3044C" reversible="false" sboTerm="SBO:0000176">

<notes>

<body xmlns="<http://www.w3.org/1999/xhtml>">

<p>GENE_ASSOCIATION: </p>

<p>SUBSYSTEM: Arachidonic acid metabolism</p>

<p>EC Number: 1.13.11.34</p>

<p>Confidence Level: 0</p>

<p>AUTHORS: PMID:11323741,PMID:9870464</p>

<p>NOTES: </p>

</body>

</notes>

<listOfReactants>

<speciesReference species="M_o2_c"/>

<speciesReference species="M_CE7172_c" stoichiometry="2"/>

</listOfReactants>

<listOfProducts>

<speciesReference species="M_C06315_c" stoichiometry="2"/>

</listOfProducts>

<kineticLaw>

<math xmlns="<http://www.w3.org/1998/Math/MathML>">

<ci> FLUX_VALUE </ci>

</math>

<listOfParameters>

<parameter id="LOWER_BOUND" units="mmol_per_gDW_per_hr" value="0"/>

<parameter id="UPPER_BOUND" units="mmol_per_gDW_per_hr" value="1000"/>

<parameter id="FLUX_VALUE" units="mmol_per_gDW_per_hr" value="0"/>

<parameter id="OBJECTIVE_COEFFICIENT" units="dimensionless" value="0"/>

</listOfParameters>

</kineticLaw>

</reaction>

<reaction name="Arachidonate 12-lipoxygenase R" id="R_ALOX12R" metaid="_metaR_ALOX12R" reversible="false" sboTerm="SBO:0000176">

<notes>

<body xmlns="<http://www.w3.org/1999/xhtml>">

<p>GENE_ASSOCIATION: 242.1</p>

<p>SUBSYSTEM: Eicosanoid metabolism</p>

<p>EC Number: 1.13.11.31</p>

<p>Confidence Level: 4</p>

<p>AUTHORS: PMID:9618483</p>

<p>NOTES: </p>

</body>

</notes>

<listOfReactants>

<speciesReference species="M_o2_c"/>

<speciesReference species="M_arachd_c"/>

</listOfReactants>

<listOfProducts>

<speciesReference species="M_12RHPET_c"/>

</listOfProducts>

<kineticLaw>

<math xmlns="<http://www.w3.org/1998/Math/MathML>">

<ci> FLUX_VALUE </ci>

</math>

<listOfParameters>

<parameter id="LOWER_BOUND" units="mmol_per_gDW_per_hr" value="0"/>

<parameter id="UPPER_BOUND" units="mmol_per_gDW_per_hr" value="1000"/>

<parameter id="FLUX_VALUE" units="mmol_per_gDW_per_hr" value="0"/>

<parameter id="OBJECTIVE_COEFFICIENT" units="dimensionless" value="0"/>

</listOfParameters>

</kineticLaw>

</reaction>

<reaction name="Arachidonate 12-lipoxygenase" id="R_ALOX12" metaid="_metaR_ALOX12" reversible="false" sboTerm="SBO:0000176">

<notes>

<body xmlns="<http://www.w3.org/1999/xhtml>">

<p>GENE_ASSOCIATION: 239.1</p>

<p>SUBSYSTEM: Eicosanoid metabolism</p>

<p>EC Number: 1.13.11.31</p>

<p>Confidence Level: 4</p>

<p>AUTHORS: PMID:2217179,PMID:2377602</p>

<p>NOTES: NJ</p>

</body>

</notes>

<listOfReactants>

<speciesReference species="M_arachd_c"/>

<speciesReference species="M_o2_c"/>

</listOfReactants>

<listOfProducts>

<speciesReference species="M_12HPET_c"/>

</listOfProducts>

<kineticLaw>

<math xmlns="<http://www.w3.org/1998/Math/MathML>">

<ci> FLUX_VALUE </ci>

</math>

<listOfParameters>

<parameter id="LOWER_BOUND" units="mmol_per_gDW_per_hr" value="0"/>

<parameter id="UPPER_BOUND" units="mmol_per_gDW_per_hr" value="1000"/>

<parameter id="FLUX_VALUE" units="mmol_per_gDW_per_hr" value="0"/>

<parameter id="OBJECTIVE_COEFFICIENT" units="dimensionless" value="0"/>

</listOfParameters>

</kineticLaw>

</reaction>

<reaction name="Prostaglandin G/H synthase" id="R_PGSr" metaid="_metaR_PGSr" reversible="false" sboTerm="SBO:0000176">

<notes>

<body xmlns="<http://www.w3.org/1999/xhtml>">

<p>GENE_ASSOCIATION: (5743.1) or (5742.1)</p>

<p>SUBSYSTEM: Eicosanoid metabolism</p>

<p>EC Number: 1.14.99.1</p>

<p>Confidence Level: 4</p>

<p>AUTHORS: PMID:14511332,PMID:1907252</p>

<p>NOTES: NJ</p>

</body>

</notes>

<listOfReactants>

<speciesReference species="M_o2_r" stoichiometry="2"/>

<speciesReference species="M_arachd_r"/>

<speciesReference species="M_h_r"/>

<speciesReference species="M_nadph_r"/>

</listOfReactants>

<listOfProducts>

<speciesReference species="M_prostgh2_r"/>

<speciesReference species="M_h2o_r"/>

<speciesReference species="M_nadp_r"/>

</listOfProducts>

<kineticLaw>

<math xmlns="<http://www.w3.org/1998/Math/MathML>">

<ci> FLUX_VALUE </ci>

</math>

<listOfParameters>

<parameter id="LOWER_BOUND" units="mmol_per_gDW_per_hr" value="0"/>

<parameter id="UPPER_BOUND" units="mmol_per_gDW_per_hr" value="1000"/>

<parameter id="FLUX_VALUE" units="mmol_per_gDW_per_hr" value="0"/>

<parameter id="OBJECTIVE_COEFFICIENT" units="dimensionless" value="0"/>

</listOfParameters>

</kineticLaw>

</reaction>

<reaction name="Prostaglandin E synthase" id="R_PGESr" metaid="_metaR_PGESr" reversible="true" sboTerm="SBO:0000176">

<notes>

<body xmlns="<http://www.w3.org/1999/xhtml>">

<p>GENE_ASSOCIATION: (80142.4) or (80142.2) or (9536.1) or (80142.1) or (9536.2) or (80142.3)</p>

<p>SUBSYSTEM: Eicosanoid metabolism</p>

<p>EC Number: 5.3.99.3</p>

<p>Confidence Level: 4</p>

<p>AUTHORS: PMID:11847219</p>

<p>NOTES: NJ</p>

</body>

</notes>

<listOfReactants>

<speciesReference species="M_prostgh2_r"/>

</listOfReactants>

<listOfProducts>

<speciesReference species="M_prostge2_r"/>

</listOfProducts>

<kineticLaw>

<math xmlns="<http://www.w3.org/1998/Math/MathML>">

<ci> FLUX_VALUE </ci>

</math>

<listOfParameters>

<parameter id="LOWER_BOUND" units="mmol_per_gDW_per_hr" value="-1000"/>

<parameter id="UPPER_BOUND" units="mmol_per_gDW_per_hr" value="1000"/>

<parameter id="FLUX_VALUE" units="mmol_per_gDW_per_hr" value="0"/>

<parameter id="OBJECTIVE_COEFFICIENT" units="dimensionless" value="0"/>

</listOfParameters>

</kineticLaw>

</reaction>

<reaction name="intracellular transport" id="R_ARACHDtr" metaid="_metaR_ARACHDtr" reversible="true" sboTerm="SBO:0000185">

<notes>

<body xmlns="<http://www.w3.org/1999/xhtml>">

<p>GENE_ASSOCIATION: </p>

<p>SUBSYSTEM: Transport, endoplasmic reticular</p>

<p>EC Number: </p>

<p>Confidence Level: 1</p>

<p>AUTHORS: </p>

<p>NOTES: unknown mechanism, presumed diffusion NJ</p>

</body>

</notes>

<listOfReactants>

<speciesReference species="M_arachd_c"/>

</listOfReactants>

<listOfProducts>

<speciesReference species="M_arachd_r"/>

</listOfProducts>

<kineticLaw>

<math xmlns="<http://www.w3.org/1998/Math/MathML>">

<ci> FLUX_VALUE </ci>

</math>

<listOfParameters>

<parameter id="LOWER_BOUND" units="mmol_per_gDW_per_hr" value="-1000"/>

<parameter id="UPPER_BOUND" units="mmol_per_gDW_per_hr" value="1000"/>

<parameter id="FLUX_VALUE" units="mmol_per_gDW_per_hr" value="0"/>

<parameter id="OBJECTIVE_COEFFICIENT" units="dimensionless" value="0"/>

</listOfParameters>

</kineticLaw>

</reaction>

<reaction name="cytochrome p450 4F8" id="R_P4504F81r" metaid="_metaR_P4504F81r" reversible="false" sboTerm="SBO:0000176">

<notes>

<body xmlns="<http://www.w3.org/1999/xhtml>">

<p>GENE_ASSOCIATION: 11283.1</p>

<p>SUBSYSTEM: Eicosanoid metabolism</p>

<p>EC Number: </p>

<p>Confidence Level: 4</p>

<p>AUTHORS: PMID:10405341,PMID:15579107</p>

<p>NOTES: w-2 hydroxylation of arachidonic acid -&amp;gt; 19R-Hydroxyprostaglandins NJ</p>

</body>

</notes>

<listOfReactants>

<speciesReference species="M_arachd_r"/>

<speciesReference species="M_o2_r"/>

<speciesReference species="M_h_r"/>

<speciesReference species="M_nadph_r"/>

</listOfReactants>

<listOfProducts>

<speciesReference species="M_18harachd_r"/>

<speciesReference species="M_h2o_r"/>

<speciesReference species="M_nadp_r"/>

</listOfProducts>

<kineticLaw>

<math xmlns="<http://www.w3.org/1998/Math/MathML>">

<ci> FLUX_VALUE </ci>

</math>

<listOfParameters>

<parameter id="LOWER_BOUND" units="mmol_per_gDW_per_hr" value="0"/>

<parameter id="UPPER_BOUND" units="mmol_per_gDW_per_hr" value="1000"/>

<parameter id="FLUX_VALUE" units="mmol_per_gDW_per_hr" value="0"/>

<parameter id="OBJECTIVE_COEFFICIENT" units="dimensionless" value="0"/>

</listOfParameters>

</kineticLaw>

</reaction>

<reaction name="cytochrome P450 4B1" id="R_P4504B1r" metaid="_metaR_P4504B1r" reversible="false" sboTerm="SBO:0000176">

<notes>

<body xmlns="<http://www.w3.org/1999/xhtml>">

<p>GENE_ASSOCIATION: 1572.1</p>

<p>SUBSYSTEM: Eicosanoid metabolism</p>

<p>EC Number: 1.14.14.1</p>

<p>Confidence Level: 4</p>

<p>AUTHORS: PMID:11996015,PMID:15579107,PMID:2574990</p>

<p>NOTES: _12 hydroxylation of arachidonic acid NJ</p>

</body>

</notes>

<listOfReactants>

<speciesReference species="M_o2_r"/>

<speciesReference species="M_arachd_r"/>

<speciesReference species="M_h_r"/>

<speciesReference species="M_nadph_r"/>

</listOfReactants>

<listOfProducts>

<speciesReference species="M_12harachd_r"/>

<speciesReference species="M_h2o_r"/>

<speciesReference species="M_nadp_r"/>

</listOfProducts>

<kineticLaw>

<math xmlns="<http://www.w3.org/1998/Math/MathML>">

<ci> FLUX_VALUE </ci>

</math>

<listOfParameters>

<parameter id="LOWER_BOUND" units="mmol_per_gDW_per_hr" value="0"/>

<parameter id="UPPER_BOUND" units="mmol_per_gDW_per_hr" value="1000"/>

<parameter id="FLUX_VALUE" units="mmol_per_gDW_per_hr" value="0"/>

<parameter id="OBJECTIVE_COEFFICIENT" units="dimensionless" value="0"/>

</listOfParameters>

</kineticLaw>

</reaction>

<reaction name="cytochrome p450 4F12/4F2" id="R_P4504F121r" metaid="_metaR_P4504F121r" reversible="false" sboTerm="SBO:0000176">

<notes>

<body xmlns="<http://www.w3.org/1999/xhtml>">

<p>GENE_ASSOCIATION: 66002.1</p>

<p>SUBSYSTEM: Eicosanoid metabolism</p>

<p>EC Number: </p>

<p>Confidence Level: 4</p>

<p>AUTHORS: PMID:15579107</p>

<p>NOTES: omega hydroxylation of arachidonic acid NJ</p>

</body>

</notes>

<listOfReactants>

<speciesReference species="M_o2_r"/>

<speciesReference species="M_arachd_r"/>

<speciesReference species="M_h_r"/>

<speciesReference species="M_nadph_r"/>

</listOfReactants>

<listOfProducts>

<speciesReference species="M_wharachd_r"/>

<speciesReference species="M_h2o_r"/>

<speciesReference species="M_nadp_r"/>

</listOfProducts>

<kineticLaw>

<math xmlns="<http://www.w3.org/1998/Math/MathML>">

<ci> FLUX_VALUE </ci>

</math>

<listOfParameters>

<parameter id="LOWER_BOUND" units="mmol_per_gDW_per_hr" value="0"/>

<parameter id="UPPER_BOUND" units="mmol_per_gDW_per_hr" value="1000"/>

<parameter id="FLUX_VALUE" units="mmol_per_gDW_per_hr" value="0"/>

<parameter id="OBJECTIVE_COEFFICIENT" units="dimensionless" value="0"/>

</listOfParameters>

</kineticLaw>

</reaction>

<reaction name="EC:6.2.1.3" id="R_r1262" metaid="_metaR_r1262" reversible="false" sboTerm="SBO:0000176">

<notes>

<body xmlns="<http://www.w3.org/1999/xhtml>">

<p>GENE_ASSOCIATION: </p>

<p>SUBSYSTEM: Fatty acid oxidation</p>

<p>EC Number: 6.2.1.3</p>

<p>Confidence Level: 0</p>

<p>AUTHORS: PMID:8584017</p>

<p>NOTES: </p>

</body>

</notes>

<listOfReactants>

<speciesReference species="M_atp_r"/>

<speciesReference species="M_arachd_r"/>

<speciesReference species="M_coa_r"/>

</listOfReactants>

<listOfProducts>

<speciesReference species="M_arachdcoa_r"/>

</listOfProducts>

<kineticLaw>

<math xmlns="<http://www.w3.org/1998/Math/MathML>">

<ci> FLUX_VALUE </ci>

</math>

<listOfParameters>

<parameter id="LOWER_BOUND" units="mmol_per_gDW_per_hr" value="0"/>

<parameter id="UPPER_BOUND" units="mmol_per_gDW_per_hr" value="1000"/>

<parameter id="FLUX_VALUE" units="mmol_per_gDW_per_hr" value="0"/>

<parameter id="OBJECTIVE_COEFFICIENT" units="dimensionless" value="0"/>

</listOfParameters>

</kineticLaw>

</reaction>

<reaction name="RE3151" id="R_RE3151R" metaid="_metaR_RE3151R" reversible="false" sboTerm="SBO:0000176">

<notes>

<body xmlns="<http://www.w3.org/1999/xhtml>">

<p>GENE_ASSOCIATION: 64834.1</p>

<p>SUBSYSTEM: Fatty acid oxidation</p>

<p>EC Number: </p>

<p>Confidence Level: 0</p>

<p>AUTHORS: PMID:10970790</p>

<p>NOTES: </p>

</body>

</notes>

<listOfReactants>

<speciesReference species="M_arachdcoa_r"/>

<speciesReference species="M_h_r"/>

</listOfReactants>

<listOfProducts>

<speciesReference species="M_co2_r"/>

<speciesReference species="M_coa_r"/>

</listOfProducts>

<kineticLaw>

<math xmlns="<http://www.w3.org/1998/Math/MathML>">

<ci> FLUX_VALUE </ci>

</math>

<listOfParameters>

<parameter id="LOWER_BOUND" units="mmol_per_gDW_per_hr" value="0"/>

<parameter id="UPPER_BOUND" units="mmol_per_gDW_per_hr" value="1000"/>

<parameter id="FLUX_VALUE" units="mmol_per_gDW_per_hr" value="0"/>

<parameter id="OBJECTIVE_COEFFICIENT" units="dimensionless" value="0"/>

</listOfParameters>

</kineticLaw>

</reaction>

<reaction name="RE3289" id="R_RE3289R" metaid="_metaR_RE3289R" reversible="false" sboTerm="SBO:0000176">

<notes>

<body xmlns="<http://www.w3.org/1999/xhtml>">

<p>GENE_ASSOCIATION: (1543.1) or (1544.1) or (1571.1) or (1576.1) or (1558.1) or (1565.1) or (1548.1) or (1588.1) or (1559.1) or (1580.1) or (1555.1) or (1577.1) or (1549.1) or (1551.1) or (1572.1) or (1562.1) or (1573.1) or (11283.1) or (1545.1) or (1553.1) or (199974.1) or (260293.1) or (29785.1) or (64816.1) or (57834.1) or (66002.1)</p>

<p>SUBSYSTEM: Arachidonic acid metabolism</p>

<p>EC Number: 1.14.14.1</p>

<p>Confidence Level: 0</p>

<p>AUTHORS: PMID:8651708</p>

<p>NOTES: </p>

</body>

</notes>

<listOfReactants>

<speciesReference species="M_o2_r"/>

<speciesReference species="M_arachd_r"/>

</listOfReactants>

<listOfProducts>

<speciesReference species="M_C14771_r"/>

<speciesReference species="M_h2o_r"/>

</listOfProducts>

<kineticLaw>

<math xmlns="<http://www.w3.org/1998/Math/MathML>">

<ci> FLUX_VALUE </ci>

</math>

<listOfParameters>

<parameter id="LOWER_BOUND" units="mmol_per_gDW_per_hr" value="0"/>

<parameter id="UPPER_BOUND" units="mmol_per_gDW_per_hr" value="1000"/>

<parameter id="FLUX_VALUE" units="mmol_per_gDW_per_hr" value="0"/>

<parameter id="OBJECTIVE_COEFFICIENT" units="dimensionless" value="0"/>

</listOfParameters>

</kineticLaw>

</reaction>

<reaction name="RE3288" id="R_RE3288R" metaid="_metaR_RE3288R" reversible="false" sboTerm="SBO:0000176">

<notes>

<body xmlns="<http://www.w3.org/1999/xhtml>">

<p>GENE_ASSOCIATION: (1543.1) or (1544.1) or (1571.1) or (1576.1) or (1558.1) or (1565.1) or (1548.1) or (1588.1) or (1559.1) or (1580.1) or (1555.1) or (1577.1) or (1549.1) or (1551.1) or (1572.1) or (1562.1) or (1573.1) or (11283.1) or (1545.1) or (1553.1) or (199974.1) or (260293.1) or (29785.1) or (64816.1) or (57834.1) or (66002.1)</p>

<p>SUBSYSTEM: Arachidonic acid metabolism</p>

<p>EC Number: 1.14.14.1</p>

<p>Confidence Level: 0</p>

<p>AUTHORS: PMID:8651708</p>

<p>NOTES: </p>

</body>

</notes>

<listOfReactants>

<speciesReference species="M_o2_r"/>

<speciesReference species="M_arachd_r"/>

</listOfReactants>

<listOfProducts>

<speciesReference species="M_C14769_r"/>

<speciesReference species="M_h2o_r"/>

</listOfProducts>

<kineticLaw>

<math xmlns="<http://www.w3.org/1998/Math/MathML>">

<ci> FLUX_VALUE </ci>

</math>

<listOfParameters>

<parameter id="LOWER_BOUND" units="mmol_per_gDW_per_hr" value="0"/>

<parameter id="UPPER_BOUND" units="mmol_per_gDW_per_hr" value="1000"/>

<parameter id="FLUX_VALUE" units="mmol_per_gDW_per_hr" value="0"/>

<parameter id="OBJECTIVE_COEFFICIENT" units="dimensionless" value="0"/>

</listOfParameters>

</kineticLaw>

</reaction>

<reaction name="RE3287" id="R_RE3287R" metaid="_metaR_RE3287R" reversible="false" sboTerm="SBO:0000176">

<notes>

<body xmlns="<http://www.w3.org/1999/xhtml>">

<p>GENE_ASSOCIATION: (1543.1) or (1544.1) or (1571.1) or (1576.1) or (1558.1) or (1565.1) or (1548.1) or (1588.1) or (1559.1) or (1580.1) or (1555.1) or (1577.1) or (1549.1) or (1551.1) or (1572.1) or (1562.1) or (1573.1) or (11283.1) or (1545.1) or (1553.1) or (199974.1) or (260293.1) or (29785.1) or (64816.1) or (57834.1) or (66002.1)</p>

<p>SUBSYSTEM: Arachidonic acid metabolism</p>

<p>EC Number: 1.14.14.1</p>

<p>Confidence Level: 0</p>

<p>AUTHORS: PMID:8651708</p>

<p>NOTES: </p>

</body>

</notes>

<listOfReactants>

<speciesReference species="M_arachd_r"/>

<speciesReference species="M_o2_r"/>

</listOfReactants>

<listOfProducts>

<speciesReference species="M_C14770_r"/>

<speciesReference species="M_h2o_r"/>

</listOfProducts>

<kineticLaw>

<math xmlns="<http://www.w3.org/1998/Math/MathML>">

<ci> FLUX_VALUE </ci>

</math>

<listOfParameters>

<parameter id="LOWER_BOUND" units="mmol_per_gDW_per_hr" value="0"/>

<parameter id="UPPER_BOUND" units="mmol_per_gDW_per_hr" value="1000"/>

<parameter id="FLUX_VALUE" units="mmol_per_gDW_per_hr" value="0"/>

<parameter id="OBJECTIVE_COEFFICIENT" units="dimensionless" value="0"/>

</listOfParameters>

</kineticLaw>

</reaction>

<reaction name="RE3286" id="R_RE3286R" metaid="_metaR_RE3286R" reversible="false" sboTerm="SBO:0000176">

<notes>

<body xmlns="<http://www.w3.org/1999/xhtml>">

<p>GENE_ASSOCIATION: (1543.1) or (1544.1) or (1571.1) or (1576.1) or (1558.1) or (1565.1) or (1548.1) or (1588.1) or (1559.1) or (1580.1) or (1555.1) or (1577.1) or (1549.1) or (1551.1) or (1572.1) or (1562.1) or (1573.1) or (11283.1) or (1545.1) or (1553.1) or (199974.1) or (260293.1) or (29785.1) or (64816.1) or (57834.1) or (66002.1)</p>

<p>SUBSYSTEM: Arachidonic acid metabolism</p>

<p>EC Number: 1.14.14.1</p>

<p>Confidence Level: 0</p>

<p>AUTHORS: PMID:8651708</p>

<p>NOTES: </p>

</body>

</notes>

<listOfReactants>

<speciesReference species="M_o2_r"/>

<speciesReference species="M_arachd_r"/>

</listOfReactants>

<listOfProducts>

<speciesReference species="M_C14768_r"/>

<speciesReference species="M_h2o_r"/>

</listOfProducts>

<kineticLaw>

<math xmlns="<http://www.w3.org/1998/Math/MathML>">

<ci> FLUX_VALUE </ci>

</math>

<listOfParameters>

<parameter id="LOWER_BOUND" units="mmol_per_gDW_per_hr" value="0"/>

<parameter id="UPPER_BOUND" units="mmol_per_gDW_per_hr" value="1000"/>

<parameter id="FLUX_VALUE" units="mmol_per_gDW_per_hr" value="0"/>

<parameter id="OBJECTIVE_COEFFICIENT" units="dimensionless" value="0"/>

</listOfParameters>

</kineticLaw>

</reaction>

<reaction name="fatty acid electroneutral transport" id="R_FATP5t" metaid="_metaR_FATP5t" reversible="true" sboTerm="SBO:0000185">

<notes>

<body xmlns="<http://www.w3.org/1999/xhtml>">

<p>GENE_ASSOCIATION: 10998.1</p>

<p>SUBSYSTEM: Transport, extracellular</p>

<p>EC Number: </p>

<p>Confidence Level: 4</p>

<p>AUTHORS: PMID:10479480,PMID:12856180,PMID:15522816</p>

<p>NOTES: electroneutral transport NJ</p>

</body>

</notes>

<listOfReactants>

<speciesReference species="M_arachd_c"/>

<speciesReference species="M_na1_c"/>

</listOfReactants>

<listOfProducts>

<speciesReference species="M_arachd_e"/>

<speciesReference species="M_na1_e"/>

</listOfProducts>

<kineticLaw>

<math xmlns="<http://www.w3.org/1998/Math/MathML>">

<ci> FLUX_VALUE </ci>

</math>

<listOfParameters>

<parameter id="LOWER_BOUND" units="mmol_per_gDW_per_hr" value="-1000"/>

<parameter id="UPPER_BOUND" units="mmol_per_gDW_per_hr" value="1000"/>

<parameter id="FLUX_VALUE" units="mmol_per_gDW_per_hr" value="0"/>

<parameter id="OBJECTIVE_COEFFICIENT" units="dimensionless" value="0"/>

</listOfParameters>

</kineticLaw>

</reaction>

<reaction name="fatty acid transport via diffusion" id="R_ARACHDt2" metaid="_metaR_ARACHDt2" reversible="true" sboTerm="SBO:0000185">

<notes>

<body xmlns="<http://www.w3.org/1999/xhtml>">

<p>GENE_ASSOCIATION: </p>

<p>SUBSYSTEM: Transport, extracellular</p>

<p>EC Number: </p>

<p>Confidence Level: 2</p>

<p>AUTHORS: PMID:11788354,PMID:12864740</p>

<p>NOTES: NJ</p>

</body>

</notes>

<listOfReactants>

<speciesReference species="M_arachd_e"/>

</listOfReactants>

<listOfProducts>

<speciesReference species="M_arachd_c"/>

</listOfProducts>

<kineticLaw>

<math xmlns="<http://www.w3.org/1998/Math/MathML>">

<ci> FLUX_VALUE </ci>

</math>

<listOfParameters>

<parameter id="LOWER_BOUND" units="mmol_per_gDW_per_hr" value="-1000"/>

<parameter id="UPPER_BOUND" units="mmol_per_gDW_per_hr" value="1000"/>

<parameter id="FLUX_VALUE" units="mmol_per_gDW_per_hr" value="0"/>

<parameter id="OBJECTIVE_COEFFICIENT" units="dimensionless" value="0"/>

</listOfParameters>

</kineticLaw>

</reaction>

<reaction name="RE3289" id="R_RE3289C" metaid="_metaR_RE3289C" reversible="false" sboTerm="SBO:0000176">

<notes>

<body xmlns="<http://www.w3.org/1999/xhtml>">

<p>GENE_ASSOCIATION: </p>

<p>SUBSYSTEM: Arachidonic acid metabolism</p>

<p>EC Number: 1.14.14.1</p>

<p>Confidence Level: 0</p>

<p>AUTHORS: PMID:8651708</p>

<p>NOTES: </p>

</body>

</notes>

<listOfReactants>

<speciesReference species="M_arachd_c"/>

<speciesReference species="M_o2_c"/>

<speciesReference species="M_fadh2_c"/>

</listOfReactants>

<listOfProducts>

<speciesReference species="M_C14771_c"/>

<speciesReference species="M_h2o_c"/>

<speciesReference species="M_fad_c"/>

</listOfProducts>

<kineticLaw>

<math xmlns="<http://www.w3.org/1998/Math/MathML>">

<ci> FLUX_VALUE </ci>

</math>

<listOfParameters>

<parameter id="LOWER_BOUND" units="mmol_per_gDW_per_hr" value="0"/>

<parameter id="UPPER_BOUND" units="mmol_per_gDW_per_hr" value="1000"/>

<parameter id="FLUX_VALUE" units="mmol_per_gDW_per_hr" value="0"/>

<parameter id="OBJECTIVE_COEFFICIENT" units="dimensionless" value="0"/>

</listOfParameters>

</kineticLaw>

</reaction>

<reaction name="RE3288" id="R_RE3288C" metaid="_metaR_RE3288C" reversible="false" sboTerm="SBO:0000176">

<notes>

<body xmlns="<http://www.w3.org/1999/xhtml>">

<p>GENE_ASSOCIATION: </p>

<p>SUBSYSTEM: Arachidonic acid metabolism</p>

<p>EC Number: 1.14.14.1</p>

<p>Confidence Level: 0</p>

<p>AUTHORS: PMID:8651708</p>

<p>NOTES: </p>

</body>

</notes>

<listOfReactants>

<speciesReference species="M_arachd_c"/>

<speciesReference species="M_o2_c"/>

<speciesReference species="M_fadh2_c"/>

</listOfReactants>

<listOfProducts>

<speciesReference species="M_C14769_c"/>

<speciesReference species="M_h2o_c"/>

<speciesReference species="M_fad_c"/>

</listOfProducts>

<kineticLaw>

<math xmlns="<http://www.w3.org/1998/Math/MathML>">

<ci> FLUX_VALUE </ci>

</math>

<listOfParameters>

<parameter id="LOWER_BOUND" units="mmol_per_gDW_per_hr" value="0"/>

<parameter id="UPPER_BOUND" units="mmol_per_gDW_per_hr" value="1000"/>

<parameter id="FLUX_VALUE" units="mmol_per_gDW_per_hr" value="0"/>

<parameter id="OBJECTIVE_COEFFICIENT" units="dimensionless" value="0"/>

</listOfParameters>

</kineticLaw>

</reaction>

<reaction name="RE3287" id="R_RE3287C" metaid="_metaR_RE3287C" reversible="false" sboTerm="SBO:0000176">

<notes>

<body xmlns="<http://www.w3.org/1999/xhtml>">

<p>GENE_ASSOCIATION: </p>

<p>SUBSYSTEM: Arachidonic acid metabolism</p>

<p>EC Number: 1.14.14.1</p>

<p>Confidence Level: 0</p>

<p>AUTHORS: PMID:8651708</p>

<p>NOTES: </p>

</body>

</notes>

<listOfReactants>

<speciesReference species="M_arachd_c"/>

<speciesReference species="M_o2_c"/>

<speciesReference species="M_fadh2_c"/>

</listOfReactants>

<listOfProducts>

<speciesReference species="M_h2o_c"/>

<speciesReference species="M_C14770_c"/>

<speciesReference species="M_fad_c"/>

</listOfProducts>

<kineticLaw>

<math xmlns="<http://www.w3.org/1998/Math/MathML>">

<ci> FLUX_VALUE </ci>

</math>

<listOfParameters>

<parameter id="LOWER_BOUND" units="mmol_per_gDW_per_hr" value="0"/>

<parameter id="UPPER_BOUND" units="mmol_per_gDW_per_hr" value="1000"/>

<parameter id="FLUX_VALUE" units="mmol_per_gDW_per_hr" value="0"/>

<parameter id="OBJECTIVE_COEFFICIENT" units="dimensionless" value="0"/>

</listOfParameters>

</kineticLaw>

</reaction>

<reaction name="RE3286" id="R_RE3286C" metaid="_metaR_RE3286C" reversible="false" sboTerm="SBO:0000176">

<notes>

<body xmlns="<http://www.w3.org/1999/xhtml>">

<p>GENE_ASSOCIATION: </p>

<p>SUBSYSTEM: Arachidonic acid metabolism</p>

<p>EC Number: 1.14.14.1</p>

<p>Confidence Level: 0</p>

<p>AUTHORS: PMID:8651708</p>

<p>NOTES: </p>

</body>

</notes>

<listOfReactants>

<speciesReference species="M_arachd_c"/>

<speciesReference species="M_o2_c"/>

<speciesReference species="M_fadh2_c"/>

</listOfReactants>

<listOfProducts>

<speciesReference species="M_C14768_c"/>

<speciesReference species="M_h2o_c"/>

<speciesReference species="M_fad_c"/>

</listOfProducts>

<kineticLaw>

<math xmlns="<http://www.w3.org/1998/Math/MathML>">

<ci> FLUX_VALUE </ci>

</math>

<listOfParameters>

<parameter id="LOWER_BOUND" units="mmol_per_gDW_per_hr" value="0"/>

<parameter id="UPPER_BOUND" units="mmol_per_gDW_per_hr" value="1000"/>

<parameter id="FLUX_VALUE" units="mmol_per_gDW_per_hr" value="0"/>

<parameter id="OBJECTIVE_COEFFICIENT" units="dimensionless" value="0"/>

</listOfParameters>

</kineticLaw>

</reaction>

<reaction name="Resistance-Nodulation-Cell Division (RND) TCDB:2.A.60.1.2" id="R_r2313" metaid="_metaR_r2313" reversible="true" sboTerm="SBO:0000185">

<notes>

<body xmlns="<http://www.w3.org/1999/xhtml>">

<p>GENE_ASSOCIATION: </p>

<p>SUBSYSTEM: Transport, extracellular</p>

<p>EC Number: </p>

<p>Confidence Level: 0</p>

<p>AUTHORS: PMID:11076394,PMID:11076396,PMID:14579113</p>

<p>NOTES: </p>

</body>

</notes>

<listOfReactants>

<speciesReference species="M_arachd_e"/>

<speciesReference species="M_hco3_c"/>

</listOfReactants>

<listOfProducts>

<speciesReference species="M_arachd_c"/>

<speciesReference species="M_hco3_e"/>

</listOfProducts>

<kineticLaw>

<math xmlns="<http://www.w3.org/1998/Math/MathML>">

<ci> FLUX_VALUE </ci>

</math>

<listOfParameters>

<parameter id="LOWER_BOUND" units="mmol_per_gDW_per_hr" value="-1000"/>

<parameter id="UPPER_BOUND" units="mmol_per_gDW_per_hr" value="1000"/>

<parameter id="FLUX_VALUE" units="mmol_per_gDW_per_hr" value="0"/>

<parameter id="OBJECTIVE_COEFFICIENT" units="dimensionless" value="0"/>

</listOfParameters>

</kineticLaw>

</reaction>

<reaction name="Resistance-Nodulation-Cell Division (RND) TCDB:2.A.60.1.5" id="R_r2218" metaid="_metaR_r2218" reversible="true" sboTerm="SBO:0000185">

<notes>

<body xmlns="<http://www.w3.org/1999/xhtml>">

<p>GENE_ASSOCIATION: 6566.1</p>

<p>SUBSYSTEM: Transport, extracellular</p>

<p>EC Number: </p>

<p>Confidence Level: 0</p>

<p>AUTHORS: PMID:11076396,PMID:14579113</p>

<p>NOTES: </p>

</body>

</notes>

<listOfReactants>

<speciesReference species="M_arachd_e"/>

<speciesReference species="M_hco3_c"/>

</listOfReactants>

<listOfProducts>

<speciesReference species="M_arachd_c"/>

<speciesReference species="M_hco3_e"/>

<speciesReference species="M_HC02199_e"/>

</listOfProducts>

<kineticLaw>

<math xmlns="<http://www.w3.org/1998/Math/MathML>">

<ci> FLUX_VALUE </ci>

</math>

<listOfParameters>

<parameter id="LOWER_BOUND" units="mmol_per_gDW_per_hr" value="-1000"/>

<parameter id="UPPER_BOUND" units="mmol_per_gDW_per_hr" value="1000"/>

<parameter id="FLUX_VALUE" units="mmol_per_gDW_per_hr" value="0"/>

<parameter id="OBJECTIVE_COEFFICIENT" units="dimensionless" value="0"/>

</listOfParameters>

</kineticLaw>

</reaction>

<reaction name="Resistance-Nodulation-Cell Division (RND) TCDB:2.A.60.1.5" id="R_r2219" metaid="_metaR_r2219" reversible="true" sboTerm="SBO:0000185">

<notes>

<body xmlns="<http://www.w3.org/1999/xhtml>">

<p>GENE_ASSOCIATION: 6566.1</p>

<p>SUBSYSTEM: Transport, extracellular</p>

<p>EC Number: </p>

<p>Confidence Level: 0</p>

<p>AUTHORS: PMID:11076396,PMID:14579113</p>

<p>NOTES: </p>

</body>

</notes>

<listOfReactants>

<speciesReference species="M_arachd_e"/>

<speciesReference species="M_hco3_c"/>

</listOfReactants>

<listOfProducts>

<speciesReference species="M_arachd_c"/>

<speciesReference species="M_hco3_e"/>

<speciesReference species="M_HC02200_e"/>

</listOfProducts>

<kineticLaw>

<math xmlns="<http://www.w3.org/1998/Math/MathML>">

<ci> FLUX_VALUE </ci>

</math>

<listOfParameters>

<parameter id="LOWER_BOUND" units="mmol_per_gDW_per_hr" value="-1000"/>

<parameter id="UPPER_BOUND" units="mmol_per_gDW_per_hr" value="1000"/>

<parameter id="FLUX_VALUE" units="mmol_per_gDW_per_hr" value="0"/>

<parameter id="OBJECTIVE_COEFFICIENT" units="dimensionless" value="0"/>

</listOfParameters>

</kineticLaw>

</reaction>

<reaction name="Resistance-Nodulation-Cell Division (RND) TCDB:2.A.60.1.5" id="R_r2220" metaid="_metaR_r2220" reversible="true" sboTerm="SBO:0000185">

<notes>

<body xmlns="<http://www.w3.org/1999/xhtml>">

<p>GENE_ASSOCIATION: </p>

<p>SUBSYSTEM: Transport, extracellular</p>

<p>EC Number: </p>

<p>Confidence Level: 0</p>

<p>AUTHORS: PMID:11076396,PMID:14579113</p>

<p>NOTES: </p>

</body>

</notes>

<listOfReactants>

<speciesReference species="M_arachd_e"/>

<speciesReference species="M_hco3_c"/>

</listOfReactants>

<listOfProducts>

<speciesReference species="M_arachd_c"/>

<speciesReference species="M_hco3_e"/>

<speciesReference species="M_HC02201_e"/>

</listOfProducts>

<kineticLaw>

<math xmlns="<http://www.w3.org/1998/Math/MathML>">

<ci> FLUX_VALUE </ci>

</math>

<listOfParameters>

<parameter id="LOWER_BOUND" units="mmol_per_gDW_per_hr" value="-1000"/>

<parameter id="UPPER_BOUND" units="mmol_per_gDW_per_hr" value="1000"/>

<parameter id="FLUX_VALUE" units="mmol_per_gDW_per_hr" value="0"/>

<parameter id="OBJECTIVE_COEFFICIENT" units="dimensionless" value="0"/>

</listOfParameters>

</kineticLaw>

</reaction>

<reaction name="carbamoyl phosphate transport, endoplasmic reticulum" id="R_CBPter" metaid="_metaR_CBPter" reversible="true" sboTerm="SBO:0000185">

<notes>

<body xmlns="<http://www.w3.org/1999/xhtml>">

<p>GENE_ASSOCIATION: </p>

<p>SUBSYSTEM: Transport, endoplasmic reticular</p>

<p>EC Number: </p>

<p>Confidence Level: 2</p>

<p>AUTHORS: PMID:12192101,PMID:8384451</p>

<p>NOTES: NCD</p>

</body>

</notes>

<listOfReactants>

<speciesReference species="M_ppi_c"/>

<speciesReference species="M_pi_c"/>

</listOfReactants>

<listOfProducts>

<speciesReference species="M_cbp_r"/>

<speciesReference species="M_pi_r"/>

</listOfProducts>

<kineticLaw>

<math xmlns="<http://www.w3.org/1998/Math/MathML>">

<ci> FLUX_VALUE </ci>

</math>

<listOfParameters>

<parameter id="LOWER_BOUND" units="mmol_per_gDW_per_hr" value="-1000"/>

<parameter id="UPPER_BOUND" units="mmol_per_gDW_per_hr" value="1000"/>

<parameter id="FLUX_VALUE" units="mmol_per_gDW_per_hr" value="0"/>

<parameter id="OBJECTIVE_COEFFICIENT" units="dimensionless" value="0"/>

</listOfParameters>

</kineticLaw>

</reaction>

<reaction name="fatty-acid--CoA ligase" id="R_FACOAL204" metaid="_metaR_FACOAL204" reversible="true" sboTerm="SBO:0000176">

<notes>

<body xmlns="<http://www.w3.org/1999/xhtml>">

<p>GENE_ASSOCIATION: (22305.1) or (2181.2) or (2181.1) or (2180.1) or (22305.2) or (2182.1) or (2182.2)</p>

<p>SUBSYSTEM: Fatty acid oxidation</p>

<p>EC Number: 6.2.1.3</p>

<p>Confidence Level: 4</p>

<p>AUTHORS: PMID:15292367</p>

<p>NOTES: rev version so additional rxn doesn&amp;apos;t need to be added to &amp;quot;unload&amp;quot; AA NJ. Merged GPR from FACOAL204 and FACOAL204i - (May 2011) IT</p>

</body>

</notes>

<listOfReactants>

<speciesReference species="M_arachd_c"/>

<speciesReference species="M_atp_c"/>

<speciesReference species="M_coa_c"/>

</listOfReactants>

<listOfProducts>

<speciesReference species="M_ppi_c"/>

<speciesReference species="M_arachdcoa_c"/>

<speciesReference species="M_amp_c"/>

</listOfProducts>

<kineticLaw>

<math xmlns="<http://www.w3.org/1998/Math/MathML>">

<ci> FLUX_VALUE </ci>

</math>

<listOfParameters>

<parameter id="LOWER_BOUND" units="mmol_per_gDW_per_hr" value="-1000"/>

<parameter id="UPPER_BOUND" units="mmol_per_gDW_per_hr" value="1000"/>

<parameter id="FLUX_VALUE" units="mmol_per_gDW_per_hr" value="0"/>

<parameter id="OBJECTIVE_COEFFICIENT" units="dimensionless" value="0"/>

</listOfParameters>

</kineticLaw>

</reaction>

<reaction name="Carbamoyl phosphate phosphotransferase, endoplasmic reticulum" id="R_CBPPer" metaid="_metaR_CBPPer" reversible="false" sboTerm="SBO:0000176">

<notes>

<body xmlns="<http://www.w3.org/1999/xhtml>">

<p>GENE_ASSOCIATION: (2538.1) or (57818.1) or (92579.1)</p>

<p>SUBSYSTEM: Glycolysis/gluconeogenesis</p>

<p>EC Number: </p>

<p>Confidence Level: 2</p>

<p>AUTHORS: PMID:11946414,PMID:8384451</p>

<p>NOTES: See PMID: 11946414 NCD</p>

</body>

</notes>

<listOfReactants>

<speciesReference species="M_cbp_r"/>

<speciesReference species="M_h_r"/>

</listOfReactants>

<listOfProducts>

<speciesReference species="M_nh4_r"/>

<speciesReference species="M_co2_r"/>

</listOfProducts>

<kineticLaw>

<math xmlns="<http://www.w3.org/1998/Math/MathML>">

<ci> FLUX_VALUE </ci>

</math>

<listOfParameters>

<parameter id="LOWER_BOUND" units="mmol_per_gDW_per_hr" value="0"/>

<parameter id="UPPER_BOUND" units="mmol_per_gDW_per_hr" value="1000"/>

<parameter id="FLUX_VALUE" units="mmol_per_gDW_per_hr" value="0"/>

<parameter id="OBJECTIVE_COEFFICIENT" units="dimensionless" value="0"/>

</listOfParameters>

</kineticLaw>

</reaction>

<reaction name="orotate phosphoribosyltransferase" id="R_ORPT" metaid="_metaR_ORPT" reversible="true" sboTerm="SBO:0000176">

<notes>

<body xmlns="<http://www.w3.org/1999/xhtml>">

<p>GENE_ASSOCIATION: 7372.1</p>

<p>SUBSYSTEM: Pyrimidine synthesis</p>

<p>EC Number: 2.4.2.10</p>

<p>Confidence Level: 4</p>

<p>AUTHORS: PMID:3279416,PMID:6574608,PMID:6893554,PMID:9042911</p>

<p>NOTES: </p>

</body>

</notes>

<listOfReactants>

<speciesReference species="M_ppi_c"/>

</listOfReactants>

<listOfProducts>

<speciesReference species="M_orot_c"/>

</listOfProducts>

<kineticLaw>

<math xmlns="<http://www.w3.org/1998/Math/MathML>">

<ci> FLUX_VALUE </ci>

</math>

<listOfParameters>

<parameter id="LOWER_BOUND" units="mmol_per_gDW_per_hr" value="-1000"/>

<parameter id="UPPER_BOUND" units="mmol_per_gDW_per_hr" value="1000"/>

<parameter id="FLUX_VALUE" units="mmol_per_gDW_per_hr" value="0"/>

<parameter id="OBJECTIVE_COEFFICIENT" units="dimensionless" value="0"/>

</listOfParameters>

</kineticLaw>

</reaction>

<reaction name="R group artificial flux" id="R_ARTFR206" metaid="_metaR_ARTFR206" reversible="false" sboTerm="SBO:0000185">

<notes>

<body xmlns="<http://www.w3.org/1999/xhtml>">

<p>GENE_ASSOCIATION: </p>

<p>SUBSYSTEM: R group synthesis</p>

<p>EC Number: </p>

<p>Confidence Level: 0</p>

<p>AUTHORS: </p>

<p>NOTES: </p>

</body>

</notes>

<listOfReactants>

<speciesReference species="M_h_m" stoichiometry="2"/>

<speciesReference species="M_arachdcoa_c"/>

<speciesReference species="M_nadph_m" stoichiometry="2"/>

</listOfReactants>

<listOfProducts>

<speciesReference species="M_R2coa_hs_c" stoichiometry="1.25"/>

<speciesReference species="M_nadp_m" stoichiometry="2"/>

<speciesReference species="M_fad_m" stoichiometry="2"/>

</listOfProducts>

<kineticLaw>

<math xmlns="<http://www.w3.org/1998/Math/MathML>">

<ci> FLUX_VALUE </ci>

</math>

<listOfParameters>

<parameter id="LOWER_BOUND" units="mmol_per_gDW_per_hr" value="0"/>

<parameter id="UPPER_BOUND" units="mmol_per_gDW_per_hr" value="1000"/>

<parameter id="FLUX_VALUE" units="mmol_per_gDW_per_hr" value="0"/>

<parameter id="OBJECTIVE_COEFFICIENT" units="dimensionless" value="0"/>

</listOfParameters>

</kineticLaw>

</reaction>

<reaction name="sterol O-acyltransferase (acyl-Coenzyme A: cholesterol acyltransferase) 1" id="R_SOAT12" metaid="_metaR_SOAT12" reversible="false" sboTerm="SBO:0000176">

<notes>

<body xmlns="<http://www.w3.org/1999/xhtml>">

<p>GENE_ASSOCIATION: 6646.1</p>

<p>SUBSYSTEM: Cholesterol metabolism</p>

<p>EC Number: </p>

<p>Confidence Level: 4</p>

<p>AUTHORS: PMID:15850387,PMID:8407899</p>

<p>NOTES: SOAT11 and SOAT12 are templates, customized rxns can be formed particular ester moieties. NJ</p>

</body>

</notes>

<listOfReactants>

<speciesReference species="M_R2coa_hs_c"/>

</listOfReactants>

<listOfProducts>

<speciesReference species="M_xolest_hs_c"/>

<speciesReference species="M_coa_c"/>

</listOfProducts>

<kineticLaw>

<math xmlns="<http://www.w3.org/1998/Math/MathML>">

<ci> FLUX_VALUE </ci>

</math>

<listOfParameters>

<parameter id="LOWER_BOUND" units="mmol_per_gDW_per_hr" value="0"/>

<parameter id="UPPER_BOUND" units="mmol_per_gDW_per_hr" value="1000"/>

<parameter id="FLUX_VALUE" units="mmol_per_gDW_per_hr" value="0"/>

<parameter id="OBJECTIVE_COEFFICIENT" units="dimensionless" value="0"/>

</listOfParameters>

</kineticLaw>

</reaction>

<reaction name="fatty acid beta oxidation(C9br-->C7br)m" id="R_FAOXC9BRC7BRm" metaid="_metaR_FAOXC9BRC7BRm" reversible="false" sboTerm="SBO:0000176">

<notes>

<body xmlns="<http://www.w3.org/1999/xhtml>">

<p>SUBSYSTEM: Fatty acid oxidation</p>

<p>Confidence Level: 4</p>

<p>AUTHORS: PMID: 9469587,HMDB06258,PMID:9714723,PMID:11591435</p>

<p>EC Number: </p>

<p>NOTES: First cycle of mitochondrial beta oxidation. The mitochondrial acylcoA dehydrogenases cannot act on branched chain fatty acids so no GPR association could be found for this reaction. Recon 1 mentions ACADM gene with this reaction. In Recon 1, the neutral formula for dmnoncoa should be corrected to C32H56N7O17P3S (HMDB11604) and charged formula to C32H52N7O17P3S with -4 as the charge. Therefore the reaction appears unbalanced here.</p>

<p>GENE_ASSOCIATION: </p>

</body>

</notes>

<listOfReactants>

<speciesReference species="M_fad_m"/>

<speciesReference species="M_h2o_m"/>

<speciesReference species="M_coa_m"/>

<speciesReference species="M_nad_m"/>

</listOfReactants>

<listOfProducts>

<speciesReference species="M_dmhepcoa_m"/>

<speciesReference species="M_h_m"/>

<speciesReference species="M_nadh_m"/>

</listOfProducts>

<kineticLaw>

<math xmlns="<http://www.w3.org/1998/Math/MathML>">

<ci> FLUX_VALUE </ci>

</math>

<listOfParameters>

<parameter id="LOWER_BOUND" units="mmol_per_gDW_per_hr" value="0"/>

<parameter id="UPPER_BOUND" units="mmol_per_gDW_per_hr" value="1000"/>

<parameter id="FLUX_VALUE" units="mmol_per_gDW_per_hr" value="0"/>

<parameter id="OBJECTIVE_COEFFICIENT" units="dimensionless" value="0"/>

</listOfParameters>

</kineticLaw>

</reaction>

<reaction name="fatty-acyl-CoA elongation (n-C20:4CoA)" id="R_FAEL204" metaid="_metaR_FAEL204" reversible="false" sboTerm="SBO:0000176">

<notes>

<body xmlns="<http://www.w3.org/1999/xhtml>">

<p>GENE_ASSOCIATION: 6785.1</p>

<p>SUBSYSTEM: Fatty acid synthesis</p>

<p>EC Number: 2.3.1.86</p>

<p>Confidence Level: 4</p>

<p>AUTHORS: PMID:11138005,PMID:15189125</p>

<p>NOTES: fatty acid elongation step NJ</p>

</body>

</notes>

<listOfReactants>

<speciesReference species="M_arachdcoa_c"/>

<speciesReference species="M_o2_c"/>

<speciesReference species="M_h_c" stoichiometry="5"/>

<speciesReference species="M_nadph_c" stoichiometry="4"/>

</listOfReactants>

<listOfProducts>

<speciesReference species="M_h2o_c" stoichiometry="3"/>

<speciesReference species="M_co2_c"/>

<speciesReference species="M_nadp_c" stoichiometry="4"/>

<speciesReference species="M_coa_c"/>

</listOfProducts>

<kineticLaw>

<math xmlns="<http://www.w3.org/1998/Math/MathML>">

<ci> FLUX_VALUE </ci>

</math>

<listOfParameters>

<parameter id="LOWER_BOUND" units="mmol_per_gDW_per_hr" value="0"/>

<parameter id="UPPER_BOUND" units="mmol_per_gDW_per_hr" value="1000"/>

<parameter id="FLUX_VALUE" units="mmol_per_gDW_per_hr" value="0"/>

<parameter id="OBJECTIVE_COEFFICIENT" units="dimensionless" value="0"/>

</listOfParameters>

</kineticLaw>

</reaction>

<reaction name="CO2 endoplasmic reticular transport via diffusion" id="R_CO2ter" metaid="_metaR_CO2ter" reversible="true" sboTerm="SBO:0000185">

<notes>

<body xmlns="<http://www.w3.org/1999/xhtml>">

<p>GENE_ASSOCIATION: </p>

<p>SUBSYSTEM: Transport, endoplasmic reticular</p>

<p>EC Number: </p>

<p>Confidence Level: 1</p>

<p>AUTHORS: </p>

<p>NOTES: NCD</p>

</body>

</notes>

<listOfReactants>

<speciesReference species="M_co2_c"/>

</listOfReactants>

<listOfProducts>

<speciesReference species="M_co2_r"/>

</listOfProducts>

<kineticLaw>

<math xmlns="<http://www.w3.org/1998/Math/MathML>">

<ci> FLUX_VALUE </ci>

</math>

<listOfParameters>

<parameter id="LOWER_BOUND" units="mmol_per_gDW_per_hr" value="-1000"/>

<parameter id="UPPER_BOUND" units="mmol_per_gDW_per_hr" value="1000"/>

<parameter id="FLUX_VALUE" units="mmol_per_gDW_per_hr" value="0"/>

<parameter id="OBJECTIVE_COEFFICIENT" units="dimensionless" value="0"/>

</listOfParameters>

</kineticLaw>

</reaction>

<reaction name="CO2 Golgi transport" id="R_CO2tg" metaid="_metaR_CO2tg" reversible="true" sboTerm="SBO:0000185">

<notes>

<body xmlns="<http://www.w3.org/1999/xhtml>">

<p>GENE_ASSOCIATION: </p>

<p>SUBSYSTEM: Transport, golgi apparatus</p>

<p>EC Number: </p>

<p>Confidence Level: 1</p>

<p>AUTHORS: </p>

<p>NOTES: NCD</p>

</body>

</notes>

<listOfReactants>

<speciesReference species="M_co2_c"/>

</listOfReactants>

<listOfProducts>

<speciesReference species="M_co2_g"/>

</listOfProducts>

<kineticLaw>

<math xmlns="<http://www.w3.org/1998/Math/MathML>">

<ci> FLUX_VALUE </ci>

</math>

<listOfParameters>

<parameter id="LOWER_BOUND" units="mmol_per_gDW_per_hr" value="-1000"/>

<parameter id="UPPER_BOUND" units="mmol_per_gDW_per_hr" value="1000"/>

<parameter id="FLUX_VALUE" units="mmol_per_gDW_per_hr" value="0"/>

<parameter id="OBJECTIVE_COEFFICIENT" units="dimensionless" value="0"/>

</listOfParameters>

</kineticLaw>

</reaction>

<reaction name="RE3181" id="R_RE3181C" metaid="_metaR_RE3181C" reversible="true" sboTerm="SBO:0000176">

<notes>

<body xmlns="<http://www.w3.org/1999/xhtml>">

<p>GENE_ASSOCIATION: </p>

<p>SUBSYSTEM: Cholesterol metabolism</p>

<p>EC Number: </p>

<p>Confidence Level: 0</p>

<p>AUTHORS: PMID:11111101</p>

<p>NOTES: </p>

</body>

</notes>

<listOfReactants>

<speciesReference species="M_co2_c"/>

</listOfReactants>

<listOfProducts>

<speciesReference species="M_h2o_c" stoichiometry="2"/>

<speciesReference species="M_44mctr_c" stoichiometry="3"/>

<speciesReference species="M_CE5049_c" stoichiometry="4"/>

</listOfProducts>

<kineticLaw>

<math xmlns="<http://www.w3.org/1998/Math/MathML>">

<ci> FLUX_VALUE </ci>

</math>

<listOfParameters>

<parameter id="LOWER_BOUND" units="mmol_per_gDW_per_hr" value="-1000"/>

<parameter id="UPPER_BOUND" units="mmol_per_gDW_per_hr" value="1000"/>

<parameter id="FLUX_VALUE" units="mmol_per_gDW_per_hr" value="0"/>

<parameter id="OBJECTIVE_COEFFICIENT" units="dimensionless" value="0"/>

</listOfParameters>

</kineticLaw>

</reaction>

<reaction name="RE3180" id="R_RE3180C" metaid="_metaR_RE3180C" reversible="true" sboTerm="SBO:0000176">

<notes>

<body xmlns="<http://www.w3.org/1999/xhtml>">

<p>GENE_ASSOCIATION: </p>

<p>SUBSYSTEM: Cholesterol metabolism</p>

<p>EC Number: </p>

<p>Confidence Level: 0</p>

<p>AUTHORS: PMID:11111101</p>

<p>NOTES: </p>

</body>

</notes>

<listOfReactants>

<speciesReference species="M_co2_c"/>

</listOfReactants>

<listOfProducts>

<speciesReference species="M_CE5049_c" stoichiometry="4"/>

<speciesReference species="M_h2o_c" stoichiometry="2"/>

</listOfProducts>

<kineticLaw>

<math xmlns="<http://www.w3.org/1998/Math/MathML>">

<ci> FLUX_VALUE </ci>

</math>

<listOfParameters>

<parameter id="LOWER_BOUND" units="mmol_per_gDW_per_hr" value="-1000"/>

<parameter id="UPPER_BOUND" units="mmol_per_gDW_per_hr" value="1000"/>

<parameter id="FLUX_VALUE" units="mmol_per_gDW_per_hr" value="0"/>

<parameter id="OBJECTIVE_COEFFICIENT" units="dimensionless" value="0"/>

</listOfParameters>

</kineticLaw>

</reaction>

<reaction name="RE2533" id="R_RE2533C" metaid="_metaR_RE2533C" reversible="true" sboTerm="SBO:0000176">

<notes>

<body xmlns="<http://www.w3.org/1999/xhtml>">

<p>GENE_ASSOCIATION: </p>

<p>SUBSYSTEM: Vitamin E metabolism</p>

<p>EC Number: </p>

<p>Confidence Level: 0</p>

<p>AUTHORS: PMID:9425126</p>

<p>NOTES: </p>

</body>

</notes>

<listOfReactants>

<speciesReference species="M_co2_c"/>

</listOfReactants>

<listOfProducts>

<speciesReference species="M_CE6000_c"/>

</listOfProducts>

<kineticLaw>

<math xmlns="<http://www.w3.org/1998/Math/MathML>">

<ci> FLUX_VALUE </ci>

</math>

<listOfParameters>

<parameter id="LOWER_BOUND" units="mmol_per_gDW_per_hr" value="-1000"/>

<parameter id="UPPER_BOUND" units="mmol_per_gDW_per_hr" value="1000"/>

<parameter id="FLUX_VALUE" units="mmol_per_gDW_per_hr" value="0"/>

<parameter id="OBJECTIVE_COEFFICIENT" units="dimensionless" value="0"/>

</listOfParameters>

</kineticLaw>

</reaction>

<reaction name="EC:2.3.1.15" id="R_r1190" metaid="_metaR_r1190" reversible="false" sboTerm="SBO:0000176">

<notes>

<body xmlns="<http://www.w3.org/1999/xhtml>">

<p>GENE_ASSOCIATION: </p>

<p>SUBSYSTEM: Unassigned</p>

<p>EC Number: 2.3.1.15</p>

<p>Confidence Level: 0</p>

<p>AUTHORS: PMID:12573444,PMID:17170135,PMID:17389595</p>

<p>NOTES: </p>

</body>

</notes>

<listOfReactants>

<speciesReference species="M_arachdcoa_c"/>

</listOfReactants>

<listOfProducts>

<speciesReference species="M_HC02036_c"/>

<speciesReference species="M_coa_c"/>

</listOfProducts>

<kineticLaw>

<math xmlns="<http://www.w3.org/1998/Math/MathML>">

<ci> FLUX_VALUE </ci>

</math>

<listOfParameters>

<parameter id="LOWER_BOUND" units="mmol_per_gDW_per_hr" value="0"/>

<parameter id="UPPER_BOUND" units="mmol_per_gDW_per_hr" value="1000"/>

<parameter id="FLUX_VALUE" units="mmol_per_gDW_per_hr" value="0"/>

<parameter id="OBJECTIVE_COEFFICIENT" units="dimensionless" value="0"/>

</listOfParameters>

</kineticLaw>

</reaction>

<reaction name="RE3151" id="R_RE3151C" metaid="_metaR_RE3151C" reversible="false" sboTerm="SBO:0000176">

<notes>

<body xmlns="<http://www.w3.org/1999/xhtml>">

<p>GENE_ASSOCIATION: </p>

<p>SUBSYSTEM: Fatty acid oxidation</p>

<p>EC Number: </p>

<p>Confidence Level: 0</p>

<p>AUTHORS: PMID:10970790</p>

<p>NOTES: </p>

</body>

</notes>

<listOfReactants>

<speciesReference species="M_arachdcoa_c"/>

<speciesReference species="M_h_c"/>

</listOfReactants>

<listOfProducts>

<speciesReference species="M_co2_c"/>

<speciesReference species="M_coa_c"/>

</listOfProducts>

<kineticLaw>

<math xmlns="<http://www.w3.org/1998/Math/MathML>">

<ci> FLUX_VALUE </ci>

</math>

<listOfParameters>

<parameter id="LOWER_BOUND" units="mmol_per_gDW_per_hr" value="0"/>

<parameter id="UPPER_BOUND" units="mmol_per_gDW_per_hr" value="1000"/>

<parameter id="FLUX_VALUE" units="mmol_per_gDW_per_hr" value="0"/>

<parameter id="OBJECTIVE_COEFFICIENT" units="dimensionless" value="0"/>

</listOfParameters>

</kineticLaw>

</reaction>

<reaction name="ammonia peroxisomal transport" id="R_NH4tp" metaid="_metaR_NH4tp" reversible="true" sboTerm="SBO:0000185">

<notes>

<body xmlns="<http://www.w3.org/1999/xhtml>">

<p>GENE_ASSOCIATION: </p>

<p>SUBSYSTEM: Transport, peroxisomal</p>

<p>EC Number: </p>

<p>Confidence Level: 1</p>

<p>AUTHORS: </p>

<p>NOTES: NCD</p>

</body>

</notes>

<listOfReactants>

<speciesReference species="M_nh4_c"/>

</listOfReactants>

<listOfProducts>

<speciesReference species="M_nh4_x"/>

</listOfProducts>

<kineticLaw>

<math xmlns="<http://www.w3.org/1998/Math/MathML>">

<ci> FLUX_VALUE </ci>

</math>

<listOfParameters>

<parameter id="LOWER_BOUND" units="mmol_per_gDW_per_hr" value="-1000"/>

<parameter id="UPPER_BOUND" units="mmol_per_gDW_per_hr" value="1000"/>

<parameter id="FLUX_VALUE" units="mmol_per_gDW_per_hr" value="0"/>

<parameter id="OBJECTIVE_COEFFICIENT" units="dimensionless" value="0"/>

</listOfParameters>

</kineticLaw>

</reaction>

<reaction name="Adenosine monophosphate deaminase" id="R_AMPDA" metaid="_metaR_AMPDA" reversible="false" sboTerm="SBO:0000176">

<notes>

<body xmlns="<http://www.w3.org/1999/xhtml>">

<p>GENE_ASSOCIATION: (271.2) or (270.1) or (271.1) or (272.1) or (271.3)</p>

<p>SUBSYSTEM: Nucleotide interconversion</p>

<p>EC Number: 3.5.4.6</p>

<p>Confidence Level: 4</p>

<p>AUTHORS: PMID:1400401,PMID:2345176</p>

<p>NOTES: </p>

</body>

</notes>

<listOfReactants>

<speciesReference species="M_h2o_c"/>

<speciesReference species="M_amp_c"/>

<speciesReference species="M_h_c"/>

</listOfReactants>

<listOfProducts>

<speciesReference species="M_nh4_c"/>

<speciesReference species="M_imp_c"/>

</listOfProducts>

<kineticLaw>

<math xmlns="<http://www.w3.org/1998/Math/MathML>">

<ci> FLUX_VALUE </ci>

</math>

<listOfParameters>

<parameter id="LOWER_BOUND" units="mmol_per_gDW_per_hr" value="0"/>

<parameter id="UPPER_BOUND" units="mmol_per_gDW_per_hr" value="1000"/>

<parameter id="FLUX_VALUE" units="mmol_per_gDW_per_hr" value="0"/>

<parameter id="OBJECTIVE_COEFFICIENT" units="dimensionless" value="0"/>

</listOfParameters>

</kineticLaw>

</reaction>

<reaction name="ammonia nuclear transport" id="R_NH4tn" metaid="_metaR_NH4tn" reversible="true" sboTerm="SBO:0000185">

<notes>

<body xmlns="<http://www.w3.org/1999/xhtml>">

<p>GENE_ASSOCIATION: </p>

<p>SUBSYSTEM: Transport, nuclear</p>

<p>EC Number: </p>

<p>Confidence Level: 1</p>

<p>AUTHORS: </p>

<p>NOTES: IT</p>

</body>

</notes>

<listOfReactants>

<speciesReference species="M_nh4_c"/>

</listOfReactants>

<listOfProducts>

<speciesReference species="M_nh4_n"/>

</listOfProducts>

<kineticLaw>

<math xmlns="<http://www.w3.org/1998/Math/MathML>">

<ci> FLUX_VALUE </ci>

</math>

<listOfParameters>

<parameter id="LOWER_BOUND" units="mmol_per_gDW_per_hr" value="-1000"/>

<parameter id="UPPER_BOUND" units="mmol_per_gDW_per_hr" value="1000"/>

<parameter id="FLUX_VALUE" units="mmol_per_gDW_per_hr" value="0"/>

<parameter id="OBJECTIVE_COEFFICIENT" units="dimensionless" value="0"/>

</listOfParameters>

</kineticLaw>

</reaction>

<reaction name="IMP cyclohydrolase" id="R_IMPC" metaid="_metaR_IMPC" reversible="true" sboTerm="SBO:0000176">

<notes>

<body xmlns="<http://www.w3.org/1999/xhtml>">

<p>GENE_ASSOCIATION: 471.1</p>

<p>SUBSYSTEM: Purine synthesis</p>

<p>EC Number: 3.5.4.10</p>

<p>Confidence Level: 4</p>

<p>AUTHORS: PMID:11948179,PMID:14966129,PMID:9378707</p>

<p>NOTES: </p>

</body>

</notes>

<listOfReactants>

<speciesReference species="M_imp_c"/>

<speciesReference species="M_h2o_c"/>

</listOfReactants>

<listOfProducts>

<speciesReference species="M_fprica_c"/>

</listOfProducts>

<kineticLaw>

<math xmlns="<http://www.w3.org/1998/Math/MathML>">

<ci> FLUX_VALUE </ci>

</math>

<listOfParameters>

<parameter id="LOWER_BOUND" units="mmol_per_gDW_per_hr" value="-1000"/>

<parameter id="UPPER_BOUND" units="mmol_per_gDW_per_hr" value="1000"/>

<parameter id="FLUX_VALUE" units="mmol_per_gDW_per_hr" value="0"/>

<parameter id="OBJECTIVE_COEFFICIENT" units="dimensionless" value="0"/>

</listOfParameters>

</kineticLaw>

</reaction>

<reaction name="adentylate kinase (GTP)" id="R_ADK3" metaid="_metaR_ADK3" reversible="true" sboTerm="SBO:0000176">

<notes>

<body xmlns="<http://www.w3.org/1999/xhtml>">

<p>GENE_ASSOCIATION: (26289.1) or (26289.2)</p>

<p>SUBSYSTEM: Nucleotide interconversion</p>

<p>EC Number: </p>

<p>Confidence Level: 4</p>

<p>AUTHORS: PMID:9434148</p>

<p>NOTES: JLR</p>

</body>

</notes>

<listOfReactants>

<speciesReference species="M_amp_c"/>

</listOfReactants>

<listOfProducts>

<speciesReference species="M_gdp_c"/>

<speciesReference species="M_adp_c"/>

</listOfProducts>

<kineticLaw>

<math xmlns="<http://www.w3.org/1998/Math/MathML>">

<ci> FLUX_VALUE </ci>

</math>

<listOfParameters>

<parameter id="LOWER_BOUND" units="mmol_per_gDW_per_hr" value="-1000"/>

<parameter id="UPPER_BOUND" units="mmol_per_gDW_per_hr" value="1000"/>

<parameter id="FLUX_VALUE" units="mmol_per_gDW_per_hr" value="0"/>

<parameter id="OBJECTIVE_COEFFICIENT" units="dimensionless" value="0"/>

</listOfParameters>

</kineticLaw>

</reaction>

<reaction name="GDP intracellular transport" id="R_GDPtg" metaid="_metaR_GDPtg" reversible="true" sboTerm="SBO:0000185">

<notes>

<body xmlns="<http://www.w3.org/1999/xhtml>">

<p>GENE_ASSOCIATION: </p>

<p>SUBSYSTEM: Transport, golgi apparatus</p>

<p>EC Number: </p>

<p>Confidence Level: 1</p>

<p>AUTHORS: </p>

<p>NOTES: unknown mechanism for transport - likely translocated, unknown transporter/mechanism though NJ</p>

</body>

</notes>

<listOfReactants>

<speciesReference species="M_gdp_c"/>

</listOfReactants>

<listOfProducts>

<speciesReference species="M_gdp_g"/>

</listOfProducts>

<kineticLaw>

<math xmlns="<http://www.w3.org/1998/Math/MathML>">

<ci> FLUX_VALUE </ci>

</math>

<listOfParameters>

<parameter id="LOWER_BOUND" units="mmol_per_gDW_per_hr" value="-1000"/>

<parameter id="UPPER_BOUND" units="mmol_per_gDW_per_hr" value="1000"/>

<parameter id="FLUX_VALUE" units="mmol_per_gDW_per_hr" value="0"/>

<parameter id="OBJECTIVE_COEFFICIENT" units="dimensionless" value="0"/>

</listOfParameters>

</kineticLaw>

</reaction>

<reaction name="RE3422" id="R_RE3422C" metaid="_metaR_RE3422C" reversible="true" sboTerm="SBO:0000176">

<notes>

<body xmlns="<http://www.w3.org/1999/xhtml>">

<p>GENE_ASSOCIATION: </p>

<p>SUBSYSTEM: Eicosanoid metabolism</p>

<p>EC Number: </p>

<p>Confidence Level: 0</p>

<p>AUTHORS: PMID:11786541,PMID:8521498</p>

<p>NOTES: </p>

</body>

</notes>

<listOfReactants>

<speciesReference species="M_prostgd2_c"/>

</listOfReactants>

<listOfProducts>

<speciesReference species="M_h2o_c"/>

<speciesReference species="M_CE5304_c"/>

</listOfProducts>

<kineticLaw>

<math xmlns="<http://www.w3.org/1998/Math/MathML>">

<ci> FLUX_VALUE </ci>

</math>

<listOfParameters>

<parameter id="LOWER_BOUND" units="mmol_per_gDW_per_hr" value="-1000"/>

<parameter id="UPPER_BOUND" units="mmol_per_gDW_per_hr" value="1000"/>

<parameter id="FLUX_VALUE" units="mmol_per_gDW_per_hr" value="0"/>

<parameter id="OBJECTIVE_COEFFICIENT" units="dimensionless" value="0"/>

</listOfParameters>

</kineticLaw>

</reaction>

<reaction name="Prostaglandin G/H synthase" id="R_PGS" metaid="_metaR_PGS" reversible="false" sboTerm="SBO:0000176">

<notes>

<body xmlns="<http://www.w3.org/1999/xhtml>">

<p>GENE_ASSOCIATION: (5743.1) or (5742.1)</p>

<p>SUBSYSTEM: Eicosanoid metabolism</p>

<p>EC Number: 1.14.99.1</p>

<p>Confidence Level: 4</p>

<p>AUTHORS: PMID:14511332,PMID:1907252</p>

<p>NOTES: NJ</p>

</body>

</notes>

<listOfReactants>

<speciesReference species="M_arachd_c"/>

<speciesReference species="M_o2_c" stoichiometry="2"/>

<speciesReference species="M_h_c"/>

<speciesReference species="M_nadph_c"/>

</listOfReactants>

<listOfProducts>

<speciesReference species="M_prostgh2_c"/>

<speciesReference species="M_h2o_c"/>

<speciesReference species="M_nadp_c"/>

</listOfProducts>

<kineticLaw>

<math xmlns="<http://www.w3.org/1998/Math/MathML>">

<ci> FLUX_VALUE </ci>

</math>

<listOfParameters>

<parameter id="LOWER_BOUND" units="mmol_per_gDW_per_hr" value="0"/>

<parameter id="UPPER_BOUND" units="mmol_per_gDW_per_hr" value="1000"/>

<parameter id="FLUX_VALUE" units="mmol_per_gDW_per_hr" value="0"/>

<parameter id="OBJECTIVE_COEFFICIENT" units="dimensionless" value="0"/>

</listOfParameters>

</kineticLaw>

</reaction>

<reaction name="Prostaglandin-H2 D-isomerase [Precursor]" id="R_PGDI" metaid="_metaR_PGDI" reversible="true" sboTerm="SBO:0000176">

<notes>

<body xmlns="<http://www.w3.org/1999/xhtml>">

<p>GENE_ASSOCIATION: (5730.1) or (27306.1)</p>

<p>SUBSYSTEM: Eicosanoid metabolism</p>

<p>EC Number: 5.3.99.2</p>

<p>Confidence Level: 4</p>

<p>AUTHORS: PMID:9065498,PMID:9353279</p>

<p>NOTES: noted on RER, but also cytoplasmic structures NJ</p>

</body>

</notes>

<listOfReactants>

<speciesReference species="M_prostgh2_c"/>

</listOfReactants>

<listOfProducts>

<speciesReference species="M_prostgd2_c"/>

</listOfProducts>

<kineticLaw>

<math xmlns="<http://www.w3.org/1998/Math/MathML>">

<ci> FLUX_VALUE </ci>

</math>

<listOfParameters>

<parameter id="LOWER_BOUND" units="mmol_per_gDW_per_hr" value="-1000"/>

<parameter id="UPPER_BOUND" units="mmol_per_gDW_per_hr" value="1000"/>

<parameter id="FLUX_VALUE" units="mmol_per_gDW_per_hr" value="0"/>

<parameter id="OBJECTIVE_COEFFICIENT" units="dimensionless" value="0"/>

</listOfParameters>

</kineticLaw>

</reaction>

<reaction name="RE1077" id="R_RE1077C" metaid="_metaR_RE1077C" reversible="true" sboTerm="SBO:0000176">

<notes>

<body xmlns="<http://www.w3.org/1999/xhtml>">

<p>GENE_ASSOCIATION: </p>

<p>SUBSYSTEM: Eicosanoid metabolism</p>

<p>EC Number: 5.3.99.5</p>

<p>Confidence Level: 0</p>

<p>AUTHORS: PMID:11097184,PMID:6440597,PMID:6812567</p>

<p>NOTES: </p>

</body>

</notes>

<listOfReactants>

<speciesReference species="M_prostgh2_c"/>

</listOfReactants>

<listOfProducts>

<speciesReference species="M_CE1243_c"/>

<speciesReference species="M_h_c"/>

<speciesReference species="M_CE0737_c"/>

</listOfProducts>

<kineticLaw>

<math xmlns="<http://www.w3.org/1998/Math/MathML>">

<ci> FLUX_VALUE </ci>

</math>

<listOfParameters>

<parameter id="LOWER_BOUND" units="mmol_per_gDW_per_hr" value="-1000"/>

<parameter id="UPPER_BOUND" units="mmol_per_gDW_per_hr" value="1000"/>

<parameter id="FLUX_VALUE" units="mmol_per_gDW_per_hr" value="0"/>

<parameter id="OBJECTIVE_COEFFICIENT" units="dimensionless" value="0"/>

</listOfParameters>

</kineticLaw>

</reaction>

<reaction name="ATP-binding Cassette (ABC) TCDB:3.A.1.211.1" id="R_r1514" metaid="_metaR_r1514" reversible="false" sboTerm="SBO:0000185">

<notes>

<body xmlns="<http://www.w3.org/1999/xhtml>">

<p>GENE_ASSOCIATION: </p>

<p>SUBSYSTEM: Transport, extracellular</p>

<p>EC Number: </p>

<p>Confidence Level: 0</p>

<p>AUTHORS: PMID:16858612</p>

<p>NOTES: </p>

</body>

</notes>

<listOfReactants>

<speciesReference species="M_arachd_c"/>

<speciesReference species="M_h2o_c"/>

<speciesReference species="M_atp_c"/>

</listOfReactants>

<listOfProducts>

<speciesReference species="M_arachd_e"/>

<speciesReference species="M_adp_c"/>

<speciesReference species="M_h_c"/>

<speciesReference species="M_pi_c"/>

</listOfProducts>

<kineticLaw>

<math xmlns="<http://www.w3.org/1998/Math/MathML>">

<ci> FLUX_VALUE </ci>

</math>

<listOfParameters>

<parameter id="LOWER_BOUND" units="mmol_per_gDW_per_hr" value="0"/>

<parameter id="UPPER_BOUND" units="mmol_per_gDW_per_hr" value="1000"/>

<parameter id="FLUX_VALUE" units="mmol_per_gDW_per_hr" value="0"/>

<parameter id="OBJECTIVE_COEFFICIENT" units="dimensionless" value="0"/>

</listOfParameters>

</kineticLaw>

</reaction>

<reaction name="Facilitated diffusion" id="R_r1000" metaid="_metaR_r1000" reversible="true" sboTerm="SBO:0000185">

<notes>

<body xmlns="<http://www.w3.org/1999/xhtml>">

<p>GENE_ASSOCIATION: </p>

<p>SUBSYSTEM: Transport, endoplasmic reticular</p>

<p>EC Number: </p>

<p>Confidence Level: 0</p>

<p>AUTHORS: PMID:14598172,PMID:1735445,PMID:18406340,PMID:1988962,PMID:2351134,PMID:2355017,PMID:6361812,PMID:7892212,PMID:8132483</p>

<p>NOTES: </p>

</body>

</notes>

<listOfReactants>

<speciesReference species="M_crn_c"/>

</listOfReactants>

<listOfProducts>

<speciesReference species="M_HC10854_c"/>

</listOfProducts>

<kineticLaw>

<math xmlns="<http://www.w3.org/1998/Math/MathML>">

<ci> FLUX_VALUE </ci>

</math>

<listOfParameters>

<parameter id="LOWER_BOUND" units="mmol_per_gDW_per_hr" value="-1000"/>

<parameter id="UPPER_BOUND" units="mmol_per_gDW_per_hr" value="1000"/>

<parameter id="FLUX_VALUE" units="mmol_per_gDW_per_hr" value="0"/>

<parameter id="OBJECTIVE_COEFFICIENT" units="dimensionless" value="0"/>

</listOfParameters>

</kineticLaw>

</reaction>

<reaction name="Utilized transport" id="R_r2507" metaid="_metaR_r2507" reversible="true" sboTerm="SBO:0000185">

<notes>

<body xmlns="<http://www.w3.org/1999/xhtml>">

<p>GENE_ASSOCIATION: </p>

<p>SUBSYSTEM: Transport, endoplasmic reticular</p>

<p>EC Number: </p>

<p>Confidence Level: 0</p>

<p>AUTHORS: PMID:17466261</p>

<p>NOTES: </p>

</body>

</notes>

<listOfReactants>

<speciesReference species="M_arachd_c"/>

</listOfReactants>

<listOfProducts>

<speciesReference species="M_crn_c"/>

<speciesReference species="M_arachd_r"/>

</listOfProducts>

<kineticLaw>

<math xmlns="<http://www.w3.org/1998/Math/MathML>">

<ci> FLUX_VALUE </ci>

</math>

<listOfParameters>

<parameter id="LOWER_BOUND" units="mmol_per_gDW_per_hr" value="-1000"/>

<parameter id="UPPER_BOUND" units="mmol_per_gDW_per_hr" value="1000"/>

<parameter id="FLUX_VALUE" units="mmol_per_gDW_per_hr" value="0"/>

<parameter id="OBJECTIVE_COEFFICIENT" units="dimensionless" value="0"/>

</listOfParameters>

</kineticLaw>

</reaction>

<reaction name="Palmitoyl-CoA:L-carnitine O-palmitoyltransferase Fatty acid metabolism EC:2.3.1.21" id="R_r0439" metaid="_metaR_r0439" reversible="true" sboTerm="SBO:0000176">

<notes>

<body xmlns="<http://www.w3.org/1999/xhtml>">

<p>GENE_ASSOCIATION: </p>

<p>SUBSYSTEM: Fatty acid oxidation</p>

<p>EC Number: 2.3.1.21</p>

<p>Confidence Level: 0</p>

<p>AUTHORS: PMID:1735445,PMID:1988962,PMID:2355017,PMID:6361812,PMID:7892212</p>

<p>NOTES: </p>

</body>

</notes>

<listOfReactants>

<speciesReference species="M_crn_c"/>

</listOfReactants>

<listOfProducts>

<speciesReference species="M_HC10855_c"/>

<speciesReference species="M_coa_c"/>

</listOfProducts>

<kineticLaw>

<math xmlns="<http://www.w3.org/1998/Math/MathML>">

<ci> FLUX_VALUE </ci>

</math>

<listOfParameters>

<parameter id="LOWER_BOUND" units="mmol_per_gDW_per_hr" value="-1000"/>

<parameter id="UPPER_BOUND" units="mmol_per_gDW_per_hr" value="1000"/>

<parameter id="FLUX_VALUE" units="mmol_per_gDW_per_hr" value="0"/>

<parameter id="OBJECTIVE_COEFFICIENT" units="dimensionless" value="0"/>

</listOfParameters>

</kineticLaw>

</reaction>

<reaction name="Palmitoyl-CoA:L-carnitine O-palmitoyltransferase Fatty acid metabolism EC:2.3.1.21" id="R_r0445" metaid="_metaR_r0445" reversible="true" sboTerm="SBO:0000176">

<notes>

<body xmlns="<http://www.w3.org/1999/xhtml>">

<p>GENE_ASSOCIATION: (AI971036.1) or (3417.1) or (3418.1)</p>

<p>SUBSYSTEM: Fatty acid oxidation</p>

<p>EC Number: 2.3.1.21</p>

<p>Confidence Level: 0</p>

<p>AUTHORS: PMID:1735445,PMID:1988962,PMID:2355017,PMID:6361812,PMID:7892212</p>

<p>NOTES: </p>

</body>

</notes>

<listOfReactants>

<speciesReference species="M_crn_c"/>

</listOfReactants>

<listOfProducts>

<speciesReference species="M_HC10854_c"/>

<speciesReference species="M_coa_c"/>

</listOfProducts>

<kineticLaw>

<math xmlns="<http://www.w3.org/1998/Math/MathML>">

<ci> FLUX_VALUE </ci>

</math>

<listOfParameters>

<parameter id="LOWER_BOUND" units="mmol_per_gDW_per_hr" value="-1000"/>

<parameter id="UPPER_BOUND" units="mmol_per_gDW_per_hr" value="1000"/>

<parameter id="FLUX_VALUE" units="mmol_per_gDW_per_hr" value="0"/>

<parameter id="OBJECTIVE_COEFFICIENT" units="dimensionless" value="0"/>

</listOfParameters>

</kineticLaw>

</reaction>

<reaction name="Facilitated diffusion" id="R_r1003" metaid="_metaR_r1003" reversible="false" sboTerm="SBO:0000185">

<notes>

<body xmlns="<http://www.w3.org/1999/xhtml>">

<p>GENE_ASSOCIATION: </p>

<p>SUBSYSTEM: Transport, peroxisomal</p>

<p>EC Number: </p>

<p>Confidence Level: 0</p>

<p>AUTHORS: PMID:14598172,PMID:1735445,PMID:18406340,PMID:1988962,PMID:2351134,PMID:2355017,PMID:6361812,PMID:7892212,PMID:8132483</p>

<p>NOTES: </p>

</body>

</notes>

<listOfReactants>

<speciesReference species="M_crn_c"/>

</listOfReactants>

<listOfProducts>

<speciesReference species="M_HC10855_c"/>

</listOfProducts>

<kineticLaw>

<math xmlns="<http://www.w3.org/1998/Math/MathML>">

<ci> FLUX_VALUE </ci>

</math>

<listOfParameters>

<parameter id="LOWER_BOUND" units="mmol_per_gDW_per_hr" value="0"/>

<parameter id="UPPER_BOUND" units="mmol_per_gDW_per_hr" value="1000"/>

<parameter id="FLUX_VALUE" units="mmol_per_gDW_per_hr" value="0"/>

<parameter id="OBJECTIVE_COEFFICIENT" units="dimensionless" value="0"/>

</listOfParameters>

</kineticLaw>

</reaction>

<reaction name="Facilitated diffusion" id="R_r1001" metaid="_metaR_r1001" reversible="true" sboTerm="SBO:0000185">

<notes>

<body xmlns="<http://www.w3.org/1999/xhtml>">

<p>GENE_ASSOCIATION: </p>

<p>SUBSYSTEM: Transport, mitochondrial</p>

<p>EC Number: </p>

<p>Confidence Level: 0</p>

<p>AUTHORS: PMID:14598172,PMID:1735445,PMID:18406340,PMID:1988962,PMID:2351134,PMID:2355017,PMID:6361812,PMID:7892212,PMID:8132483</p>

<p>NOTES: </p>

</body>

</notes>

<listOfReactants>

<speciesReference species="M_crn_c"/>

</listOfReactants>

<listOfProducts>

<speciesReference species="M_HC10854_c"/>

</listOfProducts>

<kineticLaw>

<math xmlns="<http://www.w3.org/1998/Math/MathML>">

<ci> FLUX_VALUE </ci>

</math>

<listOfParameters>

<parameter id="LOWER_BOUND" units="mmol_per_gDW_per_hr" value="-1000"/>

<parameter id="UPPER_BOUND" units="mmol_per_gDW_per_hr" value="1000"/>

<parameter id="FLUX_VALUE" units="mmol_per_gDW_per_hr" value="0"/>

<parameter id="OBJECTIVE_COEFFICIENT" units="dimensionless" value="0"/>

</listOfParameters>

</kineticLaw>

</reaction>

<reaction name="Facilitated diffusion" id="R_r1002" metaid="_metaR_r1002" reversible="true" sboTerm="SBO:0000185">

<notes>

<body xmlns="<http://www.w3.org/1999/xhtml>">

<p>GENE_ASSOCIATION: </p>

<p>SUBSYSTEM: Transport, endoplasmic reticular</p>

<p>EC Number: </p>

<p>Confidence Level: 0</p>

<p>AUTHORS: PMID:14598172,PMID:1735445,PMID:18406340,PMID:1988962,PMID:2351134,PMID:2355017,PMID:6361812,PMID:7892212,PMID:8132483</p>

<p>NOTES: </p>

</body>

</notes>

<listOfReactants>

<speciesReference species="M_crn_c"/>

</listOfReactants>

<listOfProducts>

<speciesReference species="M_HC10855_c"/>

</listOfProducts>

<kineticLaw>

<math xmlns="<http://www.w3.org/1998/Math/MathML>">

<ci> FLUX_VALUE </ci>

</math>

<listOfParameters>

<parameter id="LOWER_BOUND" units="mmol_per_gDW_per_hr" value="-1000"/>

<parameter id="UPPER_BOUND" units="mmol_per_gDW_per_hr" value="1000"/>

<parameter id="FLUX_VALUE" units="mmol_per_gDW_per_hr" value="0"/>

<parameter id="OBJECTIVE_COEFFICIENT" units="dimensionless" value="0"/>

</listOfParameters>

</kineticLaw>

</reaction>

<reaction name="Virtual reaction/potential definition" id="R_r1329" metaid="_metaR_r1329" reversible="true" sboTerm="SBO:0000176">

<notes>

<body xmlns="<http://www.w3.org/1999/xhtml>">

<p>GENE_ASSOCIATION: </p>

<p>SUBSYSTEM: Unassigned</p>

<p>EC Number: </p>

<p>Confidence Level: 0</p>

<p>AUTHORS: PMID:17466261</p>

<p>NOTES: </p>

</body>

</notes>

<listOfReactants>

<speciesReference species="M_fadh2_c"/>

</listOfReactants>

<listOfProducts>

<speciesReference species="M_HC02114_c"/>

</listOfProducts>

<kineticLaw>

<math xmlns="<http://www.w3.org/1998/Math/MathML>">

<ci> FLUX_VALUE </ci>

</math>

<listOfParameters>

<parameter id="LOWER_BOUND" units="mmol_per_gDW_per_hr" value="-1000"/>

<parameter id="UPPER_BOUND" units="mmol_per_gDW_per_hr" value="1000"/>

<parameter id="FLUX_VALUE" units="mmol_per_gDW_per_hr" value="0"/>

<parameter id="OBJECTIVE_COEFFICIENT" units="dimensionless" value="0"/>

</listOfParameters>

</kineticLaw>

</reaction>

<reaction name="dihydroceramide desaturase" id="R_DHCRD2" metaid="_metaR_DHCRD2" reversible="false" sboTerm="SBO:0000176">

<notes>

<body xmlns="<http://www.w3.org/1999/xhtml>">

<p>GENE_ASSOCIATION: (8560.1) or (8560.2)</p>

<p>SUBSYSTEM: Sphingolipid metabolism</p>

<p>EC Number: </p>

<p>Confidence Level: 4</p>

<p>AUTHORS: PMID:9188692</p>

<p>NOTES: NJ</p>

</body>

</notes>

<listOfReactants>

<speciesReference species="M_fad_c"/>

</listOfReactants>

<listOfProducts>

<speciesReference species="M_fadh2_c"/>

</listOfProducts>

<kineticLaw>

<math xmlns="<http://www.w3.org/1998/Math/MathML>">

<ci> FLUX_VALUE </ci>

</math>

<listOfParameters>

<parameter id="LOWER_BOUND" units="mmol_per_gDW_per_hr" value="0"/>

<parameter id="UPPER_BOUND" units="mmol_per_gDW_per_hr" value="1000"/>

<parameter id="FLUX_VALUE" units="mmol_per_gDW_per_hr" value="0"/>

<parameter id="OBJECTIVE_COEFFICIENT" units="dimensionless" value="0"/>

</listOfParameters>

</kineticLaw>

</reaction>

<reaction name="Proline dehydrogenase" id="R_PROD2" metaid="_metaR_PROD2" reversible="false" sboTerm="SBO:0000176">

<notes>

<body xmlns="<http://www.w3.org/1999/xhtml>">

<p>GENE_ASSOCIATION: 58510.1</p>

<p>SUBSYSTEM: Arginine and Proline Metabolism</p>

<p>EC Number: 1.5.99.8</p>

<p>Confidence Level: 2</p>

<p>AUTHORS: PMID:10192398</p>

<p>NOTES: JLR</p>

</body>

</notes>

<listOfReactants>

<speciesReference species="M_fad_c"/>

</listOfReactants>

<listOfProducts>

<speciesReference species="M_fadh2_c"/>

<speciesReference species="M_h_c"/>

</listOfProducts>

<kineticLaw>

<math xmlns="<http://www.w3.org/1998/Math/MathML>">

<ci> FLUX_VALUE </ci>

</math>

<listOfParameters>

<parameter id="LOWER_BOUND" units="mmol_per_gDW_per_hr" value="0"/>

<parameter id="UPPER_BOUND" units="mmol_per_gDW_per_hr" value="1000"/>

<parameter id="FLUX_VALUE" units="mmol_per_gDW_per_hr" value="0"/>

<parameter id="OBJECTIVE_COEFFICIENT" units="dimensionless" value="0"/>

</listOfParameters>

</kineticLaw>

</reaction>

<reaction name="Postulated transport reaction" id="R_r1290" metaid="_metaR_r1290" reversible="true" sboTerm="SBO:0000185">

<notes>

<body xmlns="<http://www.w3.org/1999/xhtml>">

<p>GENE_ASSOCIATION: </p>

<p>SUBSYSTEM: Unassigned</p>

<p>EC Number: 6.2.1.3</p>

<p>Confidence Level: 0</p>

<p>AUTHORS: PMID:8584017</p>

<p>NOTES: </p>

</body>

</notes>

<listOfReactants>

<speciesReference species="M_fad_c"/>

</listOfReactants>

<listOfProducts>

<speciesReference species="M_fad_m"/>

<speciesReference species="M_fadh2_c"/>

</listOfProducts>

<kineticLaw>

<math xmlns="<http://www.w3.org/1998/Math/MathML>">

<ci> FLUX_VALUE </ci>

</math>

<listOfParameters>

<parameter id="LOWER_BOUND" units="mmol_per_gDW_per_hr" value="-1000"/>

<parameter id="UPPER_BOUND" units="mmol_per_gDW_per_hr" value="1000"/>

<parameter id="FLUX_VALUE" units="mmol_per_gDW_per_hr" value="0"/>

<parameter id="OBJECTIVE_COEFFICIENT" units="dimensionless" value="0"/>

</listOfParameters>

</kineticLaw>

</reaction>

<reaction name="RE3347" id="R_RE3347C" metaid="_metaR_RE3347C" reversible="true" sboTerm="SBO:0000176">

<notes>

<body xmlns="<http://www.w3.org/1999/xhtml>">

<p>GENE_ASSOCIATION: </p>

<p>SUBSYSTEM: Vitamin B2 metabolism</p>

<p>EC Number: 1.5.1.29</p>

<p>Confidence Level: 0</p>

<p>AUTHORS: PMID:10620517</p>

<p>NOTES: </p>

</body>

</notes>

<listOfReactants>

<speciesReference species="M_fad_c"/>

<speciesReference species="M_h_c"/>

<speciesReference species="M_nadh_c"/>

</listOfReactants>

<listOfProducts>

<speciesReference species="M_fadh2_c"/>

<speciesReference species="M_nad_c"/>

</listOfProducts>

<kineticLaw>

<math xmlns="<http://www.w3.org/1998/Math/MathML>">

<ci> FLUX_VALUE </ci>

</math>

<listOfParameters>

<parameter id="LOWER_BOUND" units="mmol_per_gDW_per_hr" value="-1000"/>

<parameter id="UPPER_BOUND" units="mmol_per_gDW_per_hr" value="1000"/>

<parameter id="FLUX_VALUE" units="mmol_per_gDW_per_hr" value="0"/>

<parameter id="OBJECTIVE_COEFFICIENT" units="dimensionless" value="0"/>

</listOfParameters>

</kineticLaw>

</reaction>

<reaction name="Transport of fad into mitochondria" id="R_FADtm" metaid="_metaR_FADtm" reversible="true" sboTerm="SBO:0000185">

<notes>

<body xmlns="<http://www.w3.org/1999/xhtml>">

<p>GENE_ASSOCIATION: </p>

<p>SUBSYSTEM: Transport, mitochondrial</p>

<p>EC Number: </p>

<p>Confidence Level: 4</p>

<p>AUTHORS: </p>

<p>NOTES: </p>

</body>

</notes>

<listOfReactants>

<speciesReference species="M_fad_c"/>

</listOfReactants>

<listOfProducts>

<speciesReference species="M_fad_m"/>

</listOfProducts>

<kineticLaw>

<math xmlns="<http://www.w3.org/1998/Math/MathML>">

<ci> FLUX_VALUE </ci>

</math>

<listOfParameters>

<parameter id="LOWER_BOUND" units="mmol_per_gDW_per_hr" value="-1000"/>

<parameter id="UPPER_BOUND" units="mmol_per_gDW_per_hr" value="1000"/>

<parameter id="FLUX_VALUE" units="mmol_per_gDW_per_hr" value="0"/>

<parameter id="OBJECTIVE_COEFFICIENT" units="dimensionless" value="0"/>

</listOfParameters>

</kineticLaw>

</reaction>

<reaction name="RE3449" id="R_RE3449C" metaid="_metaR_RE3449C" reversible="true" sboTerm="SBO:0000176">

<notes>

<body xmlns="<http://www.w3.org/1999/xhtml>">

<p>GENE_ASSOCIATION: </p>

<p>SUBSYSTEM: Eicosanoid metabolism</p>

<p>EC Number: </p>

<p>Confidence Level: 0</p>

<p>AUTHORS: PMID:2123555</p>

<p>NOTES: </p>

</body>

</notes>

<listOfReactants>

<speciesReference species="M_arachd_c" stoichiometry="3"/>

<speciesReference species="M_h_c" stoichiometry="5"/>

</listOfReactants>

<listOfProducts>

<speciesReference species="M_CE5535_c" stoichiometry="3"/>

<speciesReference species="M_h2o_c" stoichiometry="4"/>

</listOfProducts>

<kineticLaw>

<math xmlns="<http://www.w3.org/1998/Math/MathML>">

<ci> FLUX_VALUE </ci>

</math>

<listOfParameters>

<parameter id="LOWER_BOUND" units="mmol_per_gDW_per_hr" value="-1000"/>

<parameter id="UPPER_BOUND" units="mmol_per_gDW_per_hr" value="1000"/>

<parameter id="FLUX_VALUE" units="mmol_per_gDW_per_hr" value="0"/>

<parameter id="OBJECTIVE_COEFFICIENT" units="dimensionless" value="0"/>

</listOfParameters>

</kineticLaw>

</reaction>

<reaction name="RE3451" id="R_RE3451C" metaid="_metaR_RE3451C" reversible="false" sboTerm="SBO:0000176">

<notes>

<body xmlns="<http://www.w3.org/1999/xhtml>">

<p>GENE_ASSOCIATION: </p>

<p>SUBSYSTEM: Eicosanoid metabolism</p>

<p>EC Number: </p>

<p>Confidence Level: 0</p>

<p>AUTHORS: PMID:2123555</p>

<p>NOTES: </p>

</body>

</notes>

<listOfReactants>

<speciesReference species="M_CE5535_c"/>

<speciesReference species="M_o2_c"/>

</listOfReactants>

<listOfProducts>

<speciesReference species="M_CE5534_c"/>

</listOfProducts>

<kineticLaw>

<math xmlns="<http://www.w3.org/1998/Math/MathML>">

<ci> FLUX_VALUE </ci>

</math>

<listOfParameters>

<parameter id="LOWER_BOUND" units="mmol_per_gDW_per_hr" value="0"/>

<parameter id="UPPER_BOUND" units="mmol_per_gDW_per_hr" value="1000"/>

<parameter id="FLUX_VALUE" units="mmol_per_gDW_per_hr" value="0"/>

<parameter id="OBJECTIVE_COEFFICIENT" units="dimensionless" value="0"/>

</listOfParameters>

</kineticLaw>

</reaction>

<reaction name="RE3450" id="R_RE3450C" metaid="_metaR_RE3450C" reversible="true" sboTerm="SBO:0000176">

<notes>

<body xmlns="<http://www.w3.org/1999/xhtml>">

<p>GENE_ASSOCIATION: </p>

<p>SUBSYSTEM: Eicosanoid metabolism</p>

<p>EC Number: </p>

<p>Confidence Level: 0</p>

<p>AUTHORS: PMID:2123555</p>

<p>NOTES: </p>

</body>

</notes>

<listOfReactants>

<speciesReference species="M_CE5534_c"/>

</listOfReactants>

<listOfProducts>

<speciesReference species="M_h2o_c"/>

<speciesReference species="M_CE5828_c"/>

</listOfProducts>

<kineticLaw>

<math xmlns="<http://www.w3.org/1998/Math/MathML>">

<ci> FLUX_VALUE </ci>

</math>

<listOfParameters>

<parameter id="LOWER_BOUND" units="mmol_per_gDW_per_hr" value="-1000"/>

<parameter id="UPPER_BOUND" units="mmol_per_gDW_per_hr" value="1000"/>

<parameter id="FLUX_VALUE" units="mmol_per_gDW_per_hr" value="0"/>

<parameter id="OBJECTIVE_COEFFICIENT" units="dimensionless" value="0"/>

</listOfParameters>

</kineticLaw>

</reaction>

<reaction name="RE3455" id="R_RE3455C" metaid="_metaR_RE3455C" reversible="false" sboTerm="SBO:0000176">

<notes>

<body xmlns="<http://www.w3.org/1999/xhtml>">

<p>GENE_ASSOCIATION: </p>

<p>SUBSYSTEM: Eicosanoid metabolism</p>

<p>EC Number: </p>

<p>Confidence Level: 0</p>

<p>AUTHORS: PMID:2123555</p>

<p>NOTES: </p>

</body>

</notes>

<listOfReactants>

<speciesReference species="M_arachd_c" stoichiometry="4"/>

<speciesReference species="M_o2_c" stoichiometry="5"/>

</listOfReactants>

<listOfProducts>

<speciesReference species="M_CE5928_c" stoichiometry="4"/>

<speciesReference species="M_h2o_c" stoichiometry="2"/>

</listOfProducts>

<kineticLaw>

<math xmlns="<http://www.w3.org/1998/Math/MathML>">

<ci> FLUX_VALUE </ci>

</math>

<listOfParameters>

<parameter id="LOWER_BOUND" units="mmol_per_gDW_per_hr" value="0"/>

<parameter id="UPPER_BOUND" units="mmol_per_gDW_per_hr" value="1000"/>

<parameter id="FLUX_VALUE" units="mmol_per_gDW_per_hr" value="0"/>

<parameter id="OBJECTIVE_COEFFICIENT" units="dimensionless" value="0"/>

</listOfParameters>

</kineticLaw>

</reaction>

<reaction name="RE3456" id="R_RE3456C" metaid="_metaR_RE3456C" reversible="true" sboTerm="SBO:0000176">

<notes>

<body xmlns="<http://www.w3.org/1999/xhtml>">

<p>GENE_ASSOCIATION: </p>

<p>SUBSYSTEM: Eicosanoid metabolism</p>

<p>EC Number: </p>

<p>Confidence Level: 0</p>

<p>AUTHORS: PMID:2123555</p>

<p>NOTES: </p>

</body>

</notes>

<listOfReactants>

<speciesReference species="M_CE5930_c"/>

</listOfReactants>

<listOfProducts>

<speciesReference species="M_CE5931_c"/>

<speciesReference species="M_h2o_c"/>

</listOfProducts>

<kineticLaw>

<math xmlns="<http://www.w3.org/1998/Math/MathML>">

<ci> FLUX_VALUE </ci>

</math>

<listOfParameters>

<parameter id="LOWER_BOUND" units="mmol_per_gDW_per_hr" value="-1000"/>

<parameter id="UPPER_BOUND" units="mmol_per_gDW_per_hr" value="1000"/>

<parameter id="FLUX_VALUE" units="mmol_per_gDW_per_hr" value="0"/>

<parameter id="OBJECTIVE_COEFFICIENT" units="dimensionless" value="0"/>

</listOfParameters>

</kineticLaw>

</reaction>

<reaction name="RE3457" id="R_RE3457C" metaid="_metaR_RE3457C" reversible="false" sboTerm="SBO:0000176">

<notes>

<body xmlns="<http://www.w3.org/1999/xhtml>">

<p>GENE_ASSOCIATION: </p>

<p>SUBSYSTEM: Eicosanoid metabolism</p>

<p>EC Number: </p>

<p>Confidence Level: 0</p>

<p>AUTHORS: PMID:2123555</p>

<p>NOTES: </p>

</body>

</notes>

<listOfReactants>

<speciesReference species="M_CE5928_c"/>

<speciesReference species="M_o2_c"/>

</listOfReactants>

<listOfProducts>

<speciesReference species="M_CE5930_c"/>

</listOfProducts>

<kineticLaw>

<math xmlns="<http://www.w3.org/1998/Math/MathML>">

<ci> FLUX_VALUE </ci>

</math>

<listOfParameters>

<parameter id="LOWER_BOUND" units="mmol_per_gDW_per_hr" value="0"/>

<parameter id="UPPER_BOUND" units="mmol_per_gDW_per_hr" value="1000"/>

<parameter id="FLUX_VALUE" units="mmol_per_gDW_per_hr" value="0"/>

<parameter id="OBJECTIVE_COEFFICIENT" units="dimensionless" value="0"/>

</listOfParameters>

</kineticLaw>

</reaction>

<reaction name="RE3452" id="R_RE3452C" metaid="_metaR_RE3452C" reversible="true" sboTerm="SBO:0000176">

<notes>

<body xmlns="<http://www.w3.org/1999/xhtml>">

<p>GENE_ASSOCIATION: </p>

<p>SUBSYSTEM: Eicosanoid metabolism</p>

<p>EC Number: </p>

<p>Confidence Level: 0</p>

<p>AUTHORS: PMID:2123555</p>

<p>NOTES: </p>

</body>

</notes>

<listOfReactants>

<speciesReference species="M_arachd_c" stoichiometry="3"/>

<speciesReference species="M_h_c" stoichiometry="5"/>

</listOfReactants>

<listOfProducts>

<speciesReference species="M_h2o_c" stoichiometry="4"/>

<speciesReference species="M_CE5925_c" stoichiometry="3"/>

</listOfProducts>

<kineticLaw>

<math xmlns="<http://www.w3.org/1998/Math/MathML>">

<ci> FLUX_VALUE </ci>

</math>

<listOfParameters>

<parameter id="LOWER_BOUND" units="mmol_per_gDW_per_hr" value="-1000"/>

<parameter id="UPPER_BOUND" units="mmol_per_gDW_per_hr" value="1000"/>

<parameter id="FLUX_VALUE" units="mmol_per_gDW_per_hr" value="0"/>

<parameter id="OBJECTIVE_COEFFICIENT" units="dimensionless" value="0"/>

</listOfParameters>

</kineticLaw>

</reaction>

<reaction name="RE3453" id="R_RE3453C" metaid="_metaR_RE3453C" reversible="true" sboTerm="SBO:0000176">

<notes>

<body xmlns="<http://www.w3.org/1999/xhtml>">

<p>GENE_ASSOCIATION: </p>

<p>SUBSYSTEM: Eicosanoid metabolism</p>

<p>EC Number: </p>

<p>Confidence Level: 0</p>

<p>AUTHORS: PMID:2123555</p>

<p>NOTES: </p>

</body>

</notes>

<listOfReactants>

<speciesReference species="M_CE5926_c"/>

<speciesReference species="M_h_c" stoichiometry="4"/>

</listOfReactants>

<listOfProducts>

<speciesReference species="M_CE5929_c"/>

<speciesReference species="M_h2o_c"/>

</listOfProducts>

<kineticLaw>

<math xmlns="<http://www.w3.org/1998/Math/MathML>">

<ci> FLUX_VALUE </ci>

</math>

<listOfParameters>

<parameter id="LOWER_BOUND" units="mmol_per_gDW_per_hr" value="-1000"/>

<parameter id="UPPER_BOUND" units="mmol_per_gDW_per_hr" value="1000"/>

<parameter id="FLUX_VALUE" units="mmol_per_gDW_per_hr" value="0"/>

<parameter id="OBJECTIVE_COEFFICIENT" units="dimensionless" value="0"/>

</listOfParameters>

</kineticLaw>

</reaction>

<reaction name="RE3454" id="R_RE3454C" metaid="_metaR_RE3454C" reversible="false" sboTerm="SBO:0000176">

<notes>

<body xmlns="<http://www.w3.org/1999/xhtml>">

<p>GENE_ASSOCIATION: </p>

<p>SUBSYSTEM: Eicosanoid metabolism</p>

<p>EC Number: </p>

<p>Confidence Level: 0</p>

<p>AUTHORS: PMID:2123555</p>

<p>NOTES: </p>

</body>

</notes>

<listOfReactants>

<speciesReference species="M_CE5925_c"/>

<speciesReference species="M_o2_c"/>

</listOfReactants>

<listOfProducts>

<speciesReference species="M_CE5926_c"/>

</listOfProducts>

<kineticLaw>

<math xmlns="<http://www.w3.org/1998/Math/MathML>">

<ci> FLUX_VALUE </ci>

</math>

<listOfParameters>

<parameter id="LOWER_BOUND" units="mmol_per_gDW_per_hr" value="0"/>

<parameter id="UPPER_BOUND" units="mmol_per_gDW_per_hr" value="1000"/>

<parameter id="FLUX_VALUE" units="mmol_per_gDW_per_hr" value="0"/>

<parameter id="OBJECTIVE_COEFFICIENT" units="dimensionless" value="0"/>

</listOfParameters>

</kineticLaw>

</reaction>

<reaction name="RE3460" id="R_RE3460C" metaid="_metaR_RE3460C" reversible="false" sboTerm="SBO:0000176">

<notes>

<body xmlns="<http://www.w3.org/1999/xhtml>">

<p>GENE_ASSOCIATION: </p>

<p>SUBSYSTEM: Eicosanoid metabolism</p>

<p>EC Number: </p>

<p>Confidence Level: 0</p>

<p>AUTHORS: PMID:2123555</p>

<p>NOTES: </p>

</body>

</notes>

<listOfReactants>

<speciesReference species="M_CE5537_c"/>

<speciesReference species="M_o2_c"/>

</listOfReactants>

<listOfProducts>

<speciesReference species="M_CE5533_c"/>

</listOfProducts>

<kineticLaw>

<math xmlns="<http://www.w3.org/1998/Math/MathML>">

<ci> FLUX_VALUE </ci>

</math>

<listOfParameters>

<parameter id="LOWER_BOUND" units="mmol_per_gDW_per_hr" value="0"/>

<parameter id="UPPER_BOUND" units="mmol_per_gDW_per_hr" value="1000"/>

<parameter id="FLUX_VALUE" units="mmol_per_gDW_per_hr" value="0"/>

<parameter id="OBJECTIVE_COEFFICIENT" units="dimensionless" value="0"/>

</listOfParameters>

</kineticLaw>

</reaction>

<reaction name="RE3458" id="R_RE3458C" metaid="_metaR_RE3458C" reversible="true" sboTerm="SBO:0000176">

<notes>

<body xmlns="<http://www.w3.org/1999/xhtml>">

<p>GENE_ASSOCIATION: </p>

<p>SUBSYSTEM: Eicosanoid metabolism</p>

<p>EC Number: </p>

<p>Confidence Level: 0</p>

<p>AUTHORS: PMID:2123555</p>

<p>NOTES: </p>

</body>

</notes>

<listOfReactants>

<speciesReference species="M_arachd_c" stoichiometry="3"/>

<speciesReference species="M_h_c" stoichiometry="5"/>

</listOfReactants>

<listOfProducts>

<speciesReference species="M_CE5537_c" stoichiometry="3"/>

<speciesReference species="M_h2o_c" stoichiometry="4"/>

</listOfProducts>

<kineticLaw>

<math xmlns="<http://www.w3.org/1998/Math/MathML>">

<ci> FLUX_VALUE </ci>

</math>

<listOfParameters>

<parameter id="LOWER_BOUND" units="mmol_per_gDW_per_hr" value="-1000"/>

<parameter id="UPPER_BOUND" units="mmol_per_gDW_per_hr" value="1000"/>

<parameter id="FLUX_VALUE" units="mmol_per_gDW_per_hr" value="0"/>

<parameter id="OBJECTIVE_COEFFICIENT" units="dimensionless" value="0"/>

</listOfParameters>

</kineticLaw>

</reaction>

<reaction name="RE3459" id="R_RE3459C" metaid="_metaR_RE3459C" reversible="true" sboTerm="SBO:0000176">

<notes>

<body xmlns="<http://www.w3.org/1999/xhtml>">

<p>GENE_ASSOCIATION: </p>

<p>SUBSYSTEM: Eicosanoid metabolism</p>

<p>EC Number: </p>

<p>Confidence Level: 0</p>

<p>AUTHORS: PMID:2123555</p>

<p>NOTES: </p>

</body>

</notes>

<listOfReactants>

<speciesReference species="M_CE5533_c"/>

<speciesReference species="M_h_c" stoichiometry="4"/>

</listOfReactants>

<listOfProducts>

<speciesReference species="M_CE5924_c"/>

<speciesReference species="M_h2o_c"/>

</listOfProducts>

<kineticLaw>

<math xmlns="<http://www.w3.org/1998/Math/MathML>">

<ci> FLUX_VALUE </ci>

</math>

<listOfParameters>

<parameter id="LOWER_BOUND" units="mmol_per_gDW_per_hr" value="-1000"/>

<parameter id="UPPER_BOUND" units="mmol_per_gDW_per_hr" value="1000"/>

<parameter id="FLUX_VALUE" units="mmol_per_gDW_per_hr" value="0"/>

<parameter id="OBJECTIVE_COEFFICIENT" units="dimensionless" value="0"/>

</listOfParameters>

</kineticLaw>

</reaction>

<reaction name="Resistance-Nodulation-Cell Division (RND) TCDB:2.A.60.1.14" id="R_r2169" metaid="_metaR_r2169" reversible="true" sboTerm="SBO:0000185">

<notes>

<body xmlns="<http://www.w3.org/1999/xhtml>">

<p>GENE_ASSOCIATION: </p>

<p>SUBSYSTEM: Transport, extracellular</p>

<p>EC Number: </p>

<p>Confidence Level: 0</p>

<p>AUTHORS: PMID:11076394,PMID:11076396,PMID:14579113</p>

<p>NOTES: </p>

</body>

</notes>

<listOfReactants>

<speciesReference species="M_HC02199_c"/>

<speciesReference species="M_hco3_c"/>

</listOfReactants>

<listOfProducts>

<speciesReference species="M_HC02192_c"/>

<speciesReference species="M_hco3_e"/>

</listOfProducts>

<kineticLaw>

<math xmlns="<http://www.w3.org/1998/Math/MathML>">

<ci> FLUX_VALUE </ci>

</math>

<listOfParameters>

<parameter id="LOWER_BOUND" units="mmol_per_gDW_per_hr" value="-1000"/>

<parameter id="UPPER_BOUND" units="mmol_per_gDW_per_hr" value="1000"/>

<parameter id="FLUX_VALUE" units="mmol_per_gDW_per_hr" value="0"/>

<parameter id="OBJECTIVE_COEFFICIENT" units="dimensionless" value="0"/>

</listOfParameters>

</kineticLaw>

</reaction>

<reaction name="Resistance-Nodulation-Cell Division (RND) TCDB:2.A.60.1.14" id="R_r2485" metaid="_metaR_r2485" reversible="true" sboTerm="SBO:0000185">

<notes>

<body xmlns="<http://www.w3.org/1999/xhtml>">

<p>GENE_ASSOCIATION: </p>

<p>SUBSYSTEM: Transport, extracellular</p>

<p>EC Number: </p>

<p>Confidence Level: 0</p>

<p>AUTHORS: PMID:14579113</p>

<p>NOTES: </p>

</body>

</notes>

<listOfReactants>

<speciesReference species="M_HC02199_e"/>

<speciesReference species="M_hco3_e"/>

</listOfReactants>

<listOfProducts>

<speciesReference species="M_HC02199_c"/>

<speciesReference species="M_hco3_c"/>

</listOfProducts>

<kineticLaw>

<math xmlns="<http://www.w3.org/1998/Math/MathML>">

<ci> FLUX_VALUE </ci>

</math>

<listOfParameters>

<parameter id="LOWER_BOUND" units="mmol_per_gDW_per_hr" value="-1000"/>

<parameter id="UPPER_BOUND" units="mmol_per_gDW_per_hr" value="1000"/>

<parameter id="FLUX_VALUE" units="mmol_per_gDW_per_hr" value="0"/>

<parameter id="OBJECTIVE_COEFFICIENT" units="dimensionless" value="0"/>

</listOfParameters>

</kineticLaw>

</reaction>

<reaction name="Resistance-Nodulation-Cell Division (RND) TCDB:2.A.60.1.14" id="R_r2172" metaid="_metaR_r2172" reversible="true" sboTerm="SBO:0000185">

<notes>

<body xmlns="<http://www.w3.org/1999/xhtml>">

<p>GENE_ASSOCIATION: </p>

<p>SUBSYSTEM: Transport, extracellular</p>

<p>EC Number: </p>

<p>Confidence Level: 0</p>

<p>AUTHORS: PMID:11076394,PMID:11076396,PMID:14579113</p>

<p>NOTES: </p>

</body>

</notes>

<listOfReactants>

<speciesReference species="M_HC02199_c"/>

<speciesReference species="M_hco3_c"/>

</listOfReactants>

<listOfProducts>

<speciesReference species="M_HC02193_c"/>

<speciesReference species="M_hco3_e"/>

</listOfProducts>

<kineticLaw>

<math xmlns="<http://www.w3.org/1998/Math/MathML>">

<ci> FLUX_VALUE </ci>

</math>

<listOfParameters>

<parameter id="LOWER_BOUND" units="mmol_per_gDW_per_hr" value="-1000"/>

<parameter id="UPPER_BOUND" units="mmol_per_gDW_per_hr" value="1000"/>

<parameter id="FLUX_VALUE" units="mmol_per_gDW_per_hr" value="0"/>

<parameter id="OBJECTIVE_COEFFICIENT" units="dimensionless" value="0"/>

</listOfParameters>

</kineticLaw>

</reaction>

<reaction name="Resistance-Nodulation-Cell Division (RND) TCDB:2.A.60.1.14" id="R_r2175" metaid="_metaR_r2175" reversible="true" sboTerm="SBO:0000185">

<notes>

<body xmlns="<http://www.w3.org/1999/xhtml>">

<p>GENE_ASSOCIATION: </p>

<p>SUBSYSTEM: Transport, extracellular</p>

<p>EC Number: </p>

<p>Confidence Level: 0</p>

<p>AUTHORS: PMID:11076394,PMID:11076396,PMID:14579113</p>

<p>NOTES: </p>

</body>

</notes>

<listOfReactants>

<speciesReference species="M_HC02199_c"/>

<speciesReference species="M_hco3_c"/>

</listOfReactants>

<listOfProducts>

<speciesReference species="M_HC02194_c"/>

<speciesReference species="M_hco3_e"/>

</listOfProducts>

<kineticLaw>

<math xmlns="<http://www.w3.org/1998/Math/MathML>">

<ci> FLUX_VALUE </ci>

</math>

<listOfParameters>

<parameter id="LOWER_BOUND" units="mmol_per_gDW_per_hr" value="-1000"/>

<parameter id="UPPER_BOUND" units="mmol_per_gDW_per_hr" value="1000"/>

<parameter id="FLUX_VALUE" units="mmol_per_gDW_per_hr" value="0"/>

<parameter id="OBJECTIVE_COEFFICIENT" units="dimensionless" value="0"/>

</listOfParameters>

</kineticLaw>

</reaction>

<reaction name="Resistance-Nodulation-Cell Division (RND) TCDB:2.A.60.1.14" id="R_r2184" metaid="_metaR_r2184" reversible="true" sboTerm="SBO:0000185">

<notes>

<body xmlns="<http://www.w3.org/1999/xhtml>">

<p>GENE_ASSOCIATION: 6566.1</p>

<p>SUBSYSTEM: Transport, extracellular</p>

<p>EC Number: </p>

<p>Confidence Level: 0</p>

<p>AUTHORS: PMID:11076394,PMID:11076396,PMID:14579113</p>

<p>NOTES: </p>

</body>

</notes>

<listOfReactants>

<speciesReference species="M_HC02199_c"/>

<speciesReference species="M_hco3_c"/>

</listOfReactants>

<listOfProducts>

<speciesReference species="M_HC02197_c"/>

<speciesReference species="M_hco3_e"/>

</listOfProducts>

<kineticLaw>

<math xmlns="<http://www.w3.org/1998/Math/MathML>">

<ci> FLUX_VALUE </ci>

</math>

<listOfParameters>

<parameter id="LOWER_BOUND" units="mmol_per_gDW_per_hr" value="-1000"/>

<parameter id="UPPER_BOUND" units="mmol_per_gDW_per_hr" value="1000"/>

<parameter id="FLUX_VALUE" units="mmol_per_gDW_per_hr" value="0"/>

<parameter id="OBJECTIVE_COEFFICIENT" units="dimensionless" value="0"/>

</listOfParameters>

</kineticLaw>

</reaction>

<reaction name="Resistance-Nodulation-Cell Division (RND) TCDB:2.A.60.1.14" id="R_r2181" metaid="_metaR_r2181" reversible="true" sboTerm="SBO:0000185">

<notes>

<body xmlns="<http://www.w3.org/1999/xhtml>">

<p>GENE_ASSOCIATION: 6566.1</p>

<p>SUBSYSTEM: Transport, extracellular</p>

<p>EC Number: </p>

<p>Confidence Level: 0</p>

<p>AUTHORS: PMID:11076394,PMID:11076396,PMID:14579113</p>

<p>NOTES: </p>

</body>

</notes>

<listOfReactants>

<speciesReference species="M_HC02199_c"/>

<speciesReference species="M_hco3_c"/>

</listOfReactants>

<listOfProducts>

<speciesReference species="M_HC02196_c"/>

<speciesReference species="M_hco3_e"/>

</listOfProducts>

<kineticLaw>

<math xmlns="<http://www.w3.org/1998/Math/MathML>">

<ci> FLUX_VALUE </ci>

</math>

<listOfParameters>

<parameter id="LOWER_BOUND" units="mmol_per_gDW_per_hr" value="-1000"/>

<parameter id="UPPER_BOUND" units="mmol_per_gDW_per_hr" value="1000"/>

<parameter id="FLUX_VALUE" units="mmol_per_gDW_per_hr" value="0"/>

<parameter id="OBJECTIVE_COEFFICIENT" units="dimensionless" value="0"/>

</listOfParameters>

</kineticLaw>

</reaction>

<reaction name="Resistance-Nodulation-Cell Division (RND) TCDB:2.A.60.1.14" id="R_r2178" metaid="_metaR_r2178" reversible="true" sboTerm="SBO:0000185">

<notes>

<body xmlns="<http://www.w3.org/1999/xhtml>">

<p>GENE_ASSOCIATION: </p>

<p>SUBSYSTEM: Transport, extracellular</p>

<p>EC Number: </p>

<p>Confidence Level: 0</p>

<p>AUTHORS: PMID:11076394,PMID:11076396,PMID:14579113</p>

<p>NOTES: </p>

</body>

</notes>

<listOfReactants>

<speciesReference species="M_HC02199_c"/>

<speciesReference species="M_hco3_c"/>

</listOfReactants>

<listOfProducts>

<speciesReference species="M_HC02195_c"/>

<speciesReference species="M_hco3_e"/>

</listOfProducts>

<kineticLaw>

<math xmlns="<http://www.w3.org/1998/Math/MathML>">

<ci> FLUX_VALUE </ci>

</math>

<listOfParameters>

<parameter id="LOWER_BOUND" units="mmol_per_gDW_per_hr" value="-1000"/>

<parameter id="UPPER_BOUND" units="mmol_per_gDW_per_hr" value="1000"/>

<parameter id="FLUX_VALUE" units="mmol_per_gDW_per_hr" value="0"/>

<parameter id="OBJECTIVE_COEFFICIENT" units="dimensionless" value="0"/>

</listOfParameters>

</kineticLaw>

</reaction>

<reaction name="Resistance-Nodulation-Cell Division (RND) TCDB:2.A.60.1.14" id="R_r2190" metaid="_metaR_r2190" reversible="true" sboTerm="SBO:0000185">

<notes>

<body xmlns="<http://www.w3.org/1999/xhtml>">

<p>GENE_ASSOCIATION: 6566.1</p>

<p>SUBSYSTEM: Transport, extracellular</p>

<p>EC Number: </p>

<p>Confidence Level: 0</p>

<p>AUTHORS: PMID:11076394,PMID:11076396,PMID:14579113</p>

<p>NOTES: </p>

</body>

</notes>

<listOfReactants>

<speciesReference species="M_HC02199_c"/>

<speciesReference species="M_hco3_c"/>

</listOfReactants>

<listOfProducts>

<speciesReference species="M_HC02220_c"/>

<speciesReference species="M_hco3_e"/>

</listOfProducts>

<kineticLaw>

<math xmlns="<http://www.w3.org/1998/Math/MathML>">

<ci> FLUX_VALUE </ci>

</math>

<listOfParameters>

<parameter id="LOWER_BOUND" units="mmol_per_gDW_per_hr" value="-1000"/>

<parameter id="UPPER_BOUND" units="mmol_per_gDW_per_hr" value="1000"/>

<parameter id="FLUX_VALUE" units="mmol_per_gDW_per_hr" value="0"/>

<parameter id="OBJECTIVE_COEFFICIENT" units="dimensionless" value="0"/>

</listOfParameters>

</kineticLaw>

</reaction>

<reaction name="Resistance-Nodulation-Cell Division (RND) TCDB:2.A.60.1.14" id="R_r2212" metaid="_metaR_r2212" reversible="true" sboTerm="SBO:0000185">

<notes>

<body xmlns="<http://www.w3.org/1999/xhtml>">

<p>GENE_ASSOCIATION: 6566.1</p>

<p>SUBSYSTEM: Transport, extracellular</p>

<p>EC Number: </p>

<p>Confidence Level: 0</p>

<p>AUTHORS: PMID:11076396,PMID:14579113</p>

<p>NOTES: </p>

</body>

</notes>

<listOfReactants>

<speciesReference species="M_HC02199_c"/>

<speciesReference species="M_hco3_c"/>

</listOfReactants>

<listOfProducts>

<speciesReference species="M_HC02187_c"/>

<speciesReference species="M_hco3_e"/>

</listOfProducts>

<kineticLaw>

<math xmlns="<http://www.w3.org/1998/Math/MathML>">

<ci> FLUX_VALUE </ci>

</math>

<listOfParameters>

<parameter id="LOWER_BOUND" units="mmol_per_gDW_per_hr" value="-1000"/>

<parameter id="UPPER_BOUND" units="mmol_per_gDW_per_hr" value="1000"/>

<parameter id="FLUX_VALUE" units="mmol_per_gDW_per_hr" value="0"/>

<parameter id="OBJECTIVE_COEFFICIENT" units="dimensionless" value="0"/>

</listOfParameters>

</kineticLaw>

</reaction>

<reaction name="Resistance-Nodulation-Cell Division (RND) TCDB:2.A.60.1.5" id="R_r2239" metaid="_metaR_r2239" reversible="true" sboTerm="SBO:0000185">

<notes>

<body xmlns="<http://www.w3.org/1999/xhtml>">

<p>GENE_ASSOCIATION: </p>

<p>SUBSYSTEM: Transport, extracellular</p>

<p>EC Number: </p>

<p>Confidence Level: 0</p>

<p>AUTHORS: PMID:11076396,PMID:14579113</p>

<p>NOTES: </p>

</body>

</notes>

<listOfReactants>

<speciesReference species="M_HC02199_c"/>

<speciesReference species="M_hco3_c"/>

</listOfReactants>

<listOfProducts>

<speciesReference species="M_HC02180_c"/>

<speciesReference species="M_hco3_e"/>

</listOfProducts>

<kineticLaw>

<math xmlns="<http://www.w3.org/1998/Math/MathML>">

<ci> FLUX_VALUE </ci>

</math>

<listOfParameters>

<parameter id="LOWER_BOUND" units="mmol_per_gDW_per_hr" value="-1000"/>

<parameter id="UPPER_BOUND" units="mmol_per_gDW_per_hr" value="1000"/>

<parameter id="FLUX_VALUE" units="mmol_per_gDW_per_hr" value="0"/>

<parameter id="OBJECTIVE_COEFFICIENT" units="dimensionless" value="0"/>

</listOfParameters>

</kineticLaw>

</reaction>

<reaction name="Resistance-Nodulation-Cell Division (RND) TCDB:2.A.60.1.5" id="R_r2236" metaid="_metaR_r2236" reversible="true" sboTerm="SBO:0000185">

<notes>

<body xmlns="<http://www.w3.org/1999/xhtml>">

<p>GENE_ASSOCIATION: </p>

<p>SUBSYSTEM: Transport, extracellular</p>

<p>EC Number: </p>

<p>Confidence Level: 0</p>

<p>AUTHORS: PMID:11076396,PMID:14579113</p>

<p>NOTES: </p>

</body>

</notes>

<listOfReactants>

<speciesReference species="M_HC02199_c"/>

<speciesReference species="M_hco3_c"/>

</listOfReactants>

<listOfProducts>

<speciesReference species="M_HC02179_c"/>

<speciesReference species="M_hco3_e"/>

</listOfProducts>

<kineticLaw>

<math xmlns="<http://www.w3.org/1998/Math/MathML>">

<ci> FLUX_VALUE </ci>

</math>

<listOfParameters>

<parameter id="LOWER_BOUND" units="mmol_per_gDW_per_hr" value="-1000"/>

<parameter id="UPPER_BOUND" units="mmol_per_gDW_per_hr" value="1000"/>

<parameter id="FLUX_VALUE" units="mmol_per_gDW_per_hr" value="0"/>

<parameter id="OBJECTIVE_COEFFICIENT" units="dimensionless" value="0"/>

</listOfParameters>

</kineticLaw>

</reaction>

<reaction name="Resistance-Nodulation-Cell Division (RND) TCDB:2.A.60.1.5" id="R_r2251" metaid="_metaR_r2251" reversible="true" sboTerm="SBO:0000185">

<notes>

<body xmlns="<http://www.w3.org/1999/xhtml>">

<p>GENE_ASSOCIATION: </p>

<p>SUBSYSTEM: Transport, extracellular</p>

<p>EC Number: </p>

<p>Confidence Level: 0</p>

<p>AUTHORS: PMID:11076396,PMID:14579113</p>

<p>NOTES: </p>

</body>

</notes>

<listOfReactants>

<speciesReference species="M_HC02199_c"/>

<speciesReference species="M_hco3_c"/>

</listOfReactants>

<listOfProducts>

<speciesReference species="M_leuktrF4_c"/>

<speciesReference species="M_hco3_e"/>

</listOfProducts>

<kineticLaw>

<math xmlns="<http://www.w3.org/1998/Math/MathML>">

<ci> FLUX_VALUE </ci>

</math>

<listOfParameters>

<parameter id="LOWER_BOUND" units="mmol_per_gDW_per_hr" value="-1000"/>

<parameter id="UPPER_BOUND" units="mmol_per_gDW_per_hr" value="1000"/>

<parameter id="FLUX_VALUE" units="mmol_per_gDW_per_hr" value="0"/>

<parameter id="OBJECTIVE_COEFFICIENT" units="dimensionless" value="0"/>

</listOfParameters>

</kineticLaw>

</reaction>

<reaction name="Resistance-Nodulation-Cell Division (RND) TCDB:2.A.60.1.5" id="R_r2266" metaid="_metaR_r2266" reversible="true" sboTerm="SBO:0000185">

<notes>

<body xmlns="<http://www.w3.org/1999/xhtml>">

<p>GENE_ASSOCIATION: </p>

<p>SUBSYSTEM: Transport, extracellular</p>

<p>EC Number: </p>

<p>Confidence Level: 0</p>

<p>AUTHORS: PMID:11076396,PMID:14579113</p>

<p>NOTES: </p>

</body>

</notes>

<listOfReactants>

<speciesReference species="M_HC02199_c"/>

<speciesReference species="M_hco3_c"/>

</listOfReactants>

<listOfProducts>

<speciesReference species="M_HC02205_c"/>

<speciesReference species="M_hco3_e"/>

</listOfProducts>

<kineticLaw>

<math xmlns="<http://www.w3.org/1998/Math/MathML>">

<ci> FLUX_VALUE </ci>

</math>

<listOfParameters>

<parameter id="LOWER_BOUND" units="mmol_per_gDW_per_hr" value="-1000"/>

<parameter id="UPPER_BOUND" units="mmol_per_gDW_per_hr" value="1000"/>

<parameter id="FLUX_VALUE" units="mmol_per_gDW_per_hr" value="0"/>

<parameter id="OBJECTIVE_COEFFICIENT" units="dimensionless" value="0"/>

</listOfParameters>

</kineticLaw>

</reaction>

<reaction name="Resistance-Nodulation-Cell Division (RND) TCDB:2.A.60.1.5" id="R_r2275" metaid="_metaR_r2275" reversible="true" sboTerm="SBO:0000185">

<notes>

<body xmlns="<http://www.w3.org/1999/xhtml>">

<p>GENE_ASSOCIATION: </p>

<p>SUBSYSTEM: Transport, extracellular</p>

<p>EC Number: </p>

<p>Confidence Level: 0</p>

<p>AUTHORS: PMID:11076396,PMID:14579113</p>

<p>NOTES: </p>

</body>

</notes>

<listOfReactants>

<speciesReference species="M_HC02199_c"/>

<speciesReference species="M_hco3_c"/>

</listOfReactants>

<listOfProducts>

<speciesReference species="M_HC02208_c"/>

<speciesReference species="M_hco3_e"/>

</listOfProducts>

<kineticLaw>

<math xmlns="<http://www.w3.org/1998/Math/MathML>">

<ci> FLUX_VALUE </ci>

</math>

<listOfParameters>

<parameter id="LOWER_BOUND" units="mmol_per_gDW_per_hr" value="-1000"/>

<parameter id="UPPER_BOUND" units="mmol_per_gDW_per_hr" value="1000"/>

<parameter id="FLUX_VALUE" units="mmol_per_gDW_per_hr" value="0"/>

<parameter id="OBJECTIVE_COEFFICIENT" units="dimensionless" value="0"/>

</listOfParameters>

</kineticLaw>

</reaction>

<reaction name="Resistance-Nodulation-Cell Division (RND) TCDB:2.A.60.1.5" id="R_r2263" metaid="_metaR_r2263" reversible="true" sboTerm="SBO:0000185">

<notes>

<body xmlns="<http://www.w3.org/1999/xhtml>">

<p>GENE_ASSOCIATION: </p>

<p>SUBSYSTEM: Transport, extracellular</p>

<p>EC Number: </p>

<p>Confidence Level: 0</p>

<p>AUTHORS: PMID:11076396,PMID:14579113</p>

<p>NOTES: </p>

</body>

</notes>

<listOfReactants>

<speciesReference species="M_HC02199_c"/>

<speciesReference species="M_hco3_c"/>

</listOfReactants>

<listOfProducts>

<speciesReference species="M_HC02204_c"/>

<speciesReference species="M_hco3_e"/>

</listOfProducts>

<kineticLaw>

<math xmlns="<http://www.w3.org/1998/Math/MathML>">

<ci> FLUX_VALUE </ci>

</math>

<listOfParameters>

<parameter id="LOWER_BOUND" units="mmol_per_gDW_per_hr" value="-1000"/>

<parameter id="UPPER_BOUND" units="mmol_per_gDW_per_hr" value="1000"/>

<parameter id="FLUX_VALUE" units="mmol_per_gDW_per_hr" value="0"/>

<parameter id="OBJECTIVE_COEFFICIENT" units="dimensionless" value="0"/>

</listOfParameters>

</kineticLaw>

</reaction>

<reaction name="Resistance-Nodulation-Cell Division (RND) TCDB:2.A.60.1.5" id="R_r2290" metaid="_metaR_r2290" reversible="true" sboTerm="SBO:0000185">

<notes>

<body xmlns="<http://www.w3.org/1999/xhtml>">

<p>GENE_ASSOCIATION: </p>

<p>SUBSYSTEM: Transport, extracellular</p>

<p>EC Number: </p>

<p>Confidence Level: 0</p>

<p>AUTHORS: PMID:11076396,PMID:14579113</p>

<p>NOTES: </p>

</body>

</notes>

<listOfReactants>

<speciesReference species="M_HC02199_c"/>

<speciesReference species="M_hco3_c"/>

</listOfReactants>

<listOfProducts>

<speciesReference species="M_HC02214_c"/>

<speciesReference species="M_hco3_e"/>

</listOfProducts>

<kineticLaw>

<math xmlns="<http://www.w3.org/1998/Math/MathML>">

<ci> FLUX_VALUE </ci>

</math>

<listOfParameters>

<parameter id="LOWER_BOUND" units="mmol_per_gDW_per_hr" value="-1000"/>

<parameter id="UPPER_BOUND" units="mmol_per_gDW_per_hr" value="1000"/>

<parameter id="FLUX_VALUE" units="mmol_per_gDW_per_hr" value="0"/>

<parameter id="OBJECTIVE_COEFFICIENT" units="dimensionless" value="0"/>

</listOfParameters>

</kineticLaw>

</reaction>

<reaction name="Resistance-Nodulation-Cell Division (RND) TCDB:2.A.60.1.5" id="R_r2287" metaid="_metaR_r2287" reversible="true" sboTerm="SBO:0000185">

<notes>

<body xmlns="<http://www.w3.org/1999/xhtml>">

<p>GENE_ASSOCIATION: </p>

<p>SUBSYSTEM: Transport, extracellular</p>

<p>EC Number: </p>

<p>Confidence Level: 0</p>

<p>AUTHORS: PMID:11076396,PMID:14579113</p>

<p>NOTES: </p>

</body>

</notes>

<listOfReactants>

<speciesReference species="M_HC02199_c"/>

<speciesReference species="M_hco3_c"/>

</listOfReactants>

<listOfProducts>

<speciesReference species="M_HC02213_c"/>

<speciesReference species="M_hco3_e"/>

</listOfProducts>

<kineticLaw>

<math xmlns="<http://www.w3.org/1998/Math/MathML>">

<ci> FLUX_VALUE </ci>

</math>

<listOfParameters>

<parameter id="LOWER_BOUND" units="mmol_per_gDW_per_hr" value="-1000"/>

<parameter id="UPPER_BOUND" units="mmol_per_gDW_per_hr" value="1000"/>

<parameter id="FLUX_VALUE" units="mmol_per_gDW_per_hr" value="0"/>

<parameter id="OBJECTIVE_COEFFICIENT" units="dimensionless" value="0"/>

</listOfParameters>

</kineticLaw>

</reaction>

<reaction name="Resistance-Nodulation-Cell Division (RND) TCDB:2.A.60.1.5" id="R_r2296" metaid="_metaR_r2296" reversible="true" sboTerm="SBO:0000185">

<notes>

<body xmlns="<http://www.w3.org/1999/xhtml>">

<p>GENE_ASSOCIATION: </p>

<p>SUBSYSTEM: Transport, extracellular</p>

<p>EC Number: </p>

<p>Confidence Level: 0</p>

<p>AUTHORS: PMID:11076396,PMID:14579113</p>

<p>NOTES: </p>

</body>

</notes>

<listOfReactants>

<speciesReference species="M_HC02199_c"/>

<speciesReference species="M_hco3_c"/>

</listOfReactants>

<listOfProducts>

<speciesReference species="M_HC02216_c"/>

<speciesReference species="M_hco3_e"/>

</listOfProducts>

<kineticLaw>

<math xmlns="<http://www.w3.org/1998/Math/MathML>">

<ci> FLUX_VALUE </ci>

</math>

<listOfParameters>

<parameter id="LOWER_BOUND" units="mmol_per_gDW_per_hr" value="-1000"/>

<parameter id="UPPER_BOUND" units="mmol_per_gDW_per_hr" value="1000"/>

<parameter id="FLUX_VALUE" units="mmol_per_gDW_per_hr" value="0"/>

<parameter id="OBJECTIVE_COEFFICIENT" units="dimensionless" value="0"/>

</listOfParameters>

</kineticLaw>

</reaction>

<reaction name="Resistance-Nodulation-Cell Division (RND) TCDB:2.A.60.1.5" id="R_r2281" metaid="_metaR_r2281" reversible="true" sboTerm="SBO:0000185">

<notes>

<body xmlns="<http://www.w3.org/1999/xhtml>">

<p>GENE_ASSOCIATION: </p>

<p>SUBSYSTEM: Transport, extracellular</p>

<p>EC Number: </p>

<p>Confidence Level: 0</p>

<p>AUTHORS: PMID:11076396,PMID:14579113</p>

<p>NOTES: </p>

</body>

</notes>

<listOfReactants>

<speciesReference species="M_HC02199_c"/>

<speciesReference species="M_hco3_c"/>

</listOfReactants>

<listOfProducts>

<speciesReference species="M_HC02210_c"/>

<speciesReference species="M_hco3_e"/>

</listOfProducts>

<kineticLaw>

<math xmlns="<http://www.w3.org/1998/Math/MathML>">

<ci> FLUX_VALUE </ci>

</math>

<listOfParameters>

<parameter id="LOWER_BOUND" units="mmol_per_gDW_per_hr" value="-1000"/>

<parameter id="UPPER_BOUND" units="mmol_per_gDW_per_hr" value="1000"/>

<parameter id="FLUX_VALUE" units="mmol_per_gDW_per_hr" value="0"/>

<parameter id="OBJECTIVE_COEFFICIENT" units="dimensionless" value="0"/>

</listOfParameters>

</kineticLaw>

</reaction>

<reaction name="Resistance-Nodulation-Cell Division (RND) TCDB:2.A.60.1.5" id="R_r2299" metaid="_metaR_r2299" reversible="true" sboTerm="SBO:0000185">

<notes>

<body xmlns="<http://www.w3.org/1999/xhtml>">

<p>GENE_ASSOCIATION: </p>

<p>SUBSYSTEM: Transport, extracellular</p>

<p>EC Number: </p>

<p>Confidence Level: 0</p>

<p>AUTHORS: PMID:11076396,PMID:14579113</p>

<p>NOTES: </p>

</body>

</notes>

<listOfReactants>

<speciesReference species="M_HC02199_c"/>

<speciesReference species="M_hco3_c"/>

</listOfReactants>

<listOfProducts>

<speciesReference species="M_HC02217_c"/>

<speciesReference species="M_hco3_e"/>

</listOfProducts>

<kineticLaw>

<math xmlns="<http://www.w3.org/1998/Math/MathML>">

<ci> FLUX_VALUE </ci>

</math>

<listOfParameters>

<parameter id="LOWER_BOUND" units="mmol_per_gDW_per_hr" value="-1000"/>

<parameter id="UPPER_BOUND" units="mmol_per_gDW_per_hr" value="1000"/>

<parameter id="FLUX_VALUE" units="mmol_per_gDW_per_hr" value="0"/>

<parameter id="OBJECTIVE_COEFFICIENT" units="dimensionless" value="0"/>

</listOfParameters>

</kineticLaw>

</reaction>

<reaction name="Resistance-Nodulation-Cell Division (RND) TCDB:2.A.60.1.14" id="R_r2488" metaid="_metaR_r2488" reversible="true" sboTerm="SBO:0000185">

<notes>

<body xmlns="<http://www.w3.org/1999/xhtml>">

<p>GENE_ASSOCIATION: </p>

<p>SUBSYSTEM: Transport, extracellular</p>

<p>EC Number: </p>

<p>Confidence Level: 0</p>

<p>AUTHORS: PMID:14579113</p>

<p>NOTES: </p>

</body>

</notes>

<listOfReactants>

<speciesReference species="M_HC02199_e"/>

<speciesReference species="M_hco3_e"/>

</listOfReactants>

<listOfProducts>

<speciesReference species="M_HC02199_c"/>

<speciesReference species="M_hco3_c"/>

</listOfProducts>

<kineticLaw>

<math xmlns="<http://www.w3.org/1998/Math/MathML>">

<ci> FLUX_VALUE </ci>

</math>

<listOfParameters>

<parameter id="LOWER_BOUND" units="mmol_per_gDW_per_hr" value="-1000"/>

<parameter id="UPPER_BOUND" units="mmol_per_gDW_per_hr" value="1000"/>

<parameter id="FLUX_VALUE" units="mmol_per_gDW_per_hr" value="0"/>

<parameter id="OBJECTIVE_COEFFICIENT" units="dimensionless" value="0"/>

</listOfParameters>

</kineticLaw>

</reaction>

<reaction name="Resistance-Nodulation-Cell Division (RND) TCDB:2.A.60.1.14" id="R_r2482" metaid="_metaR_r2482" reversible="true" sboTerm="SBO:0000185">

<notes>

<body xmlns="<http://www.w3.org/1999/xhtml>">

<p>GENE_ASSOCIATION: </p>

<p>SUBSYSTEM: Transport, extracellular</p>

<p>EC Number: </p>

<p>Confidence Level: 0</p>

<p>AUTHORS: PMID:14579113</p>

<p>NOTES: </p>

</body>

</notes>

<listOfReactants>

<speciesReference species="M_HC02199_e"/>

<speciesReference species="M_hco3_e"/>

</listOfReactants>

<listOfProducts>

<speciesReference species="M_HC02199_c"/>

<speciesReference species="M_hco3_c"/>

</listOfProducts>

<kineticLaw>

<math xmlns="<http://www.w3.org/1998/Math/MathML>">

<ci> FLUX_VALUE </ci>

</math>

<listOfParameters>

<parameter id="LOWER_BOUND" units="mmol_per_gDW_per_hr" value="-1000"/>

<parameter id="UPPER_BOUND" units="mmol_per_gDW_per_hr" value="1000"/>

<parameter id="FLUX_VALUE" units="mmol_per_gDW_per_hr" value="0"/>

<parameter id="OBJECTIVE_COEFFICIENT" units="dimensionless" value="0"/>

</listOfParameters>

</kineticLaw>

</reaction>

<reaction name="Resistance-Nodulation-Cell Division (RND) TCDB:2.A.60.1.14" id="R_r2494" metaid="_metaR_r2494" reversible="true" sboTerm="SBO:0000185">

<notes>

<body xmlns="<http://www.w3.org/1999/xhtml>">

<p>GENE_ASSOCIATION: </p>

<p>SUBSYSTEM: Transport, extracellular</p>

<p>EC Number: </p>

<p>Confidence Level: 0</p>

<p>AUTHORS: PMID:14579113</p>

<p>NOTES: </p>

</body>

</notes>

<listOfReactants>

<speciesReference species="M_HC02199_e"/>

<speciesReference species="M_hco3_e"/>

</listOfReactants>

<listOfProducts>

<speciesReference species="M_HC02199_c"/>

<speciesReference species="M_hco3_c"/>

</listOfProducts>

<kineticLaw>

<math xmlns="<http://www.w3.org/1998/Math/MathML>">

<ci> FLUX_VALUE </ci>

</math>

<listOfParameters>

<parameter id="LOWER_BOUND" units="mmol_per_gDW_per_hr" value="-1000"/>

<parameter id="UPPER_BOUND" units="mmol_per_gDW_per_hr" value="1000"/>

<parameter id="FLUX_VALUE" units="mmol_per_gDW_per_hr" value="0"/>

<parameter id="OBJECTIVE_COEFFICIENT" units="dimensionless" value="0"/>

</listOfParameters>

</kineticLaw>

</reaction>

<reaction name="Resistance-Nodulation-Cell Division (RND) TCDB:2.A.60.1.14" id="R_r2491" metaid="_metaR_r2491" reversible="true" sboTerm="SBO:0000185">

<notes>

<body xmlns="<http://www.w3.org/1999/xhtml>">

<p>GENE_ASSOCIATION: </p>

<p>SUBSYSTEM: Transport, extracellular</p>

<p>EC Number: </p>

<p>Confidence Level: 0</p>

<p>AUTHORS: PMID:14579113</p>

<p>NOTES: </p>

</body>

</notes>

<listOfReactants>

<speciesReference species="M_hco3_e"/>

<speciesReference species="M_HC02199_e"/>

</listOfReactants>

<listOfProducts>

<speciesReference species="M_HC02199_c"/>

<speciesReference species="M_hco3_c"/>

</listOfProducts>

<kineticLaw>

<math xmlns="<http://www.w3.org/1998/Math/MathML>">

<ci> FLUX_VALUE </ci>

</math>

<listOfParameters>

<parameter id="LOWER_BOUND" units="mmol_per_gDW_per_hr" value="-1000"/>

<parameter id="UPPER_BOUND" units="mmol_per_gDW_per_hr" value="1000"/>

<parameter id="FLUX_VALUE" units="mmol_per_gDW_per_hr" value="0"/>

<parameter id="OBJECTIVE_COEFFICIENT" units="dimensionless" value="0"/>

</listOfParameters>

</kineticLaw>

</reaction>

<reaction name="Resistance-Nodulation-Cell Division (RND) TCDB:2.A.60.1.14" id="R_r2170" metaid="_metaR_r2170" reversible="true" sboTerm="SBO:0000185">

<notes>

<body xmlns="<http://www.w3.org/1999/xhtml>">

<p>GENE_ASSOCIATION: </p>

<p>SUBSYSTEM: Transport, extracellular</p>

<p>EC Number: </p>

<p>Confidence Level: 0</p>

<p>AUTHORS: PMID:11076394,PMID:11076396,PMID:14579113</p>

<p>NOTES: </p>

</body>

</notes>

<listOfReactants>

<speciesReference species="M_HC02200_c"/>

<speciesReference species="M_hco3_c"/>

</listOfReactants>

<listOfProducts>

<speciesReference species="M_HC02192_c"/>

<speciesReference species="M_hco3_e"/>

</listOfProducts>

<kineticLaw>

<math xmlns="<http://www.w3.org/1998/Math/MathML>">

<ci> FLUX_VALUE </ci>

</math>

<listOfParameters>

<parameter id="LOWER_BOUND" units="mmol_per_gDW_per_hr" value="-1000"/>

<parameter id="UPPER_BOUND" units="mmol_per_gDW_per_hr" value="1000"/>

<parameter id="FLUX_VALUE" units="mmol_per_gDW_per_hr" value="0"/>

<parameter id="OBJECTIVE_COEFFICIENT" units="dimensionless" value="0"/>

</listOfParameters>

</kineticLaw>

</reaction>

<reaction name="Resistance-Nodulation-Cell Division (RND) TCDB:2.A.60.1.14" id="R_r2486" metaid="_metaR_r2486" reversible="true" sboTerm="SBO:0000185">

<notes>

<body xmlns="<http://www.w3.org/1999/xhtml>">

<p>GENE_ASSOCIATION: </p>

<p>SUBSYSTEM: Transport, extracellular</p>

<p>EC Number: </p>

<p>Confidence Level: 0</p>

<p>AUTHORS: PMID:14579113</p>

<p>NOTES: </p>

</body>

</notes>

<listOfReactants>

<speciesReference species="M_hco3_e"/>

<speciesReference species="M_HC02200_e"/>

</listOfReactants>

<listOfProducts>

<speciesReference species="M_HC02200_c"/>

<speciesReference species="M_hco3_c"/>

</listOfProducts>

<kineticLaw>

<math xmlns="<http://www.w3.org/1998/Math/MathML>">

<ci> FLUX_VALUE </ci>

</math>

<listOfParameters>

<parameter id="LOWER_BOUND" units="mmol_per_gDW_per_hr" value="-1000"/>

<parameter id="UPPER_BOUND" units="mmol_per_gDW_per_hr" value="1000"/>

<parameter id="FLUX_VALUE" units="mmol_per_gDW_per_hr" value="0"/>

<parameter id="OBJECTIVE_COEFFICIENT" units="dimensionless" value="0"/>

</listOfParameters>

</kineticLaw>

</reaction>

<reaction name="Resistance-Nodulation-Cell Division (RND) TCDB:2.A.60.1.14" id="R_r2173" metaid="_metaR_r2173" reversible="true" sboTerm="SBO:0000185">

<notes>

<body xmlns="<http://www.w3.org/1999/xhtml>">

<p>GENE_ASSOCIATION: </p>

<p>SUBSYSTEM: Transport, extracellular</p>

<p>EC Number: </p>

<p>Confidence Level: 0</p>

<p>AUTHORS: PMID:11076394,PMID:11076396,PMID:14579113</p>

<p>NOTES: </p>

</body>

</notes>

<listOfReactants>

<speciesReference species="M_HC02200_c"/>

<speciesReference species="M_hco3_c"/>

</listOfReactants>

<listOfProducts>

<speciesReference species="M_HC02193_c"/>

<speciesReference species="M_hco3_e"/>

</listOfProducts>

<kineticLaw>

<math xmlns="<http://www.w3.org/1998/Math/MathML>">

<ci> FLUX_VALUE </ci>

</math>

<listOfParameters>

<parameter id="LOWER_BOUND" units="mmol_per_gDW_per_hr" value="-1000"/>

<parameter id="UPPER_BOUND" units="mmol_per_gDW_per_hr" value="1000"/>

<parameter id="FLUX_VALUE" units="mmol_per_gDW_per_hr" value="0"/>

<parameter id="OBJECTIVE_COEFFICIENT" units="dimensionless" value="0"/>

</listOfParameters>

</kineticLaw>

</reaction>

<reaction name="Resistance-Nodulation-Cell Division (RND) TCDB:2.A.60.1.14" id="R_r2176" metaid="_metaR_r2176" reversible="true" sboTerm="SBO:0000185">

<notes>

<body xmlns="<http://www.w3.org/1999/xhtml>">

<p>GENE_ASSOCIATION: </p>

<p>SUBSYSTEM: Transport, extracellular</p>

<p>EC Number: </p>

<p>Confidence Level: 0</p>

<p>AUTHORS: PMID:11076394,PMID:11076396,PMID:14579113</p>

<p>NOTES: </p>

</body>

</notes>

<listOfReactants>

<speciesReference species="M_HC02200_c"/>

<speciesReference species="M_hco3_c"/>

</listOfReactants>

<listOfProducts>

<speciesReference species="M_HC02194_c"/>

<speciesReference species="M_hco3_e"/>

</listOfProducts>

<kineticLaw>

<math xmlns="<http://www.w3.org/1998/Math/MathML>">

<ci> FLUX_VALUE </ci>

</math>

<listOfParameters>

<parameter id="LOWER_BOUND" units="mmol_per_gDW_per_hr" value="-1000"/>

<parameter id="UPPER_BOUND" units="mmol_per_gDW_per_hr" value="1000"/>

<parameter id="FLUX_VALUE" units="mmol_per_gDW_per_hr" value="0"/>

<parameter id="OBJECTIVE_COEFFICIENT" units="dimensionless" value="0"/>

</listOfParameters>

</kineticLaw>

</reaction>

<reaction name="Resistance-Nodulation-Cell Division (RND) TCDB:2.A.60.1.14" id="R_r2185" metaid="_metaR_r2185" reversible="true" sboTerm="SBO:0000185">

<notes>

<body xmlns="<http://www.w3.org/1999/xhtml>">

<p>GENE_ASSOCIATION: 6566.1</p>

<p>SUBSYSTEM: Transport, extracellular</p>

<p>EC Number: </p>

<p>Confidence Level: 0</p>

<p>AUTHORS: PMID:11076394,PMID:11076396,PMID:14579113</p>

<p>NOTES: </p>

</body>

</notes>

<listOfReactants>

<speciesReference species="M_HC02200_c"/>

<speciesReference species="M_hco3_c"/>

</listOfReactants>

<listOfProducts>

<speciesReference species="M_HC02197_c"/>

<speciesReference species="M_hco3_e"/>

</listOfProducts>

<kineticLaw>

<math xmlns="<http://www.w3.org/1998/Math/MathML>">

<ci> FLUX_VALUE </ci>

</math>

<listOfParameters>

<parameter id="LOWER_BOUND" units="mmol_per_gDW_per_hr" value="-1000"/>

<parameter id="UPPER_BOUND" units="mmol_per_gDW_per_hr" value="1000"/>

<parameter id="FLUX_VALUE" units="mmol_per_gDW_per_hr" value="0"/>

<parameter id="OBJECTIVE_COEFFICIENT" units="dimensionless" value="0"/>

</listOfParameters>

</kineticLaw>

</reaction>

<reaction name="Resistance-Nodulation-Cell Division (RND) TCDB:2.A.60.1.14" id="R_r2182" metaid="_metaR_r2182" reversible="true" sboTerm="SBO:0000185">

<notes>

<body xmlns="<http://www.w3.org/1999/xhtml>">

<p>GENE_ASSOCIATION: 6566.1</p>

<p>SUBSYSTEM: Transport, extracellular</p>

<p>EC Number: </p>

<p>Confidence Level: 0</p>

<p>AUTHORS: PMID:11076394,PMID:11076396,PMID:14579113</p>

<p>NOTES: </p>

</body>

</notes>

<listOfReactants>

<speciesReference species="M_HC02200_c"/>

<speciesReference species="M_hco3_c"/>

</listOfReactants>

<listOfProducts>

<speciesReference species="M_HC02196_c"/>

<speciesReference species="M_hco3_e"/>

</listOfProducts>

<kineticLaw>

<math xmlns="<http://www.w3.org/1998/Math/MathML>">

<ci> FLUX_VALUE </ci>

</math>

<listOfParameters>

<parameter id="LOWER_BOUND" units="mmol_per_gDW_per_hr" value="-1000"/>

<parameter id="UPPER_BOUND" units="mmol_per_gDW_per_hr" value="1000"/>

<parameter id="FLUX_VALUE" units="mmol_per_gDW_per_hr" value="0"/>

<parameter id="OBJECTIVE_COEFFICIENT" units="dimensionless" value="0"/>

</listOfParameters>

</kineticLaw>

</reaction>

<reaction name="Resistance-Nodulation-Cell Division (RND) TCDB:2.A.60.1.14" id="R_r2179" metaid="_metaR_r2179" reversible="true" sboTerm="SBO:0000185">

<notes>

<body xmlns="<http://www.w3.org/1999/xhtml>">

<p>GENE_ASSOCIATION: 6566.1</p>

<p>SUBSYSTEM: Transport, extracellular</p>

<p>EC Number: </p>

<p>Confidence Level: 0</p>

<p>AUTHORS: PMID:11076394,PMID:11076396,PMID:14579113</p>

<p>NOTES: </p>

</body>

</notes>

<listOfReactants>

<speciesReference species="M_HC02200_c"/>

<speciesReference species="M_hco3_c"/>

</listOfReactants>

<listOfProducts>

<speciesReference species="M_HC02195_c"/>

<speciesReference species="M_hco3_e"/>

</listOfProducts>

<kineticLaw>

<math xmlns="<http://www.w3.org/1998/Math/MathML>">

<ci> FLUX_VALUE </ci>

</math>

<listOfParameters>

<parameter id="LOWER_BOUND" units="mmol_per_gDW_per_hr" value="-1000"/>

<parameter id="UPPER_BOUND" units="mmol_per_gDW_per_hr" value="1000"/>

<parameter id="FLUX_VALUE" units="mmol_per_gDW_per_hr" value="0"/>

<parameter id="OBJECTIVE_COEFFICIENT" units="dimensionless" value="0"/>

</listOfParameters>

</kineticLaw>

</reaction>

<reaction name="Resistance-Nodulation-Cell Division (RND) TCDB:2.A.60.1.14" id="R_r2191" metaid="_metaR_r2191" reversible="true" sboTerm="SBO:0000185">

<notes>

<body xmlns="<http://www.w3.org/1999/xhtml>">

<p>GENE_ASSOCIATION: 6566.1</p>

<p>SUBSYSTEM: Transport, extracellular</p>

<p>EC Number: </p>

<p>Confidence Level: 0</p>

<p>AUTHORS: PMID:11076394,PMID:11076396,PMID:14579113</p>

<p>NOTES: </p>

</body>

</notes>

<listOfReactants>

<speciesReference species="M_HC02200_c"/>

<speciesReference species="M_hco3_c"/>

</listOfReactants>

<listOfProducts>

<speciesReference species="M_HC02220_c"/>

<speciesReference species="M_hco3_e"/>

</listOfProducts>

<kineticLaw>

<math xmlns="<http://www.w3.org/1998/Math/MathML>">

<ci> FLUX_VALUE </ci>

</math>

<listOfParameters>

<parameter id="LOWER_BOUND" units="mmol_per_gDW_per_hr" value="-1000"/>

<parameter id="UPPER_BOUND" units="mmol_per_gDW_per_hr" value="1000"/>

<parameter id="FLUX_VALUE" units="mmol_per_gDW_per_hr" value="0"/>

<parameter id="OBJECTIVE_COEFFICIENT" units="dimensionless" value="0"/>

</listOfParameters>

</kineticLaw>

</reaction>

<reaction name="Resistance-Nodulation-Cell Division (RND) TCDB:2.A.60.1.14" id="R_r2213" metaid="_metaR_r2213" reversible="true" sboTerm="SBO:0000185">

<notes>

<body xmlns="<http://www.w3.org/1999/xhtml>">

<p>GENE_ASSOCIATION: 6566.1</p>

<p>SUBSYSTEM: Transport, extracellular</p>

<p>EC Number: </p>

<p>Confidence Level: 0</p>

<p>AUTHORS: PMID:11076396,PMID:14579113</p>

<p>NOTES: </p>

</body>

</notes>

<listOfReactants>

<speciesReference species="M_HC02200_c"/>

<speciesReference species="M_hco3_c"/>

</listOfReactants>

<listOfProducts>

<speciesReference species="M_HC02187_c"/>

<speciesReference species="M_hco3_e"/>

</listOfProducts>

<kineticLaw>

<math xmlns="<http://www.w3.org/1998/Math/MathML>">

<ci> FLUX_VALUE </ci>

</math>

<listOfParameters>

<parameter id="LOWER_BOUND" units="mmol_per_gDW_per_hr" value="-1000"/>

<parameter id="UPPER_BOUND" units="mmol_per_gDW_per_hr" value="1000"/>

<parameter id="FLUX_VALUE" units="mmol_per_gDW_per_hr" value="0"/>

<parameter id="OBJECTIVE_COEFFICIENT" units="dimensionless" value="0"/>

</listOfParameters>

</kineticLaw>

</reaction>

<reaction name="Resistance-Nodulation-Cell Division (RND) TCDB:2.A.60.1.5" id="R_r2240" metaid="_metaR_r2240" reversible="true" sboTerm="SBO:0000185">

<notes>

<body xmlns="<http://www.w3.org/1999/xhtml>">

<p>GENE_ASSOCIATION: </p>

<p>SUBSYSTEM: Transport, extracellular</p>

<p>EC Number: </p>

<p>Confidence Level: 0</p>

<p>AUTHORS: PMID:11076396,PMID:14579113</p>

<p>NOTES: </p>

</body>

</notes>

<listOfReactants>

<speciesReference species="M_HC02200_c"/>

<speciesReference species="M_hco3_c"/>

</listOfReactants>

<listOfProducts>

<speciesReference species="M_HC02180_c"/>

<speciesReference species="M_hco3_e"/>

</listOfProducts>

<kineticLaw>

<math xmlns="<http://www.w3.org/1998/Math/MathML>">

<ci> FLUX_VALUE </ci>

</math>

<listOfParameters>

<parameter id="LOWER_BOUND" units="mmol_per_gDW_per_hr" value="-1000"/>

<parameter id="UPPER_BOUND" units="mmol_per_gDW_per_hr" value="1000"/>

<parameter id="FLUX_VALUE" units="mmol_per_gDW_per_hr" value="0"/>

<parameter id="OBJECTIVE_COEFFICIENT" units="dimensionless" value="0"/>

</listOfParameters>

</kineticLaw>

</reaction>

<reaction name="Resistance-Nodulation-Cell Division (RND) TCDB:2.A.60.1.5" id="R_r2237" metaid="_metaR_r2237" reversible="true" sboTerm="SBO:0000185">

<notes>

<body xmlns="<http://www.w3.org/1999/xhtml>">

<p>GENE_ASSOCIATION: </p>

<p>SUBSYSTEM: Transport, extracellular</p>

<p>EC Number: </p>

<p>Confidence Level: 0</p>

<p>AUTHORS: PMID:11076396,PMID:14579113</p>

<p>NOTES: </p>

</body>

</notes>

<listOfReactants>

<speciesReference species="M_HC02200_c"/>

<speciesReference species="M_hco3_c"/>

</listOfReactants>

<listOfProducts>

<speciesReference species="M_HC02179_c"/>

<speciesReference species="M_hco3_e"/>

</listOfProducts>

<kineticLaw>

<math xmlns="<http://www.w3.org/1998/Math/MathML>">

<ci> FLUX_VALUE </ci>

</math>

<listOfParameters>

<parameter id="LOWER_BOUND" units="mmol_per_gDW_per_hr" value="-1000"/>

<parameter id="UPPER_BOUND" units="mmol_per_gDW_per_hr" value="1000"/>

<parameter id="FLUX_VALUE" units="mmol_per_gDW_per_hr" value="0"/>

<parameter id="OBJECTIVE_COEFFICIENT" units="dimensionless" value="0"/>

</listOfParameters>

</kineticLaw>

</reaction>

<reaction name="Resistance-Nodulation-Cell Division (RND) TCDB:2.A.60.1.5" id="R_r2252" metaid="_metaR_r2252" reversible="true" sboTerm="SBO:0000185">

<notes>

<body xmlns="<http://www.w3.org/1999/xhtml>">

<p>GENE_ASSOCIATION: </p>

<p>SUBSYSTEM: Transport, extracellular</p>

<p>EC Number: </p>

<p>Confidence Level: 0</p>

<p>AUTHORS: PMID:11076396,PMID:14579113</p>

<p>NOTES: </p>

</body>

</notes>

<listOfReactants>

<speciesReference species="M_HC02200_c"/>

<speciesReference species="M_hco3_c"/>

</listOfReactants>

<listOfProducts>

<speciesReference species="M_leuktrF4_c"/>

<speciesReference species="M_hco3_e"/>

</listOfProducts>

<kineticLaw>

<math xmlns="<http://www.w3.org/1998/Math/MathML>">

<ci> FLUX_VALUE </ci>

</math>

<listOfParameters>

<parameter id="LOWER_BOUND" units="mmol_per_gDW_per_hr" value="-1000"/>

<parameter id="UPPER_BOUND" units="mmol_per_gDW_per_hr" value="1000"/>

<parameter id="FLUX_VALUE" units="mmol_per_gDW_per_hr" value="0"/>

<parameter id="OBJECTIVE_COEFFICIENT" units="dimensionless" value="0"/>

</listOfParameters>

</kineticLaw>

</reaction>

<reaction name="Resistance-Nodulation-Cell Division (RND) TCDB:2.A.60.1.5" id="R_r2267" metaid="_metaR_r2267" reversible="true" sboTerm="SBO:0000185">

<notes>

<body xmlns="<http://www.w3.org/1999/xhtml>">

<p>GENE_ASSOCIATION: </p>

<p>SUBSYSTEM: Transport, extracellular</p>

<p>EC Number: </p>

<p>Confidence Level: 0</p>

<p>AUTHORS: PMID:11076396,PMID:14579113</p>

<p>NOTES: </p>

</body>

</notes>

<listOfReactants>

<speciesReference species="M_HC02200_c"/>

<speciesReference species="M_hco3_c"/>

</listOfReactants>

<listOfProducts>

<speciesReference species="M_HC02205_c"/>

<speciesReference species="M_hco3_e"/>

</listOfProducts>

<kineticLaw>

<math xmlns="<http://www.w3.org/1998/Math/MathML>">

<ci> FLUX_VALUE </ci>

</math>

<listOfParameters>

<parameter id="LOWER_BOUND" units="mmol_per_gDW_per_hr" value="-1000"/>

<parameter id="UPPER_BOUND" units="mmol_per_gDW_per_hr" value="1000"/>

<parameter id="FLUX_VALUE" units="mmol_per_gDW_per_hr" value="0"/>

<parameter id="OBJECTIVE_COEFFICIENT" units="dimensionless" value="0"/>

</listOfParameters>

</kineticLaw>

</reaction>

<reaction name="Resistance-Nodulation-Cell Division (RND) TCDB:2.A.60.1.5" id="R_r2276" metaid="_metaR_r2276" reversible="true" sboTerm="SBO:0000185">

<notes>

<body xmlns="<http://www.w3.org/1999/xhtml>">

<p>GENE_ASSOCIATION: </p>

<p>SUBSYSTEM: Transport, extracellular</p>

<p>EC Number: </p>

<p>Confidence Level: 0</p>

<p>AUTHORS: PMID:11076396,PMID:14579113</p>

<p>NOTES: </p>

</body>

</notes>

<listOfReactants>

<speciesReference species="M_HC02200_c"/>

<speciesReference species="M_hco3_c"/>

</listOfReactants>

<listOfProducts>

<speciesReference species="M_HC02208_c"/>

<speciesReference species="M_hco3_e"/>

</listOfProducts>

<kineticLaw>

<math xmlns="<http://www.w3.org/1998/Math/MathML>">

<ci> FLUX_VALUE </ci>

</math>

<listOfParameters>

<parameter id="LOWER_BOUND" units="mmol_per_gDW_per_hr" value="-1000"/>

<parameter id="UPPER_BOUND" units="mmol_per_gDW_per_hr" value="1000"/>

<parameter id="FLUX_VALUE" units="mmol_per_gDW_per_hr" value="0"/>

<parameter id="OBJECTIVE_COEFFICIENT" units="dimensionless" value="0"/>

</listOfParameters>

</kineticLaw>

</reaction>

<reaction name="Resistance-Nodulation-Cell Division (RND) TCDB:2.A.60.1.5" id="R_r2264" metaid="_metaR_r2264" reversible="true" sboTerm="SBO:0000185">

<notes>

<body xmlns="<http://www.w3.org/1999/xhtml>">

<p>GENE_ASSOCIATION: </p>

<p>SUBSYSTEM: Transport, extracellular</p>

<p>EC Number: </p>

<p>Confidence Level: 0</p>

<p>AUTHORS: PMID:11076396,PMID:14579113</p>

<p>NOTES: </p>

</body>

</notes>

<listOfReactants>

<speciesReference species="M_HC02200_c"/>

<speciesReference species="M_hco3_c"/>

</listOfReactants>

<listOfProducts>

<speciesReference species="M_HC02204_c"/>

<speciesReference species="M_hco3_e"/>

</listOfProducts>

<kineticLaw>

<math xmlns="<http://www.w3.org/1998/Math/MathML>">

<ci> FLUX_VALUE </ci>

</math>

<listOfParameters>

<parameter id="LOWER_BOUND" units="mmol_per_gDW_per_hr" value="-1000"/>

<parameter id="UPPER_BOUND" units="mmol_per_gDW_per_hr" value="1000"/>

<parameter id="FLUX_VALUE" units="mmol_per_gDW_per_hr" value="0"/>

<parameter id="OBJECTIVE_COEFFICIENT" units="dimensionless" value="0"/>

</listOfParameters>

</kineticLaw>

</reaction>

<reaction name="Resistance-Nodulation-Cell Division (RND) TCDB:2.A.60.1.5" id="R_r2291" metaid="_metaR_r2291" reversible="true" sboTerm="SBO:0000185">

<notes>

<body xmlns="<http://www.w3.org/1999/xhtml>">

<p>GENE_ASSOCIATION: </p>

<p>SUBSYSTEM: Transport, extracellular</p>

<p>EC Number: </p>

<p>Confidence Level: 0</p>

<p>AUTHORS: PMID:11076396,PMID:14579113</p>

<p>NOTES: </p>

</body>

</notes>

<listOfReactants>

<speciesReference species="M_HC02200_c"/>

<speciesReference species="M_hco3_c"/>

</listOfReactants>

<listOfProducts>

<speciesReference species="M_HC02214_c"/>

<speciesReference species="M_hco3_e"/>

</listOfProducts>

<kineticLaw>

<math xmlns="<http://www.w3.org/1998/Math/MathML>">

<ci> FLUX_VALUE </ci>

</math>

<listOfParameters>

<parameter id="LOWER_BOUND" units="mmol_per_gDW_per_hr" value="-1000"/>

<parameter id="UPPER_BOUND" units="mmol_per_gDW_per_hr" value="1000"/>

<parameter id="FLUX_VALUE" units="mmol_per_gDW_per_hr" value="0"/>

<parameter id="OBJECTIVE_COEFFICIENT" units="dimensionless" value="0"/>

</listOfParameters>

</kineticLaw>

</reaction>

<reaction name="Resistance-Nodulation-Cell Division (RND) TCDB:2.A.60.1.5" id="R_r2288" metaid="_metaR_r2288" reversible="true" sboTerm="SBO:0000185">

<notes>

<body xmlns="<http://www.w3.org/1999/xhtml>">

<p>GENE_ASSOCIATION: </p>

<p>SUBSYSTEM: Transport, extracellular</p>

<p>EC Number: </p>

<p>Confidence Level: 0</p>

<p>AUTHORS: PMID:11076396,PMID:14579113</p>

<p>NOTES: </p>

</body>

</notes>

<listOfReactants>

<speciesReference species="M_HC02200_c"/>

<speciesReference species="M_hco3_c"/>

</listOfReactants>

<listOfProducts>

<speciesReference species="M_HC02213_c"/>

<speciesReference species="M_hco3_e"/>

</listOfProducts>

<kineticLaw>

<math xmlns="<http://www.w3.org/1998/Math/MathML>">

<ci> FLUX_VALUE </ci>

</math>

<listOfParameters>

<parameter id="LOWER_BOUND" units="mmol_per_gDW_per_hr" value="-1000"/>

<parameter id="UPPER_BOUND" units="mmol_per_gDW_per_hr" value="1000"/>

<parameter id="FLUX_VALUE" units="mmol_per_gDW_per_hr" value="0"/>

<parameter id="OBJECTIVE_COEFFICIENT" units="dimensionless" value="0"/>

</listOfParameters>

</kineticLaw>

</reaction>

<reaction name="Resistance-Nodulation-Cell Division (RND) TCDB:2.A.60.1.5" id="R_r2297" metaid="_metaR_r2297" reversible="true" sboTerm="SBO:0000185">

<notes>

<body xmlns="<http://www.w3.org/1999/xhtml>">

<p>GENE_ASSOCIATION: </p>

<p>SUBSYSTEM: Transport, extracellular</p>

<p>EC Number: </p>

<p>Confidence Level: 0</p>

<p>AUTHORS: PMID:11076396,PMID:14579113</p>

<p>NOTES: </p>

</body>

</notes>

<listOfReactants>

<speciesReference species="M_HC02200_c"/>

<speciesReference species="M_hco3_c"/>

</listOfReactants>

<listOfProducts>

<speciesReference species="M_HC02216_c"/>

<speciesReference species="M_hco3_e"/>

</listOfProducts>

<kineticLaw>

<math xmlns="<http://www.w3.org/1998/Math/MathML>">

<ci> FLUX_VALUE </ci>

</math>

<listOfParameters>

<parameter id="LOWER_BOUND" units="mmol_per_gDW_per_hr" value="-1000"/>

<parameter id="UPPER_BOUND" units="mmol_per_gDW_per_hr" value="1000"/>

<parameter id="FLUX_VALUE" units="mmol_per_gDW_per_hr" value="0"/>

<parameter id="OBJECTIVE_COEFFICIENT" units="dimensionless" value="0"/>

</listOfParameters>

</kineticLaw>

</reaction>

<reaction name="Resistance-Nodulation-Cell Division (RND) TCDB:2.A.60.1.5" id="R_r2282" metaid="_metaR_r2282" reversible="true" sboTerm="SBO:0000185">

<notes>

<body xmlns="<http://www.w3.org/1999/xhtml>">

<p>GENE_ASSOCIATION: </p>

<p>SUBSYSTEM: Transport, extracellular</p>

<p>EC Number: </p>

<p>Confidence Level: 0</p>

<p>AUTHORS: PMID:11076396,PMID:14579113</p>

<p>NOTES: </p>

</body>

</notes>

<listOfReactants>

<speciesReference species="M_HC02200_c"/>

<speciesReference species="M_hco3_c"/>

</listOfReactants>

<listOfProducts>

<speciesReference species="M_HC02210_c"/>

<speciesReference species="M_hco3_e"/>

</listOfProducts>

<kineticLaw>

<math xmlns="<http://www.w3.org/1998/Math/MathML>">

<ci> FLUX_VALUE </ci>

</math>

<listOfParameters>

<parameter id="LOWER_BOUND" units="mmol_per_gDW_per_hr" value="-1000"/>

<parameter id="UPPER_BOUND" units="mmol_per_gDW_per_hr" value="1000"/>

<parameter id="FLUX_VALUE" units="mmol_per_gDW_per_hr" value="0"/>

<parameter id="OBJECTIVE_COEFFICIENT" units="dimensionless" value="0"/>

</listOfParameters>

</kineticLaw>

</reaction>

<reaction name="Resistance-Nodulation-Cell Division (RND) TCDB:2.A.60.1.5" id="R_r2300" metaid="_metaR_r2300" reversible="true" sboTerm="SBO:0000185">

<notes>

<body xmlns="<http://www.w3.org/1999/xhtml>">

<p>GENE_ASSOCIATION: </p>

<p>SUBSYSTEM: Transport, extracellular</p>

<p>EC Number: </p>

<p>Confidence Level: 0</p>

<p>AUTHORS: PMID:11076396,PMID:14579113</p>

<p>NOTES: </p>

</body>

</notes>

<listOfReactants>

<speciesReference species="M_HC02200_c"/>

<speciesReference species="M_hco3_c"/>

</listOfReactants>

<listOfProducts>

<speciesReference species="M_HC02217_c"/>

<speciesReference species="M_hco3_e"/>

</listOfProducts>

<kineticLaw>

<math xmlns="<http://www.w3.org/1998/Math/MathML>">

<ci> FLUX_VALUE </ci>

</math>

<listOfParameters>

<parameter id="LOWER_BOUND" units="mmol_per_gDW_per_hr" value="-1000"/>

<parameter id="UPPER_BOUND" units="mmol_per_gDW_per_hr" value="1000"/>

<parameter id="FLUX_VALUE" units="mmol_per_gDW_per_hr" value="0"/>

<parameter id="OBJECTIVE_COEFFICIENT" units="dimensionless" value="0"/>

</listOfParameters>

</kineticLaw>

</reaction>

<reaction name="Resistance-Nodulation-Cell Division (RND) TCDB:2.A.60.1.14" id="R_r2483" metaid="_metaR_r2483" reversible="true" sboTerm="SBO:0000185">

<notes>

<body xmlns="<http://www.w3.org/1999/xhtml>">

<p>GENE_ASSOCIATION: </p>

<p>SUBSYSTEM: Transport, extracellular</p>

<p>EC Number: </p>

<p>Confidence Level: 0</p>

<p>AUTHORS: PMID:14579113</p>

<p>NOTES: </p>

</body>

</notes>

<listOfReactants>

<speciesReference species="M_hco3_e"/>

<speciesReference species="M_HC02200_e"/>

</listOfReactants>

<listOfProducts>

<speciesReference species="M_HC02200_c"/>

<speciesReference species="M_hco3_c"/>

</listOfProducts>

<kineticLaw>

<math xmlns="<http://www.w3.org/1998/Math/MathML>">

<ci> FLUX_VALUE </ci>

</math>

<listOfParameters>

<parameter id="LOWER_BOUND" units="mmol_per_gDW_per_hr" value="-1000"/>

<parameter id="UPPER_BOUND" units="mmol_per_gDW_per_hr" value="1000"/>

<parameter id="FLUX_VALUE" units="mmol_per_gDW_per_hr" value="0"/>

<parameter id="OBJECTIVE_COEFFICIENT" units="dimensionless" value="0"/>

</listOfParameters>

</kineticLaw>

</reaction>

<reaction name="Resistance-Nodulation-Cell Division (RND) TCDB:2.A.60.1.14" id="R_r2495" metaid="_metaR_r2495" reversible="true" sboTerm="SBO:0000185">

<notes>

<body xmlns="<http://www.w3.org/1999/xhtml>">

<p>GENE_ASSOCIATION: </p>

<p>SUBSYSTEM: Transport, extracellular</p>

<p>EC Number: </p>

<p>Confidence Level: 0</p>

<p>AUTHORS: PMID:14579113</p>

<p>NOTES: </p>

</body>

</notes>

<listOfReactants>

<speciesReference species="M_HC02200_e"/>

<speciesReference species="M_hco3_e"/>

</listOfReactants>

<listOfProducts>

<speciesReference species="M_HC02200_c"/>

<speciesReference species="M_hco3_c"/>

</listOfProducts>

<kineticLaw>

<math xmlns="<http://www.w3.org/1998/Math/MathML>">

<ci> FLUX_VALUE </ci>

</math>

<listOfParameters>

<parameter id="LOWER_BOUND" units="mmol_per_gDW_per_hr" value="-1000"/>

<parameter id="UPPER_BOUND" units="mmol_per_gDW_per_hr" value="1000"/>

<parameter id="FLUX_VALUE" units="mmol_per_gDW_per_hr" value="0"/>

<parameter id="OBJECTIVE_COEFFICIENT" units="dimensionless" value="0"/>

</listOfParameters>

</kineticLaw>

</reaction>

<reaction name="Resistance-Nodulation-Cell Division (RND) TCDB:2.A.60.1.14" id="R_r2489" metaid="_metaR_r2489" reversible="true" sboTerm="SBO:0000185">

<notes>

<body xmlns="<http://www.w3.org/1999/xhtml>">

<p>GENE_ASSOCIATION: </p>

<p>SUBSYSTEM: Transport, extracellular</p>

<p>EC Number: </p>

<p>Confidence Level: 0</p>

<p>AUTHORS: PMID:14579113</p>

<p>NOTES: </p>

</body>

</notes>

<listOfReactants>

<speciesReference species="M_hco3_e"/>

<speciesReference species="M_HC02200_e"/>

</listOfReactants>

<listOfProducts>

<speciesReference species="M_HC02200_c"/>

<speciesReference species="M_hco3_c"/>

</listOfProducts>

<kineticLaw>

<math xmlns="<http://www.w3.org/1998/Math/MathML>">

<ci> FLUX_VALUE </ci>

</math>

<listOfParameters>

<parameter id="LOWER_BOUND" units="mmol_per_gDW_per_hr" value="-1000"/>

<parameter id="UPPER_BOUND" units="mmol_per_gDW_per_hr" value="1000"/>

<parameter id="FLUX_VALUE" units="mmol_per_gDW_per_hr" value="0"/>

<parameter id="OBJECTIVE_COEFFICIENT" units="dimensionless" value="0"/>

</listOfParameters>

</kineticLaw>

</reaction>

<reaction name="Resistance-Nodulation-Cell Division (RND) TCDB:2.A.60.1.14" id="R_r2492" metaid="_metaR_r2492" reversible="true" sboTerm="SBO:0000185">

<notes>

<body xmlns="<http://www.w3.org/1999/xhtml>">

<p>GENE_ASSOCIATION: </p>

<p>SUBSYSTEM: Transport, extracellular</p>

<p>EC Number: </p>

<p>Confidence Level: 0</p>

<p>AUTHORS: PMID:14579113</p>

<p>NOTES: </p>

</body>

</notes>

<listOfReactants>

<speciesReference species="M_hco3_e"/>

<speciesReference species="M_HC02200_e"/>

</listOfReactants>

<listOfProducts>

<speciesReference species="M_HC02200_c"/>

<speciesReference species="M_hco3_c"/>

</listOfProducts>

<kineticLaw>

<math xmlns="<http://www.w3.org/1998/Math/MathML>">

<ci> FLUX_VALUE </ci>

</math>

<listOfParameters>

<parameter id="LOWER_BOUND" units="mmol_per_gDW_per_hr" value="-1000"/>

<parameter id="UPPER_BOUND" units="mmol_per_gDW_per_hr" value="1000"/>

<parameter id="FLUX_VALUE" units="mmol_per_gDW_per_hr" value="0"/>

<parameter id="OBJECTIVE_COEFFICIENT" units="dimensionless" value="0"/>

</listOfParameters>

</kineticLaw>

</reaction>

<reaction name="Resistance-Nodulation-Cell Division (RND) TCDB:2.A.60.1.14" id="R_r2484" metaid="_metaR_r2484" reversible="true" sboTerm="SBO:0000185">

<notes>

<body xmlns="<http://www.w3.org/1999/xhtml>">

<p>GENE_ASSOCIATION: </p>

<p>SUBSYSTEM: Transport, extracellular</p>

<p>EC Number: </p>

<p>Confidence Level: 0</p>

<p>AUTHORS: PMID:14579113</p>

<p>NOTES: </p>

</body>

</notes>

<listOfReactants>

<speciesReference species="M_HC02201_e"/>

<speciesReference species="M_hco3_e"/>

</listOfReactants>

<listOfProducts>

<speciesReference species="M_HC02201_c"/>

<speciesReference species="M_hco3_c"/>

</listOfProducts>

<kineticLaw>

<math xmlns="<http://www.w3.org/1998/Math/MathML>">

<ci> FLUX_VALUE </ci>

</math>

<listOfParameters>

<parameter id="LOWER_BOUND" units="mmol_per_gDW_per_hr" value="-1000"/>

<parameter id="UPPER_BOUND" units="mmol_per_gDW_per_hr" value="1000"/>

<parameter id="FLUX_VALUE" units="mmol_per_gDW_per_hr" value="0"/>

<parameter id="OBJECTIVE_COEFFICIENT" units="dimensionless" value="0"/>

</listOfParameters>

</kineticLaw>

</reaction>

<reaction name="Resistance-Nodulation-Cell Division (RND) TCDB:2.A.60.1.14" id="R_r2171" metaid="_metaR_r2171" reversible="true" sboTerm="SBO:0000185">

<notes>

<body xmlns="<http://www.w3.org/1999/xhtml>">

<p>GENE_ASSOCIATION: </p>

<p>SUBSYSTEM: Transport, extracellular</p>

<p>EC Number: </p>

<p>Confidence Level: 0</p>

<p>AUTHORS: PMID:11076394,PMID:11076396,PMID:14579113</p>

<p>NOTES: </p>

</body>

</notes>

<listOfReactants>

<speciesReference species="M_HC02201_c"/>

<speciesReference species="M_hco3_c"/>

</listOfReactants>

<listOfProducts>

<speciesReference species="M_HC02192_c"/>

<speciesReference species="M_hco3_e"/>

</listOfProducts>

<kineticLaw>

<math xmlns="<http://www.w3.org/1998/Math/MathML>">

<ci> FLUX_VALUE </ci>

</math>

<listOfParameters>

<parameter id="LOWER_BOUND" units="mmol_per_gDW_per_hr" value="-1000"/>

<parameter id="UPPER_BOUND" units="mmol_per_gDW_per_hr" value="1000"/>

<parameter id="FLUX_VALUE" units="mmol_per_gDW_per_hr" value="0"/>

<parameter id="OBJECTIVE_COEFFICIENT" units="dimensionless" value="0"/>

</listOfParameters>

</kineticLaw>

</reaction>

<reaction name="Resistance-Nodulation-Cell Division (RND) TCDB:2.A.60.1.14" id="R_r2174" metaid="_metaR_r2174" reversible="true" sboTerm="SBO:0000185">

<notes>

<body xmlns="<http://www.w3.org/1999/xhtml>">

<p>GENE_ASSOCIATION: </p>

<p>SUBSYSTEM: Transport, extracellular</p>

<p>EC Number: </p>

<p>Confidence Level: 0</p>

<p>AUTHORS: PMID:11076394,PMID:11076396,PMID:14579113</p>

<p>NOTES: </p>

</body>

</notes>

<listOfReactants>

<speciesReference species="M_HC02201_c"/>

<speciesReference species="M_hco3_c"/>

</listOfReactants>

<listOfProducts>

<speciesReference species="M_HC02193_c"/>

<speciesReference species="M_hco3_e"/>

</listOfProducts>

<kineticLaw>

<math xmlns="<http://www.w3.org/1998/Math/MathML>">

<ci> FLUX_VALUE </ci>

</math>

<listOfParameters>

<parameter id="LOWER_BOUND" units="mmol_per_gDW_per_hr" value="-1000"/>

<parameter id="UPPER_BOUND" units="mmol_per_gDW_per_hr" value="1000"/>

<parameter id="FLUX_VALUE" units="mmol_per_gDW_per_hr" value="0"/>

<parameter id="OBJECTIVE_COEFFICIENT" units="dimensionless" value="0"/>

</listOfParameters>

</kineticLaw>

</reaction>

<reaction name="Resistance-Nodulation-Cell Division (RND) TCDB:2.A.60.1.14" id="R_r2183" metaid="_metaR_r2183" reversible="true" sboTerm="SBO:0000185">

<notes>

<body xmlns="<http://www.w3.org/1999/xhtml>">

<p>GENE_ASSOCIATION: 6566.1</p>

<p>SUBSYSTEM: Transport, extracellular</p>

<p>EC Number: </p>

<p>Confidence Level: 0</p>

<p>AUTHORS: PMID:11076394,PMID:11076396,PMID:14579113</p>

<p>NOTES: </p>

</body>

</notes>

<listOfReactants>

<speciesReference species="M_HC02201_c"/>

<speciesReference species="M_hco3_c"/>

</listOfReactants>

<listOfProducts>

<speciesReference species="M_HC02196_c"/>

<speciesReference species="M_hco3_e"/>

</listOfProducts>

<kineticLaw>

<math xmlns="<http://www.w3.org/1998/Math/MathML>">

<ci> FLUX_VALUE </ci>

</math>

<listOfParameters>

<parameter id="LOWER_BOUND" units="mmol_per_gDW_per_hr" value="-1000"/>

<parameter id="UPPER_BOUND" units="mmol_per_gDW_per_hr" value="1000"/>

<parameter id="FLUX_VALUE" units="mmol_per_gDW_per_hr" value="0"/>

<parameter id="OBJECTIVE_COEFFICIENT" units="dimensionless" value="0"/>

</listOfParameters>

</kineticLaw>

</reaction>

<reaction name="Resistance-Nodulation-Cell Division (RND) TCDB:2.A.60.1.14" id="R_r2180" metaid="_metaR_r2180" reversible="true" sboTerm="SBO:0000185">

<notes>

<body xmlns="<http://www.w3.org/1999/xhtml>">

<p>GENE_ASSOCIATION: 6566.1</p>

<p>SUBSYSTEM: Transport, extracellular</p>

<p>EC Number: </p>

<p>Confidence Level: 0</p>

<p>AUTHORS: PMID:11076394,PMID:11076396,PMID:14579113</p>

<p>NOTES: </p>

</body>

</notes>

<listOfReactants>

<speciesReference species="M_HC02201_c"/>

<speciesReference species="M_hco3_c"/>

</listOfReactants>

<listOfProducts>

<speciesReference species="M_HC02195_c"/>

<speciesReference species="M_hco3_e"/>

</listOfProducts>

<kineticLaw>

<math xmlns="<http://www.w3.org/1998/Math/MathML>">

<ci> FLUX_VALUE </ci>

</math>

<listOfParameters>

<parameter id="LOWER_BOUND" units="mmol_per_gDW_per_hr" value="-1000"/>

<parameter id="UPPER_BOUND" units="mmol_per_gDW_per_hr" value="1000"/>

<parameter id="FLUX_VALUE" units="mmol_per_gDW_per_hr" value="0"/>

<parameter id="OBJECTIVE_COEFFICIENT" units="dimensionless" value="0"/>

</listOfParameters>

</kineticLaw>

</reaction>

<reaction name="Resistance-Nodulation-Cell Division (RND) TCDB:2.A.60.1.14" id="R_r2177" metaid="_metaR_r2177" reversible="true" sboTerm="SBO:0000185">

<notes>

<body xmlns="<http://www.w3.org/1999/xhtml>">

<p>GENE_ASSOCIATION: </p>

<p>SUBSYSTEM: Transport, extracellular</p>

<p>EC Number: </p>

<p>Confidence Level: 0</p>

<p>AUTHORS: PMID:11076394,PMID:11076396,PMID:14579113</p>

<p>NOTES: </p>

</body>

</notes>

<listOfReactants>

<speciesReference species="M_HC02201_c"/>

<speciesReference species="M_hco3_c"/>

</listOfReactants>

<listOfProducts>

<speciesReference species="M_HC02194_c"/>

<speciesReference species="M_hco3_e"/>

</listOfProducts>

<kineticLaw>

<math xmlns="<http://www.w3.org/1998/Math/MathML>">

<ci> FLUX_VALUE </ci>

</math>

<listOfParameters>

<parameter id="LOWER_BOUND" units="mmol_per_gDW_per_hr" value="-1000"/>

<parameter id="UPPER_BOUND" units="mmol_per_gDW_per_hr" value="1000"/>

<parameter id="FLUX_VALUE" units="mmol_per_gDW_per_hr" value="0"/>

<parameter id="OBJECTIVE_COEFFICIENT" units="dimensionless" value="0"/>

</listOfParameters>

</kineticLaw>

</reaction>

<reaction name="Resistance-Nodulation-Cell Division (RND) TCDB:2.A.60.1.14" id="R_r2192" metaid="_metaR_r2192" reversible="true" sboTerm="SBO:0000185">

<notes>

<body xmlns="<http://www.w3.org/1999/xhtml>">

<p>GENE_ASSOCIATION: 6566.1</p>

<p>SUBSYSTEM: Transport, extracellular</p>

<p>EC Number: </p>

<p>Confidence Level: 0</p>

<p>AUTHORS: PMID:11076394,PMID:11076396,PMID:14579113</p>

<p>NOTES: </p>

</body>

</notes>

<listOfReactants>

<speciesReference species="M_HC02201_c"/>

<speciesReference species="M_hco3_c"/>

</listOfReactants>

<listOfProducts>

<speciesReference species="M_HC02220_c"/>

<speciesReference species="M_hco3_e"/>

</listOfProducts>

<kineticLaw>

<math xmlns="<http://www.w3.org/1998/Math/MathML>">

<ci> FLUX_VALUE </ci>

</math>

<listOfParameters>

<parameter id="LOWER_BOUND" units="mmol_per_gDW_per_hr" value="-1000"/>

<parameter id="UPPER_BOUND" units="mmol_per_gDW_per_hr" value="1000"/>

<parameter id="FLUX_VALUE" units="mmol_per_gDW_per_hr" value="0"/>

<parameter id="OBJECTIVE_COEFFICIENT" units="dimensionless" value="0"/>

</listOfParameters>

</kineticLaw>

</reaction>

<reaction name="Resistance-Nodulation-Cell Division (RND) TCDB:2.A.60.1.14" id="R_r2186" metaid="_metaR_r2186" reversible="true" sboTerm="SBO:0000185">

<notes>

<body xmlns="<http://www.w3.org/1999/xhtml>">

<p>GENE_ASSOCIATION: 6566.1</p>

<p>SUBSYSTEM: Transport, extracellular</p>

<p>EC Number: </p>

<p>Confidence Level: 0</p>

<p>AUTHORS: PMID:11076394,PMID:11076396,PMID:14579113</p>

<p>NOTES: </p>

</body>

</notes>

<listOfReactants>

<speciesReference species="M_HC02201_c"/>

<speciesReference species="M_hco3_c"/>

</listOfReactants>

<listOfProducts>

<speciesReference species="M_HC02197_c"/>

<speciesReference species="M_hco3_e"/>

</listOfProducts>

<kineticLaw>

<math xmlns="<http://www.w3.org/1998/Math/MathML>">

<ci> FLUX_VALUE </ci>

</math>

<listOfParameters>

<parameter id="LOWER_BOUND" units="mmol_per_gDW_per_hr" value="-1000"/>

<parameter id="UPPER_BOUND" units="mmol_per_gDW_per_hr" value="1000"/>

<parameter id="FLUX_VALUE" units="mmol_per_gDW_per_hr" value="0"/>

<parameter id="OBJECTIVE_COEFFICIENT" units="dimensionless" value="0"/>

</listOfParameters>

</kineticLaw>

</reaction>

<reaction name="Resistance-Nodulation-Cell Division (RND) TCDB:2.A.60.1.14" id="R_r2214" metaid="_metaR_r2214" reversible="true" sboTerm="SBO:0000185">

<notes>

<body xmlns="<http://www.w3.org/1999/xhtml>">

<p>GENE_ASSOCIATION: 6566.1</p>

<p>SUBSYSTEM: Transport, extracellular</p>

<p>EC Number: </p>

<p>Confidence Level: 0</p>

<p>AUTHORS: PMID:11076396,PMID:14579113</p>

<p>NOTES: </p>

</body>

</notes>

<listOfReactants>

<speciesReference species="M_HC02201_c"/>

<speciesReference species="M_hco3_c"/>

</listOfReactants>

<listOfProducts>

<speciesReference species="M_HC02187_c"/>

<speciesReference species="M_hco3_e"/>

</listOfProducts>

<kineticLaw>

<math xmlns="<http://www.w3.org/1998/Math/MathML>">

<ci> FLUX_VALUE </ci>

</math>

<listOfParameters>

<parameter id="LOWER_BOUND" units="mmol_per_gDW_per_hr" value="-1000"/>

<parameter id="UPPER_BOUND" units="mmol_per_gDW_per_hr" value="1000"/>

<parameter id="FLUX_VALUE" units="mmol_per_gDW_per_hr" value="0"/>

<parameter id="OBJECTIVE_COEFFICIENT" units="dimensionless" value="0"/>

</listOfParameters>

</kineticLaw>

</reaction>

<reaction name="Resistance-Nodulation-Cell Division (RND) TCDB:2.A.60.1.5" id="R_r2241" metaid="_metaR_r2241" reversible="true" sboTerm="SBO:0000185">

<notes>

<body xmlns="<http://www.w3.org/1999/xhtml>">

<p>GENE_ASSOCIATION: </p>

<p>SUBSYSTEM: Transport, extracellular</p>

<p>EC Number: </p>

<p>Confidence Level: 0</p>

<p>AUTHORS: PMID:11076396,PMID:14579113</p>

<p>NOTES: </p>

</body>

</notes>

<listOfReactants>

<speciesReference species="M_HC02201_c"/>

<speciesReference species="M_hco3_c"/>

</listOfReactants>

<listOfProducts>

<speciesReference species="M_HC02180_c"/>

<speciesReference species="M_hco3_e"/>

</listOfProducts>

<kineticLaw>

<math xmlns="<http://www.w3.org/1998/Math/MathML>">

<ci> FLUX_VALUE </ci>

</math>

<listOfParameters>

<parameter id="LOWER_BOUND" units="mmol_per_gDW_per_hr" value="-1000"/>

<parameter id="UPPER_BOUND" units="mmol_per_gDW_per_hr" value="1000"/>

<parameter id="FLUX_VALUE" units="mmol_per_gDW_per_hr" value="0"/>

<parameter id="OBJECTIVE_COEFFICIENT" units="dimensionless" value="0"/>

</listOfParameters>

</kineticLaw>

</reaction>

<reaction name="Resistance-Nodulation-Cell Division (RND) TCDB:2.A.60.1.5" id="R_r2238" metaid="_metaR_r2238" reversible="true" sboTerm="SBO:0000185">

<notes>

<body xmlns="<http://www.w3.org/1999/xhtml>">

<p>GENE_ASSOCIATION: </p>

<p>SUBSYSTEM: Transport, extracellular</p>

<p>EC Number: </p>

<p>Confidence Level: 0</p>

<p>AUTHORS: PMID:11076396,PMID:14579113</p>

<p>NOTES: </p>

</body>

</notes>

<listOfReactants>

<speciesReference species="M_HC02201_c"/>

<speciesReference species="M_hco3_c"/>

</listOfReactants>

<listOfProducts>

<speciesReference species="M_HC02179_c"/>

<speciesReference species="M_hco3_e"/>

</listOfProducts>

<kineticLaw>

<math xmlns="<http://www.w3.org/1998/Math/MathML>">

<ci> FLUX_VALUE </ci>

</math>

<listOfParameters>

<parameter id="LOWER_BOUND" units="mmol_per_gDW_per_hr" value="-1000"/>

<parameter id="UPPER_BOUND" units="mmol_per_gDW_per_hr" value="1000"/>

<parameter id="FLUX_VALUE" units="mmol_per_gDW_per_hr" value="0"/>

<parameter id="OBJECTIVE_COEFFICIENT" units="dimensionless" value="0"/>

</listOfParameters>

</kineticLaw>

</reaction>

<reaction name="Resistance-Nodulation-Cell Division (RND) TCDB:2.A.60.1.5" id="R_r2253" metaid="_metaR_r2253" reversible="true" sboTerm="SBO:0000185">

<notes>

<body xmlns="<http://www.w3.org/1999/xhtml>">

<p>GENE_ASSOCIATION: </p>

<p>SUBSYSTEM: Transport, extracellular</p>

<p>EC Number: </p>

<p>Confidence Level: 0</p>

<p>AUTHORS: PMID:11076396,PMID:14579113</p>

<p>NOTES: </p>

</body>

</notes>

<listOfReactants>

<speciesReference species="M_HC02201_c"/>

<speciesReference species="M_hco3_c"/>

</listOfReactants>

<listOfProducts>

<speciesReference species="M_leuktrF4_c"/>

<speciesReference species="M_hco3_e"/>

</listOfProducts>

<kineticLaw>

<math xmlns="<http://www.w3.org/1998/Math/MathML>">

<ci> FLUX_VALUE </ci>

</math>

<listOfParameters>

<parameter id="LOWER_BOUND" units="mmol_per_gDW_per_hr" value="-1000"/>

<parameter id="UPPER_BOUND" units="mmol_per_gDW_per_hr" value="1000"/>

<parameter id="FLUX_VALUE" units="mmol_per_gDW_per_hr" value="0"/>

<parameter id="OBJECTIVE_COEFFICIENT" units="dimensionless" value="0"/>

</listOfParameters>

</kineticLaw>

</reaction>

<reaction name="Resistance-Nodulation-Cell Division (RND) TCDB:2.A.60.1.5" id="R_r2268" metaid="_metaR_r2268" reversible="true" sboTerm="SBO:0000185">

<notes>

<body xmlns="<http://www.w3.org/1999/xhtml>">

<p>GENE_ASSOCIATION: </p>

<p>SUBSYSTEM: Transport, extracellular</p>

<p>EC Number: </p>

<p>Confidence Level: 0</p>

<p>AUTHORS: PMID:11076396,PMID:14579113</p>

<p>NOTES: </p>

</body>

</notes>

<listOfReactants>

<speciesReference species="M_HC02201_c"/>

<speciesReference species="M_hco3_c"/>

</listOfReactants>

<listOfProducts>

<speciesReference species="M_HC02205_c"/>

<speciesReference species="M_hco3_e"/>

</listOfProducts>

<kineticLaw>

<math xmlns="<http://www.w3.org/1998/Math/MathML>">

<ci> FLUX_VALUE </ci>

</math>

<listOfParameters>

<parameter id="LOWER_BOUND" units="mmol_per_gDW_per_hr" value="-1000"/>

<parameter id="UPPER_BOUND" units="mmol_per_gDW_per_hr" value="1000"/>

<parameter id="FLUX_VALUE" units="mmol_per_gDW_per_hr" value="0"/>

<parameter id="OBJECTIVE_COEFFICIENT" units="dimensionless" value="0"/>

</listOfParameters>

</kineticLaw>

</reaction>

<reaction name="Resistance-Nodulation-Cell Division (RND) TCDB:2.A.60.1.5" id="R_r2265" metaid="_metaR_r2265" reversible="true" sboTerm="SBO:0000185">

<notes>

<body xmlns="<http://www.w3.org/1999/xhtml>">

<p>GENE_ASSOCIATION: </p>

<p>SUBSYSTEM: Transport, extracellular</p>

<p>EC Number: </p>

<p>Confidence Level: 0</p>

<p>AUTHORS: PMID:11076396,PMID:14579113</p>

<p>NOTES: </p>

</body>

</notes>

<listOfReactants>

<speciesReference species="M_HC02201_c"/>

<speciesReference species="M_hco3_c"/>

</listOfReactants>

<listOfProducts>

<speciesReference species="M_HC02204_c"/>

<speciesReference species="M_hco3_e"/>

</listOfProducts>

<kineticLaw>

<math xmlns="<http://www.w3.org/1998/Math/MathML>">

<ci> FLUX_VALUE </ci>

</math>

<listOfParameters>

<parameter id="LOWER_BOUND" units="mmol_per_gDW_per_hr" value="-1000"/>

<parameter id="UPPER_BOUND" units="mmol_per_gDW_per_hr" value="1000"/>

<parameter id="FLUX_VALUE" units="mmol_per_gDW_per_hr" value="0"/>

<parameter id="OBJECTIVE_COEFFICIENT" units="dimensionless" value="0"/>

</listOfParameters>

</kineticLaw>

</reaction>

<reaction name="Resistance-Nodulation-Cell Division (RND) TCDB:2.A.60.1.5" id="R_r2292" metaid="_metaR_r2292" reversible="true" sboTerm="SBO:0000185">

<notes>

<body xmlns="<http://www.w3.org/1999/xhtml>">

<p>GENE_ASSOCIATION: </p>

<p>SUBSYSTEM: Transport, extracellular</p>

<p>EC Number: </p>

<p>Confidence Level: 0</p>

<p>AUTHORS: PMID:11076396,PMID:14579113</p>

<p>NOTES: </p>

</body>

</notes>

<listOfReactants>

<speciesReference species="M_HC02201_c"/>

<speciesReference species="M_hco3_c"/>

</listOfReactants>

<listOfProducts>

<speciesReference species="M_HC02214_c"/>

<speciesReference species="M_hco3_e"/>

</listOfProducts>

<kineticLaw>

<math xmlns="<http://www.w3.org/1998/Math/MathML>">

<ci> FLUX_VALUE </ci>

</math>

<listOfParameters>

<parameter id="LOWER_BOUND" units="mmol_per_gDW_per_hr" value="-1000"/>

<parameter id="UPPER_BOUND" units="mmol_per_gDW_per_hr" value="1000"/>

<parameter id="FLUX_VALUE" units="mmol_per_gDW_per_hr" value="0"/>

<parameter id="OBJECTIVE_COEFFICIENT" units="dimensionless" value="0"/>

</listOfParameters>

</kineticLaw>

</reaction>

<reaction name="Resistance-Nodulation-Cell Division (RND) TCDB:2.A.60.1.5" id="R_r2289" metaid="_metaR_r2289" reversible="true" sboTerm="SBO:0000185">

<notes>

<body xmlns="<http://www.w3.org/1999/xhtml>">

<p>GENE_ASSOCIATION: </p>

<p>SUBSYSTEM: Transport, extracellular</p>

<p>EC Number: </p>

<p>Confidence Level: 0</p>

<p>AUTHORS: PMID:11076396,PMID:14579113</p>

<p>NOTES: </p>

</body>

</notes>

<listOfReactants>

<speciesReference species="M_HC02201_c"/>

<speciesReference species="M_hco3_c"/>

</listOfReactants>

<listOfProducts>

<speciesReference species="M_HC02213_c"/>

<speciesReference species="M_hco3_e"/>

</listOfProducts>

<kineticLaw>

<math xmlns="<http://www.w3.org/1998/Math/MathML>">

<ci> FLUX_VALUE </ci>

</math>

<listOfParameters>

<parameter id="LOWER_BOUND" units="mmol_per_gDW_per_hr" value="-1000"/>

<parameter id="UPPER_BOUND" units="mmol_per_gDW_per_hr" value="1000"/>

<parameter id="FLUX_VALUE" units="mmol_per_gDW_per_hr" value="0"/>

<parameter id="OBJECTIVE_COEFFICIENT" units="dimensionless" value="0"/>

</listOfParameters>

</kineticLaw>

</reaction>

<reaction name="Resistance-Nodulation-Cell Division (RND) TCDB:2.A.60.1.5" id="R_r2277" metaid="_metaR_r2277" reversible="true" sboTerm="SBO:0000185">

<notes>

<body xmlns="<http://www.w3.org/1999/xhtml>">

<p>GENE_ASSOCIATION: </p>

<p>SUBSYSTEM: Transport, extracellular</p>

<p>EC Number: </p>

<p>Confidence Level: 0</p>

<p>AUTHORS: PMID:11076396,PMID:14579113</p>

<p>NOTES: </p>

</body>

</notes>

<listOfReactants>

<speciesReference species="M_HC02201_c"/>

<speciesReference species="M_hco3_c"/>

</listOfReactants>

<listOfProducts>

<speciesReference species="M_HC02208_c"/>

<speciesReference species="M_hco3_e"/>

</listOfProducts>

<kineticLaw>

<math xmlns="<http://www.w3.org/1998/Math/MathML>">

<ci> FLUX_VALUE </ci>

</math>

<listOfParameters>

<parameter id="LOWER_BOUND" units="mmol_per_gDW_per_hr" value="-1000"/>

<parameter id="UPPER_BOUND" units="mmol_per_gDW_per_hr" value="1000"/>

<parameter id="FLUX_VALUE" units="mmol_per_gDW_per_hr" value="0"/>

<parameter id="OBJECTIVE_COEFFICIENT" units="dimensionless" value="0"/>

</listOfParameters>

</kineticLaw>

</reaction>

<reaction name="Resistance-Nodulation-Cell Division (RND) TCDB:2.A.60.1.5" id="R_r2283" metaid="_metaR_r2283" reversible="true" sboTerm="SBO:0000185">

<notes>

<body xmlns="<http://www.w3.org/1999/xhtml>">

<p>GENE_ASSOCIATION: </p>

<p>SUBSYSTEM: Transport, extracellular</p>

<p>EC Number: </p>

<p>Confidence Level: 0</p>

<p>AUTHORS: PMID:11076396,PMID:14579113</p>

<p>NOTES: </p>

</body>

</notes>

<listOfReactants>

<speciesReference species="M_HC02201_c"/>

<speciesReference species="M_hco3_c"/>

</listOfReactants>

<listOfProducts>

<speciesReference species="M_HC02210_c"/>

<speciesReference species="M_hco3_e"/>

</listOfProducts>

<kineticLaw>

<math xmlns="<http://www.w3.org/1998/Math/MathML>">

<ci> FLUX_VALUE </ci>

</math>

<listOfParameters>

<parameter id="LOWER_BOUND" units="mmol_per_gDW_per_hr" value="-1000"/>

<parameter id="UPPER_BOUND" units="mmol_per_gDW_per_hr" value="1000"/>

<parameter id="FLUX_VALUE" units="mmol_per_gDW_per_hr" value="0"/>

<parameter id="OBJECTIVE_COEFFICIENT" units="dimensionless" value="0"/>

</listOfParameters>

</kineticLaw>

</reaction>

<reaction name="Resistance-Nodulation-Cell Division (RND) TCDB:2.A.60.1.5" id="R_r2298" metaid="_metaR_r2298" reversible="true" sboTerm="SBO:0000185">

<notes>

<body xmlns="<http://www.w3.org/1999/xhtml>">

<p>GENE_ASSOCIATION: </p>

<p>SUBSYSTEM: Transport, extracellular</p>

<p>EC Number: </p>

<p>Confidence Level: 0</p>

<p>AUTHORS: PMID:11076396,PMID:14579113</p>

<p>NOTES: </p>

</body>

</notes>

<listOfReactants>

<speciesReference species="M_HC02201_c"/>

<speciesReference species="M_hco3_c"/>

</listOfReactants>

<listOfProducts>

<speciesReference species="M_HC02216_c"/>

<speciesReference species="M_hco3_e"/>

</listOfProducts>

<kineticLaw>

<math xmlns="<http://www.w3.org/1998/Math/MathML>">

<ci> FLUX_VALUE </ci>

</math>

<listOfParameters>

<parameter id="LOWER_BOUND" units="mmol_per_gDW_per_hr" value="-1000"/>

<parameter id="UPPER_BOUND" units="mmol_per_gDW_per_hr" value="1000"/>

<parameter id="FLUX_VALUE" units="mmol_per_gDW_per_hr" value="0"/>

<parameter id="OBJECTIVE_COEFFICIENT" units="dimensionless" value="0"/>

</listOfParameters>

</kineticLaw>

</reaction>

<reaction name="Resistance-Nodulation-Cell Division (RND) TCDB:2.A.60.1.5" id="R_r2301" metaid="_metaR_r2301" reversible="true" sboTerm="SBO:0000185">

<notes>

<body xmlns="<http://www.w3.org/1999/xhtml>">

<p>GENE_ASSOCIATION: </p>

<p>SUBSYSTEM: Transport, extracellular</p>

<p>EC Number: </p>

<p>Confidence Level: 0</p>

<p>AUTHORS: PMID:11076396,PMID:14579113</p>

<p>NOTES: </p>

</body>

</notes>

<listOfReactants>

<speciesReference species="M_HC02201_c"/>

<speciesReference species="M_hco3_c"/>

</listOfReactants>

<listOfProducts>

<speciesReference species="M_HC02217_c"/>

<speciesReference species="M_hco3_e"/>

</listOfProducts>

<kineticLaw>

<math xmlns="<http://www.w3.org/1998/Math/MathML>">

<ci> FLUX_VALUE </ci>

</math>

<listOfParameters>

<parameter id="LOWER_BOUND" units="mmol_per_gDW_per_hr" value="-1000"/>

<parameter id="UPPER_BOUND" units="mmol_per_gDW_per_hr" value="1000"/>

<parameter id="FLUX_VALUE" units="mmol_per_gDW_per_hr" value="0"/>

<parameter id="OBJECTIVE_COEFFICIENT" units="dimensionless" value="0"/>

</listOfParameters>

</kineticLaw>

</reaction>

<reaction name="Resistance-Nodulation-Cell Division (RND) TCDB:2.A.60.1.14" id="R_r2487" metaid="_metaR_r2487" reversible="true" sboTerm="SBO:0000185">

<notes>

<body xmlns="<http://www.w3.org/1999/xhtml>">

<p>GENE_ASSOCIATION: </p>

<p>SUBSYSTEM: Transport, extracellular</p>

<p>EC Number: </p>

<p>Confidence Level: 0</p>

<p>AUTHORS: PMID:14579113</p>

<p>NOTES: </p>

</body>

</notes>

<listOfReactants>

<speciesReference species="M_HC02201_e"/>

<speciesReference species="M_hco3_e"/>

</listOfReactants>

<listOfProducts>

<speciesReference species="M_HC02201_c"/>

<speciesReference species="M_hco3_c"/>

</listOfProducts>

<kineticLaw>

<math xmlns="<http://www.w3.org/1998/Math/MathML>">

<ci> FLUX_VALUE </ci>

</math>

<listOfParameters>

<parameter id="LOWER_BOUND" units="mmol_per_gDW_per_hr" value="-1000"/>

<parameter id="UPPER_BOUND" units="mmol_per_gDW_per_hr" value="1000"/>

<parameter id="FLUX_VALUE" units="mmol_per_gDW_per_hr" value="0"/>

<parameter id="OBJECTIVE_COEFFICIENT" units="dimensionless" value="0"/>

</listOfParameters>

</kineticLaw>

</reaction>

<reaction name="Resistance-Nodulation-Cell Division (RND) TCDB:2.A.60.1.14" id="R_r2496" metaid="_metaR_r2496" reversible="true" sboTerm="SBO:0000185">

<notes>

<body xmlns="<http://www.w3.org/1999/xhtml>">

<p>GENE_ASSOCIATION: </p>

<p>SUBSYSTEM: Transport, extracellular</p>

<p>EC Number: </p>

<p>Confidence Level: 0</p>

<p>AUTHORS: PMID:14579113</p>

<p>NOTES: </p>

</body>

</notes>

<listOfReactants>

<speciesReference species="M_hco3_e"/>

<speciesReference species="M_HC02201_e"/>

</listOfReactants>

<listOfProducts>

<speciesReference species="M_HC02201_c"/>

<speciesReference species="M_hco3_c"/>

</listOfProducts>

<kineticLaw>

<math xmlns="<http://www.w3.org/1998/Math/MathML>">

<ci> FLUX_VALUE </ci>

</math>

<listOfParameters>

<parameter id="LOWER_BOUND" units="mmol_per_gDW_per_hr" value="-1000"/>

<parameter id="UPPER_BOUND" units="mmol_per_gDW_per_hr" value="1000"/>

<parameter id="FLUX_VALUE" units="mmol_per_gDW_per_hr" value="0"/>

<parameter id="OBJECTIVE_COEFFICIENT" units="dimensionless" value="0"/>

</listOfParameters>

</kineticLaw>

</reaction>

<reaction name="Resistance-Nodulation-Cell Division (RND) TCDB:2.A.60.1.14" id="R_r2490" metaid="_metaR_r2490" reversible="true" sboTerm="SBO:0000185">

<notes>

<body xmlns="<http://www.w3.org/1999/xhtml>">

<p>GENE_ASSOCIATION: </p>

<p>SUBSYSTEM: Transport, extracellular</p>

<p>EC Number: </p>

<p>Confidence Level: 0</p>

<p>AUTHORS: PMID:14579113</p>

<p>NOTES: </p>

</body>

</notes>

<listOfReactants>

<speciesReference species="M_hco3_e"/>

<speciesReference species="M_HC02201_e"/>

</listOfReactants>

<listOfProducts>

<speciesReference species="M_HC02201_c"/>

<speciesReference species="M_hco3_c"/>

</listOfProducts>

<kineticLaw>

<math xmlns="<http://www.w3.org/1998/Math/MathML>">

<ci> FLUX_VALUE </ci>

</math>

<listOfParameters>

<parameter id="LOWER_BOUND" units="mmol_per_gDW_per_hr" value="-1000"/>

<parameter id="UPPER_BOUND" units="mmol_per_gDW_per_hr" value="1000"/>

<parameter id="FLUX_VALUE" units="mmol_per_gDW_per_hr" value="0"/>

<parameter id="OBJECTIVE_COEFFICIENT" units="dimensionless" value="0"/>

</listOfParameters>

</kineticLaw>

</reaction>

<reaction name="Resistance-Nodulation-Cell Division (RND) TCDB:2.A.60.1.14" id="R_r2493" metaid="_metaR_r2493" reversible="true" sboTerm="SBO:0000185">

<notes>

<body xmlns="<http://www.w3.org/1999/xhtml>">

<p>GENE_ASSOCIATION: </p>

<p>SUBSYSTEM: Transport, extracellular</p>

<p>EC Number: </p>

<p>Confidence Level: 0</p>

<p>AUTHORS: PMID:14579113</p>

<p>NOTES: </p>

</body>

</notes>

<listOfReactants>

<speciesReference species="M_hco3_e"/>

<speciesReference species="M_HC02201_e"/>

</listOfReactants>

<listOfProducts>

<speciesReference species="M_HC02201_c"/>

<speciesReference species="M_hco3_c"/>

</listOfProducts>

<kineticLaw>

<math xmlns="<http://www.w3.org/1998/Math/MathML>">

<ci> FLUX_VALUE </ci>

</math>

<listOfParameters>

<parameter id="LOWER_BOUND" units="mmol_per_gDW_per_hr" value="-1000"/>

<parameter id="UPPER_BOUND" units="mmol_per_gDW_per_hr" value="1000"/>

<parameter id="FLUX_VALUE" units="mmol_per_gDW_per_hr" value="0"/>

<parameter id="OBJECTIVE_COEFFICIENT" units="dimensionless" value="0"/>

</listOfParameters>

</kineticLaw>

</reaction>

<reaction name="RE2958" id="R_RE2958C" metaid="_metaR_RE2958C" reversible="true" sboTerm="SBO:0000176">

<notes>

<body xmlns="<http://www.w3.org/1999/xhtml>">

<p>GENE_ASSOCIATION: </p>

<p>SUBSYSTEM: Eicosanoid metabolism</p>

<p>EC Number: </p>

<p>Confidence Level: 0</p>

<p>AUTHORS: PMID:10534257</p>

<p>NOTES: </p>

</body>

</notes>

<listOfReactants>

<speciesReference species="M_prostgi2_r"/>

<speciesReference species="M_h2o_r"/>

</listOfReactants>

<listOfProducts>

<speciesReference species="M_CE0955_r"/>

</listOfProducts>

<kineticLaw>

<math xmlns="<http://www.w3.org/1998/Math/MathML>">

<ci> FLUX_VALUE </ci>

</math>

<listOfParameters>

<parameter id="LOWER_BOUND" units="mmol_per_gDW_per_hr" value="-1000"/>

<parameter id="UPPER_BOUND" units="mmol_per_gDW_per_hr" value="1000"/>

<parameter id="FLUX_VALUE" units="mmol_per_gDW_per_hr" value="0"/>

<parameter id="OBJECTIVE_COEFFICIENT" units="dimensionless" value="0"/>

</listOfParameters>

</kineticLaw>

</reaction>

<reaction name="Prostaglandin I2 synthase" id="R_PGISr" metaid="_metaR_PGISr" reversible="true" sboTerm="SBO:0000176">

<notes>

<body xmlns="<http://www.w3.org/1999/xhtml>">

<p>GENE_ASSOCIATION: 5740.1</p>

<p>SUBSYSTEM: Eicosanoid metabolism</p>

<p>EC Number: 5.3.99.4</p>

<p>Confidence Level: 4</p>

<p>AUTHORS: PMID:2120373,PMID:2491846</p>

<p>NOTES: NJ</p>

</body>

</notes>

<listOfReactants>

<speciesReference species="M_prostgh2_r"/>

</listOfReactants>

<listOfProducts>

<speciesReference species="M_prostgi2_r"/>

</listOfProducts>

<kineticLaw>

<math xmlns="<http://www.w3.org/1998/Math/MathML>">

<ci> FLUX_VALUE </ci>

</math>

<listOfParameters>

<parameter id="LOWER_BOUND" units="mmol_per_gDW_per_hr" value="-1000"/>

<parameter id="UPPER_BOUND" units="mmol_per_gDW_per_hr" value="1000"/>

<parameter id="FLUX_VALUE" units="mmol_per_gDW_per_hr" value="0"/>

<parameter id="OBJECTIVE_COEFFICIENT" units="dimensionless" value="0"/>

</listOfParameters>

</kineticLaw>

</reaction>

<reaction name="RE3477" id="R_RE3477C" metaid="_metaR_RE3477C" reversible="true" sboTerm="SBO:0000185">

<notes>

<body xmlns="<http://www.w3.org/1999/xhtml>">

<p>GENE_ASSOCIATION: </p>

<p>SUBSYSTEM: Eicosanoid metabolism</p>

<p>EC Number: </p>

<p>Confidence Level: 0</p>

<p>AUTHORS: PMID:3461463,PMID:3755252</p>

<p>NOTES: </p>

</body>

</notes>

<listOfReactants>

<speciesReference species="M_txa2_r"/>

<speciesReference species="M_h2o_r"/>

</listOfReactants>

<listOfProducts>

<speciesReference species="M_h2_c"/>

<speciesReference species="M_CE1447_c"/>

</listOfProducts>

<kineticLaw>

<math xmlns="<http://www.w3.org/1998/Math/MathML>">

<ci> FLUX_VALUE </ci>

</math>

<listOfParameters>

<parameter id="LOWER_BOUND" units="mmol_per_gDW_per_hr" value="-1000"/>

<parameter id="UPPER_BOUND" units="mmol_per_gDW_per_hr" value="1000"/>

<parameter id="FLUX_VALUE" units="mmol_per_gDW_per_hr" value="0"/>

<parameter id="OBJECTIVE_COEFFICIENT" units="dimensionless" value="0"/>

</listOfParameters>

</kineticLaw>

</reaction>

<reaction name="Thromboxane-A synthase" id="R_TXASr" metaid="_metaR_TXASr" reversible="false" sboTerm="SBO:0000176">

<notes>

<body xmlns="<http://www.w3.org/1999/xhtml>">

<p>GENE_ASSOCIATION: 6916.1</p>

<p>SUBSYSTEM: Eicosanoid metabolism</p>

<p>EC Number: 5.3.99.5</p>

<p>Confidence Level: 0</p>

<p>AUTHORS: PMID:10903770,PMID:2491846,PMID:7811713</p>

<p>NOTES: NJ</p>

</body>

</notes>

<listOfReactants>

<speciesReference species="M_prostgh2_r"/>

</listOfReactants>

<listOfProducts>

<speciesReference species="M_txa2_r"/>

</listOfProducts>

<kineticLaw>

<math xmlns="<http://www.w3.org/1998/Math/MathML>">

<ci> FLUX_VALUE </ci>

</math>

<listOfParameters>

<parameter id="LOWER_BOUND" units="mmol_per_gDW_per_hr" value="0"/>

<parameter id="UPPER_BOUND" units="mmol_per_gDW_per_hr" value="1000"/>

<parameter id="FLUX_VALUE" units="mmol_per_gDW_per_hr" value="0"/>

<parameter id="OBJECTIVE_COEFFICIENT" units="dimensionless" value="0"/>

</listOfParameters>

</kineticLaw>

</reaction>

<reaction name="Palmitoyl-CoA hydrolase Fatty acid elongation in mitochondria EC:3.1.2.22 EC:3.1.2.2" id="R_r0308" metaid="_metaR_r0308" reversible="false" sboTerm="SBO:0000176">

<notes>

<body xmlns="<http://www.w3.org/1999/xhtml>">

<p>GENE_ASSOCIATION: 6576.1</p>

<p>SUBSYSTEM: Fatty acid oxidation</p>

<p>EC Number: 3.1.2.22</p>

<p>Confidence Level: 0</p>

<p>AUTHORS: PMID:10092594,PMID:6151837</p>

<p>NOTES: </p>

</body>

</notes>

<listOfReactants>

<speciesReference species="M_arachd_x"/>

<speciesReference species="M_h_x"/>

<speciesReference species="M_coa_x"/>

</listOfReactants>

<listOfProducts>

<speciesReference species="M_arachdcoa_x"/>

<speciesReference species="M_h2o_x"/>

</listOfProducts>

<kineticLaw>

<math xmlns="<http://www.w3.org/1998/Math/MathML>">

<ci> FLUX_VALUE </ci>

</math>

<listOfParameters>

<parameter id="LOWER_BOUND" units="mmol_per_gDW_per_hr" value="0"/>

<parameter id="UPPER_BOUND" units="mmol_per_gDW_per_hr" value="1000"/>

<parameter id="FLUX_VALUE" units="mmol_per_gDW_per_hr" value="0"/>

<parameter id="OBJECTIVE_COEFFICIENT" units="dimensionless" value="0"/>

</listOfParameters>

</kineticLaw>

</reaction>

<reaction name="RE2995" id="R_RE2995X" metaid="_metaR_RE2995X" reversible="true" sboTerm="SBO:0000176">

<notes>

<body xmlns="<http://www.w3.org/1999/xhtml>">

<p>GENE_ASSOCIATION: (10455.1) or (1962.1) or (1891.1)</p>

<p>SUBSYSTEM: Fatty acid oxidation</p>

<p>EC Number: 5.3.3.8</p>

<p>Confidence Level: 0</p>

<p>AUTHORS: PMID:10407780,PMID:7775433</p>

<p>NOTES: </p>

</body>

</notes>

<listOfReactants>

<speciesReference species="M_CE4795_x"/>

</listOfReactants>

<listOfProducts>

<speciesReference species="M_CE5115_x"/>

</listOfProducts>

<kineticLaw>

<math xmlns="<http://www.w3.org/1998/Math/MathML>">

<ci> FLUX_VALUE </ci>

</math>

<listOfParameters>

<parameter id="LOWER_BOUND" units="mmol_per_gDW_per_hr" value="-1000"/>

<parameter id="UPPER_BOUND" units="mmol_per_gDW_per_hr" value="1000"/>

<parameter id="FLUX_VALUE" units="mmol_per_gDW_per_hr" value="0"/>

<parameter id="OBJECTIVE_COEFFICIENT" units="dimensionless" value="0"/>

</listOfParameters>

</kineticLaw>

</reaction>

<reaction name="RE2985" id="R_RE2985X" metaid="_metaR_RE2985X" reversible="false" sboTerm="SBO:0000176">

<notes>

<body xmlns="<http://www.w3.org/1999/xhtml>">

<p>GENE_ASSOCIATION: (8310.1) or (8309.1) or (51.1)</p>

<p>SUBSYSTEM: Fatty acid oxidation</p>

<p>EC Number: 1.3.3.6</p>

<p>Confidence Level: 0</p>

<p>AUTHORS: PMID:10407780,PMID:7775433</p>

<p>NOTES: </p>

</body>

</notes>

<listOfReactants>

<speciesReference species="M_arachdcoa_x"/>

<speciesReference species="M_o2_x"/>

</listOfReactants>

<listOfProducts>

<speciesReference species="M_CE4795_x"/>

</listOfProducts>

<kineticLaw>

<math xmlns="<http://www.w3.org/1998/Math/MathML>">

<ci> FLUX_VALUE </ci>

</math>

<listOfParameters>

<parameter id="LOWER_BOUND" units="mmol_per_gDW_per_hr" value="0"/>

<parameter id="UPPER_BOUND" units="mmol_per_gDW_per_hr" value="1000"/>

<parameter id="FLUX_VALUE" units="mmol_per_gDW_per_hr" value="0"/>

<parameter id="OBJECTIVE_COEFFICIENT" units="dimensionless" value="0"/>

</listOfParameters>

</kineticLaw>

</reaction>

<reaction name="RE2996" id="R_RE2996X" metaid="_metaR_RE2996X" reversible="true" sboTerm="SBO:0000176">

<notes>

<body xmlns="<http://www.w3.org/1999/xhtml>">

<p>GENE_ASSOCIATION: 26063.1</p>

<p>SUBSYSTEM: Fatty acid oxidation</p>

<p>EC Number: 1.3.1.34</p>

<p>Confidence Level: 0</p>

<p>AUTHORS: PMID:10407780,PMID:7775433</p>

<p>NOTES: </p>

</body>

</notes>

<listOfReactants>

<speciesReference species="M_CE4795_x"/>

<speciesReference species="M_h_x"/>

<speciesReference species="M_nadph_x"/>

</listOfReactants>

<listOfProducts>

<speciesReference species="M_CE5116_x"/>

<speciesReference species="M_nadp_x"/>

</listOfProducts>

<kineticLaw>

<math xmlns="<http://www.w3.org/1998/Math/MathML>">

<ci> FLUX_VALUE </ci>

</math>

<listOfParameters>

<parameter id="LOWER_BOUND" units="mmol_per_gDW_per_hr" value="-1000"/>

<parameter id="UPPER_BOUND" units="mmol_per_gDW_per_hr" value="1000"/>

<parameter id="FLUX_VALUE" units="mmol_per_gDW_per_hr" value="0"/>

<parameter id="OBJECTIVE_COEFFICIENT" units="dimensionless" value="0"/>

</listOfParameters>

</kineticLaw>

</reaction>

<reaction name="Transport reaction" id="R_r0931" metaid="_metaR_r0931" reversible="false" sboTerm="SBO:0000185">

<notes>

<body xmlns="<http://www.w3.org/1999/xhtml>">

<p>GENE_ASSOCIATION: </p>

<p>SUBSYSTEM: Transport, endoplasmic reticular</p>

<p>EC Number: </p>

<p>Confidence Level: 0</p>

<p>AUTHORS: PMID:11023036,PMID:12739169,PMID:12856180,PMID:12883891,PMID:1554704,PMID:18021224,PMID:4553030,PMID:793184,PMID:8725559,PMID:8781017</p>

<p>NOTES: </p>

</body>

</notes>

<listOfReactants>

<speciesReference species="M_arachd_l"/>

</listOfReactants>

<listOfProducts>

<speciesReference species="M_arachd_r"/>

</listOfProducts>

<kineticLaw>

<math xmlns="<http://www.w3.org/1998/Math/MathML>">

<ci> FLUX_VALUE </ci>

</math>

<listOfParameters>

<parameter id="LOWER_BOUND" units="mmol_per_gDW_per_hr" value="0"/>

<parameter id="UPPER_BOUND" units="mmol_per_gDW_per_hr" value="1000"/>

<parameter id="FLUX_VALUE" units="mmol_per_gDW_per_hr" value="0"/>

<parameter id="OBJECTIVE_COEFFICIENT" units="dimensionless" value="0"/>

</listOfParameters>

</kineticLaw>

</reaction>

<reaction name="Transport reaction" id="R_r0932" metaid="_metaR_r0932" reversible="true" sboTerm="SBO:0000185">

<notes>

<body xmlns="<http://www.w3.org/1999/xhtml>">

<p>GENE_ASSOCIATION: </p>

<p>SUBSYSTEM: Transport, lysosomal</p>

<p>EC Number: </p>

<p>Confidence Level: 0</p>

<p>AUTHORS: PMID:11023036,PMID:12739169,PMID:12856180,PMID:12883891,PMID:1554704,PMID:18021224,PMID:4553030,PMID:793184,PMID:8725559,PMID:8781017</p>

<p>NOTES: </p>

</body>

</notes>

<listOfReactants>

<speciesReference species="M_arachd_l"/>

</listOfReactants>

<listOfProducts>

<speciesReference species="M_arachd_c"/>

</listOfProducts>

<kineticLaw>

<math xmlns="<http://www.w3.org/1998/Math/MathML>">

<ci> FLUX_VALUE </ci>

</math>

<listOfParameters>

<parameter id="LOWER_BOUND" units="mmol_per_gDW_per_hr" value="-1000"/>

<parameter id="UPPER_BOUND" units="mmol_per_gDW_per_hr" value="1000"/>

<parameter id="FLUX_VALUE" units="mmol_per_gDW_per_hr" value="0"/>

<parameter id="OBJECTIVE_COEFFICIENT" units="dimensionless" value="0"/>

</listOfParameters>

</kineticLaw>

</reaction>

</listOfReactions>

</model>

</sbml>
