# Supplementary material for: Recon2Neo4j: applying graph database technologies for managing comprehensive genome-scale networks
Source: Bioinformatics. 2016 Dec 30;33(7):1096–8. doi: 10.1093/bioinformatics/btw731 (PMC5408918; doi:10.1093/bioinformatics/btw731)
Supplement: Supplementary Data [file btw731_supp.zip › Supplementary file 1 ΓÇô Data graph model for the metabolic framework.docx]

*Supplementary file 1 – Data graph model for the metabolic framework*

*
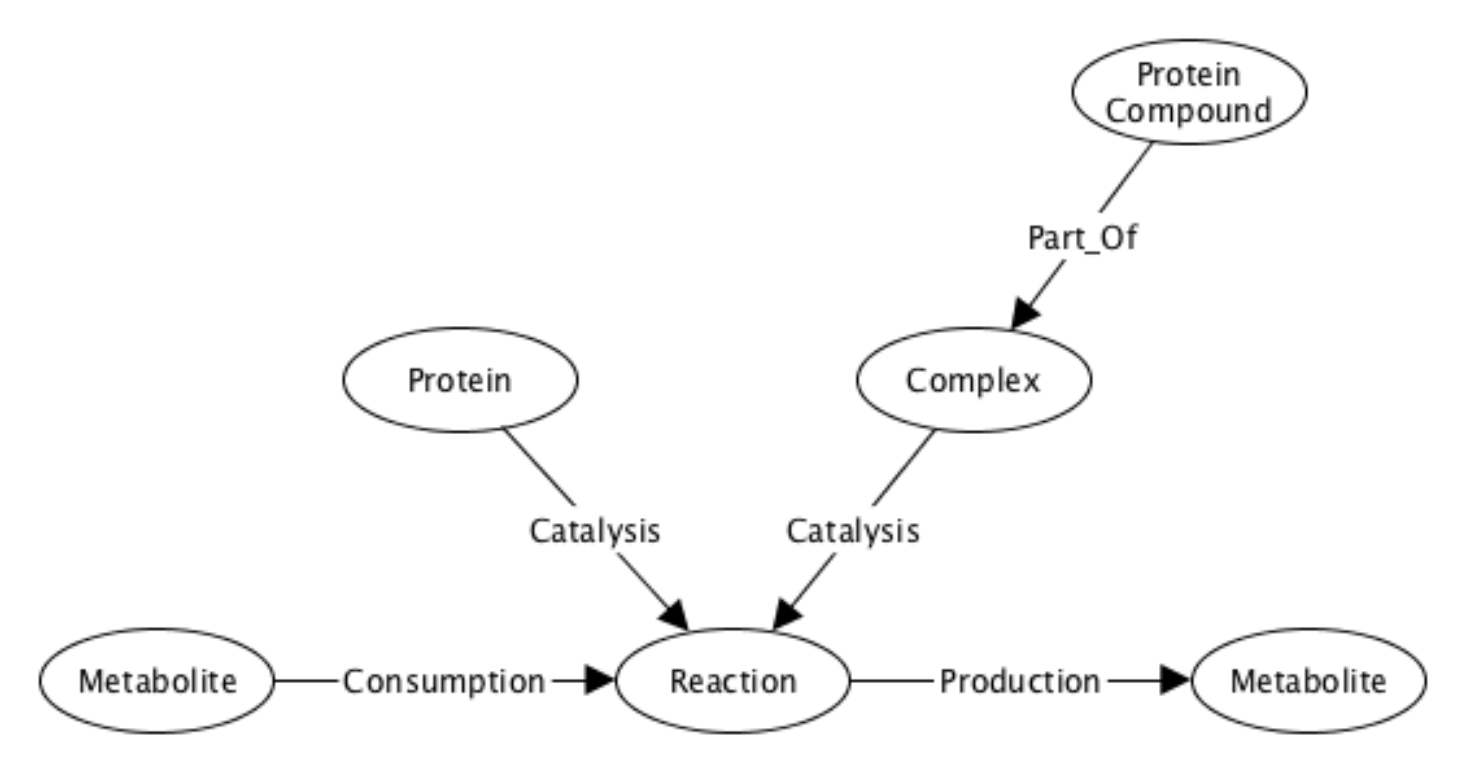
*

**Fig. S1. Data graph model**

Information is structured by graphs, with concepts represented as nodes (including metabolites, proteins, complexes, complexes and their compounds) that are connected by edges representing the metabolic reaction types (e.g. consumption, production, catalysis) or the involvement of the protein compounds into the biological complexes (the ‘part of’ associations).
