# Supplementary material for: Recon2Neo4j: applying graph database technologies for managing comprehensive genome-scale networks
Source: Bioinformatics. 2016 Dec 30;33(7):1096–8. doi: 10.1093/bioinformatics/btw731 (PMC5408918; doi:10.1093/bioinformatics/btw731)
Supplement: Supplementary Data [file btw731_supp.zip › Supplementary file 2 ΓÇô Overall workflow of the Recon2Neo4j framework.docx]

*Supplementary file 2 – Overall workflow of the Neo4-based metabolic framework and the parser for translating the JSON-based output into SBML and SIF formats*


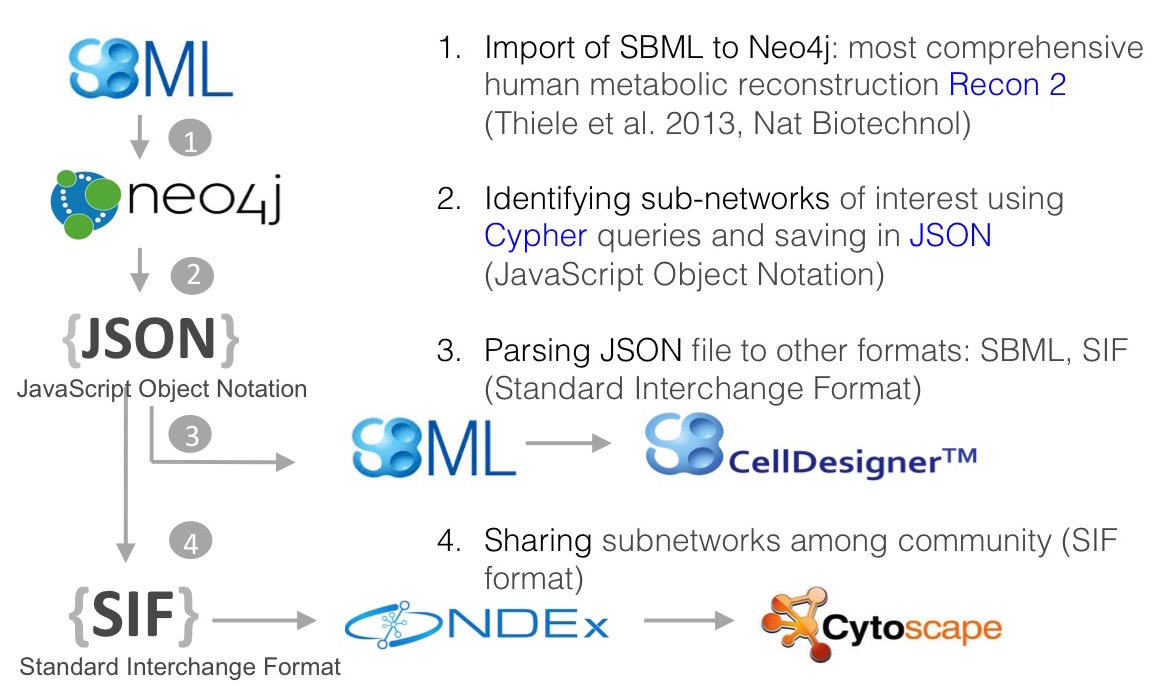


**Fig. S2. Graphical summary of the workflow of the Neo4-based metabolic framework and the parser for translating the JSON-based output into SBML and SIF formats**
